# Fish diversity and selection of taxa for conservation in the Salween and Irrawaddy Rivers, Southeast Asia

**DOI:** 10.1038/s41598-024-51205-5

**Published:** 2024-01-29

**Authors:** W. Zhou, X. Li

**Affiliations:** Key Laboratory for Conserving Wildlife with Small Populations in Yunnan, Southwest Forestry University, 300 Bailongsi Road, Kunming, 650224 Yunnan China

**Keywords:** Evolution, Zoology

## Abstract

Species diversity indices provide quantitative data for understanding the variations and trends in fish species diversity, as well as information on species richness and evenness. However, these diversity indices do not reflect differences in specific taxa, which can be of importance as key conservation targets, especially during the planning and construction of protected areas. In this study, simultaneously combining our improved traditional fish fauna analysis (TFFA) with the value of fish fauna presence (VFFP) methods, we studied fish diversity in the Salween and Irrawaddy basins. The results of the TFFA reflected the families (subfamilies) and genera that constitute the main body of fish diversity in the river basins. The results of the VFFP assessment showed which families (subfamilies) and genera were representative of certain characteristics in the basins. The VFFP scores of genera could be used as indicator indices and as priority taxa in the planning and construction of fish resource reserves. In this paper, we propose for the first time that the role and status of monotypic genera (genera comprising only a single species) in the conservation of fish diversity should not be ignored, and they should rather be a priority for protection.

## Introduction

Different biodiversity indices have been developed to reflect the characteristics of biodiversity at different levels. Four indices of species diversity, the species richness index of Margalef^1^, the Shannon–Wiener index^2^, the Simpson dominance index^3^ and the Pielou evenness index^4^, are commonly used indicators. Despite much criticism of these indices over the decades^5^, they are still widely used in fish diversity studies^6–12^. However, although these biodiversity indices are able to reflect the differences in diversity between/among study areas, they are unable to indicate which taxa are responsible for these differences. This information is of particular importance when planning and constructing protected areas, as it is necessary to decide which taxa should be selected as key conservation targets. Several studies have been concerned with the classification and conservation significance of monotypic genera (single species genera) in different biological groups^13–17^. However, there is to date no report on the selection of monotypic genera as conservation targets for freshwater fish in a river basin.

**Figure 1:**
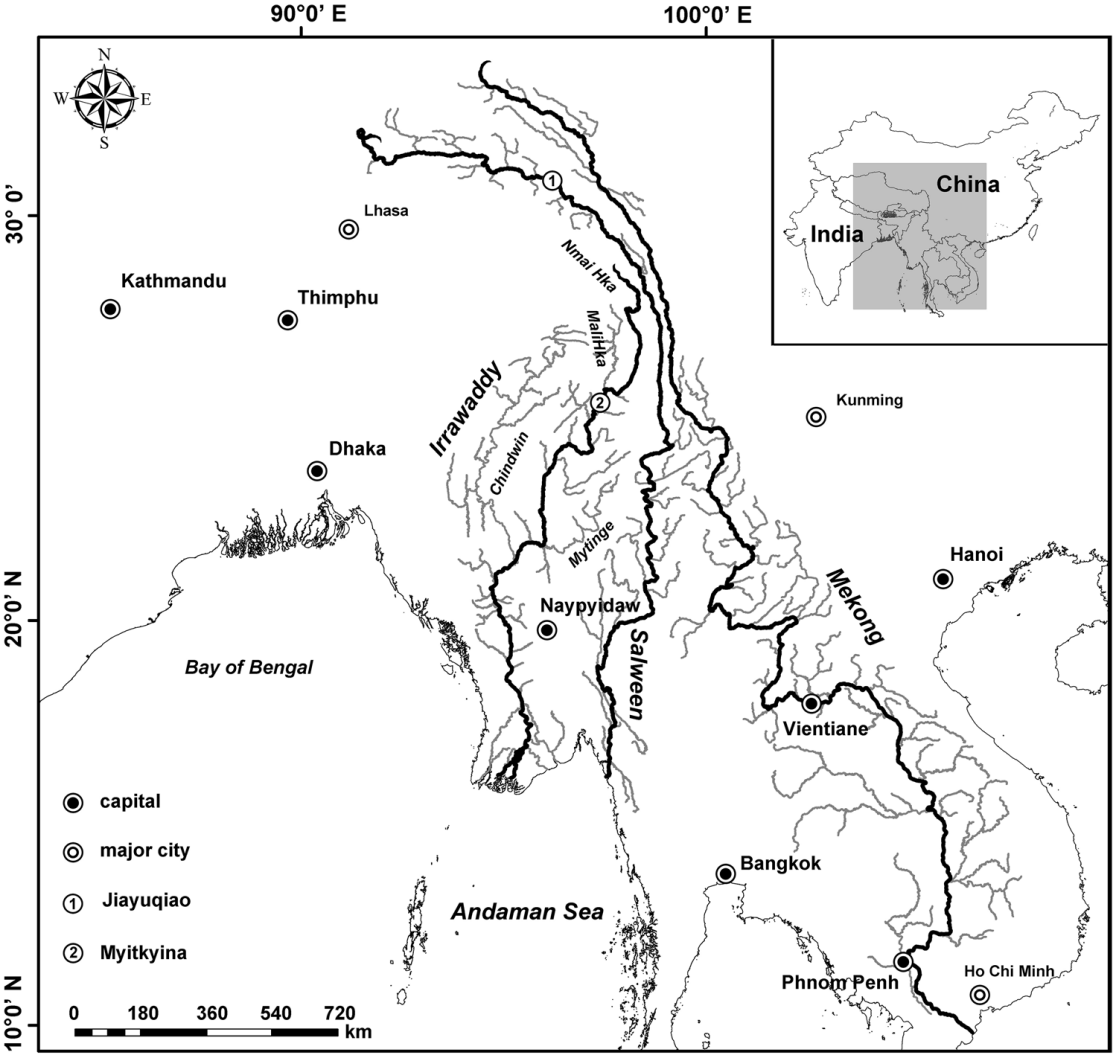
Three major rivers of Southeast Asia [The map of river basins was drawn based on online data provided by the National Platform for Common Geospatial Information Services (https://www.tianditu.gov.cn/), Ministry of Natural Resources of the People's Republic of China, with examination drawing number GS (2023) 336].

The Irrawaddy and the Salween are two major rivers of Southeast Asia (Fig. 1). A large number of studies have conducted classification and resources surveys of fish in the Salween and Irrawaddy River basins, and there are a great deal of survey data. Li et al.^18^ presented a list of fishes in the Irrawaddy River. They divided the Irrawaddy River basin into seven sub-basins based on the distribution patterns of 470 fish species and the impact of human activities. They suggested that at least four regions, the delta region, the main stem of the middle Irrawaddy, the Manipur River, and the upper Mali Hka River, should be incorporated into conservation planning. However, they did not indicate which taxa or species should receive protection. In contrast to the research in the Irrawaddy, the study of fishes along the whole of the Salween River basin has not been systematic to date, and has often been regional^19–26^ or has involved the classification of a taxon, or a description of a new species or genus^27–34^. Published papers and reports are scattered in various academic journals or books around the world and are extremely difficult to collect.

Zhou and Li^35,36^ proposed improved methods for the study of fish fauna. One method involves sorting of the absolute percentages of families or genera, with the sorted list being used to select the most representative families or genera that contributed the most to the fish composition. This is called the traditional fish fauna analysis (TFFA) method. Another method involves calculation of the ratio of the sub-taxa (genera or species) covered by a family or genus in the study area to all sub-taxa of the respective sub-taxa. If the ratio of a family or genus is higher, it indicates that the family or genus contains many (or all) sub-taxa in the study area, this family or genus will make a greater contribution to the fish diversity than a family or genus with a lower ratio. In this method, the contribution of a family or genus is small. This method is called the value of fish fauna presence (VFFP) method. Combining the traditional fish fauna analysis (TFFA) method with the value of fish fauna presence (VFFP) method, Zhou and Li^35,36^ investigated the fish composition of the Red River basin in Southeast Asia. The results of these TFFA and VFFP analyses were then used as indicators of fish diversity, and also as guiding indicators for the planning and construction of protected areas.

The Salween and Irrawaddy River basins are geographically close to South Asia, but the origin and differentiation of fish fauna in this area have not been reported so far. In this paper, we hope to integrate the scattered basic data of fish classification to form a complete list of fish in the Salween and Irrawaddy River basins. Firstly, we will discuss the origin and differentiation of fish fauna in the two basins. Secondly, we try to combine the results of TFFA and VFFP to propose taxa that should be prioritized for the conservation of fish diversity in each basin, so as to provide new ideas for the conservation of fish diversity in these two basins. Thirdly, we also want to verify the broad applicability of the TFFA and VFFP methods.

## Results

No including introduced fish species, a total of 362 native fish species belonging to 170 genera, 56 families, and 19 orders have been recorded in the Salween River (Supplementary Appendix 1-1), of which 12 genera were monotypic. Similarly, after the introduced fish species records were removed, records of a total of 502 species belonging to 193 genera, 66 families, and 22 orders were found from the Irrawaddy River (Supplementary Appendix 1-2), of which 13 genera were monotypic. Therefore, the fish taxon richness of the Irrawaddy River is greater and more differentiated than that of the Salween River (Tables S1-1, S1-2).

### Comparison of fish community composition between the Salween and Irrawaddy basins

In the Salween and Irrawaddy, the orders containing the highest diversity were concentrated in Cypriniformes, Siluriformes, Anabantiformes and Gobiiformes, and the most diverse families were found to be the following: Nemacheilidae, Cyprinidae, Danionidae, Bagridae and Sisoridae (Tables S2-1, S2-2).

There were orders, families, genera and species that were shared between the Salween and the Irrawaddy River basins, but that there were also taxa that were not shared. For example, 19 out of 22 orders found in the Irrawaddy were also found in the Salween River. Of the 502 species found in the Irrawaddy River, 189 were also recorded from the Salween, while 313 were not found in the Salween. The numbers of fish taxa (orders, families, genera and species) that were not shared between the Salween and Irrawaddy Rivers were much lower than the numbers of taxa common to both rivers (Table 1).

**Table 1:** Comparison of taxon distribution between the Salween and Irrawaddy basins.

	Family/subfamily			Genus			Species		
	Total taxa	Co-distributed taxa	Taxa found in only one river	Total taxa	Co-distributed taxa	Taxa found in only one river	Total taxa	Co-distributed taxa	Taxa found in only one river
Salween	71	70	1	170	138	32	362	189	173
Irrawaddy	80		10	193		55	502		313

### Ranking of families/subfamilies by two different methods

#### Ranking families/subfamilies based on the TFFA method

Twenty-two families/subfamilies were selected as the most highly representative families (subfamilies) contributing to the fish fauna of the Salween River basin using the criterion that the families (subfamilies) should contain five or more species. These families (subfamilies) contained a total of 272 species and 103 genera, and accounted for 30.99% of the total families (subfamilies), 60.59% of the total genera, and 75.14% of the total species in the Salween River. Nineteen taxa belonged to the Cypriniformes (11 families/subfamilies), the Siluriformes (six families/subfamilies), and the Anabantiformes (two families/subfamilies) (Table S3-1), which are the main groups of fish in the Salween River.

Similarly, a total of 30 families/subfamilies containing five or more species were selected as the most highly representative families (subfamilies) contributing to the fish fauna of the Irrawaddy River basin. The selected families (subfamilies) together comprised a total of 412 species and 122 genera, and accounted for 37.50% of the total families (subfamilies), 63.21% of the total genera, and 82.07% of the total species in the Irrawaddy River basin. Twenty-five taxa affiliated to the Cypriniformes (13 families/subfamilies), the Siluriformes (seven families/subfamilies), the Anabantiformes (two families/subfamilies), and the Gobiiformes (three families/subfamilies) (Table S3-2), which make up the main groups of fish in the Irrawaddy River.

Of the most representative families/subfamilies selected from the two basins, 20 taxa (family/subfamily) were distributed in both basins, although they did not feature in exactly the same sequence in both basins (Tables S3-1, S3-2).

#### Ranking families/subfamilies based on the VFFP method

A total of 28 fish families (subfamilies) found in the Salween River had a VFFP score greater than 40%, and eleven of these had a VFFP score of 100% (Table 2). These 28 selected families (subfamilies) contained a total of 123 species and 54 genera, accounting for 33.98% of the total species and 31.76% of the total genera found in the Salween. The number of species and genera made up about a third of the total species and genera found in the Salween River, however, none of the families (subfamilies) was endemic to the Salween River basin.

**Table 2:**
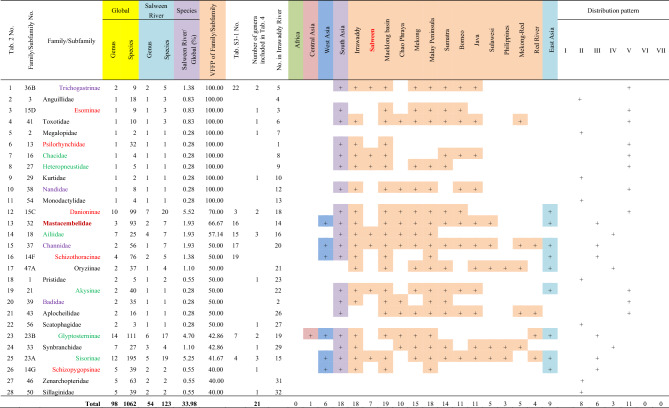
The families (subfamilies) with a Value of Fish Fauna Presence (VFFP) score greater than 40% in the Salween River and their distributions worldwide.

Family No. corresponds to the numbers in Supplementary Appendix 1-1. The family name is to the left of the cell, and the subfamily name is to the right. The five families (subfamilies) in red belong to the Cypriniformes. The six families (subfamilies) in green belong to the Siluriformes. The four families (subfamilies) in purple belong to the Anabantiformes. The family in dark red belongs to the Synbranchiformes. The 12 families (subfamilies) in black belong to other orders. A total of 26 families (subfamilies) are shared with the Irrawaddy fish fauna; these are shown in the column “No. in Irrawaddy River”. Global total species and genus is taken from Fricke et al.^45^. Distribution pattern: I-Pan-world distribution pattern; II-West Asia, South Asia, Southeast Asia, and East Asia distribution pattern; III-South Asia, Southeast Asia, and East Asia distribution pattern; IV-South Asia to Southeast Asia distribution pattern; V-Southeast Asia–East Asia distribution pattern; VI-Southeast Asia distribution pattern; VII-Salween River or Irrawaddy River distribution pattern.

There were 32 families (subfamilies) with a VFFP score greater than 40% in the Irrawaddy River, of which thirteen had VFFP scores of 100% (Table 3). These 32 selected families (subfamilies) included a total of 182 species and 66 genera, accounting for 36.25% of the total species and 34.20% of the total genera found in the Irrawaddy. The number of species and genera was represented more than a third of the total species and genera in the Irrawaddy River. However, none of the families (subfamilies) were endemic to the Irrawaddy River basin.

**Table 3:**
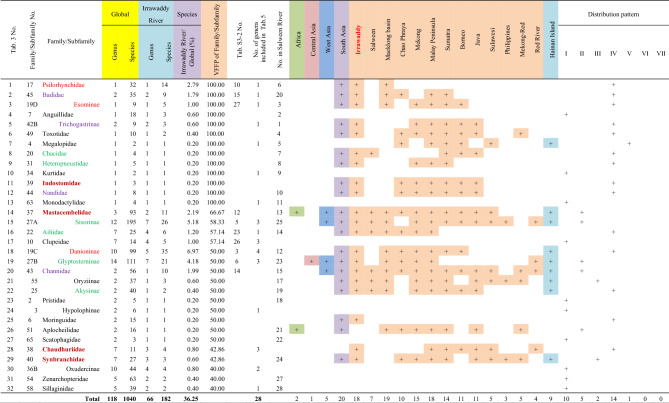
The families (subfamilies) with a Value of Fish Fauna Presence (VFFP) score greater than 40% in the Irrawaddy River and their distributions worldwide.

Family No. corresponds to the numbers in Supplementary Appendix 1-2. The family name is given to the left of the cell, and the subfamily name is to the right. The three families (subfamilies) in red belong to the Cypriniformes. The six families (subfamilies) in green belong to the Siluriformes. The four families (subfamilies) in purple belong to the Anabantiformes. The four families (subfamilies) in dark red belong to the Synbranchiformes. The 15 families (subfamilies) in black belong to other orders. A total of 26 families (subfamilies) are shared with the Salween River, shown in the column “No. in Salween River”. The distribution pattern definitions follow those given in Table 2.

Of the fish families (subfamilies) with a VFFP score greater than 40%, 26 taxa (family/subfamily) were found in the both river basins, although the taxa did not appear in exactly the same sequence in both basins. The subfamilies Schizothoracinae and Schizopygopsinae, which were both found in the Salween basin, did not appear in the list of selected taxa from the Irrawaddy basin (Table 2). Similarly, the families (subfamilies) of Indostomidae, Clupeidae, Hypolophinae, Moringuidae, Chaudhuriidae and Oxudercinae, found in the Irrawaddy basin, did not appear in the list of selected taxa from the Salween basin (Table 3).

### Ranking fish genera using two different methods

#### Ranking genera based on the TFFA method

Thirty-seven genera were selected as those most highly representative of the fish fauna of the Salween River basin using the criterion that they should contain three or more species (Table S4-1). These 37 genera together comprised 189 species, accounting for 52.21% of the total fish species and 21.76% of the total genera found in the Salween River. The selected genera belonged to the Cypriniformes (20 genera), the Siluriformes (10), the Anabantiformes (two), the Synbranchiformes (one), and the remaining five genera belonged to other orders. Twenty-seven of these also appeared in the thirty genera selected as representative of the Irrawaddy River basin fish fauna, but in a slightly different sequence. The remaining 10 genera did not appear in the list of selected genera of the Irrawaddy River basin, were not selected as representative of the Irrawaddy fauna as they featured low down in the sequence, or were not found at all in the Irrawaddy River basin.

A total of 30 genera, each with more than four species, were selected as representative of the Irrawaddy River basin fish fauna (Table S4-2). These 30 genera constituted a total of 277 species, accounting for 55.18% of the total fish species and 15.54% of the total genera in the Irrawaddy River basin. The orders represented included the Cypriniformes (18 genera), the Siluriformes (six), the Anabantiformes (three), and the Synbranchiformes (two), with the remaining genus belonging to another order. Twenty-two of these thirty genera were also represented in the thirty-seven genera selected from the Salween River basin fish fauna, but just in a slightly different sequence. A further eight genera did not appear in the 37 genera selected from the Salween River, or were not distributed in the Salween River basin at all.

#### Ranking genera based on the VFFP method

A total of 59 genera with VFFP scores greater than 30% were selected as those most representative of the fish fauna of the Salween River basin (Table 4). These findings were quite different from those identified using the TFFA method (Table S4-1). The 59 selected genera contained 107 species, accounting for 29.56% of the total fish species and 34.71% of the total genera in the Salween River basin. However, the species contained in these genera had distinct Salween characteristics. The genera belonged to the Cypriniformes (18 genera), the Siluriformes (11), the Gobiiformes (three), the Synbranchiformes (two) and the Anabantiformes (five). The remaining 20 genera belonged to other orders. When the results of the two methods were compared, of the top 37 genera selected using the TFFA method (Table S4-1), only eight genera had a VFFP score greater than 30% (Table S4-1, No. 2, 6, 17–18, 29–30, 33 and 36). The ranking results based on the VFFP score (Table 4) were thus quite different from those generated using the TFFA method (Table S4-1). Of these 59 genera with a VFFP score greater than 30%, 21 were included in 14 families (subfamilies) out of the 28 families (subfamilies) selected by the VFFP method, as shown in the column “Number of genera included in Table 4” in Table 2.

**Table 4:**
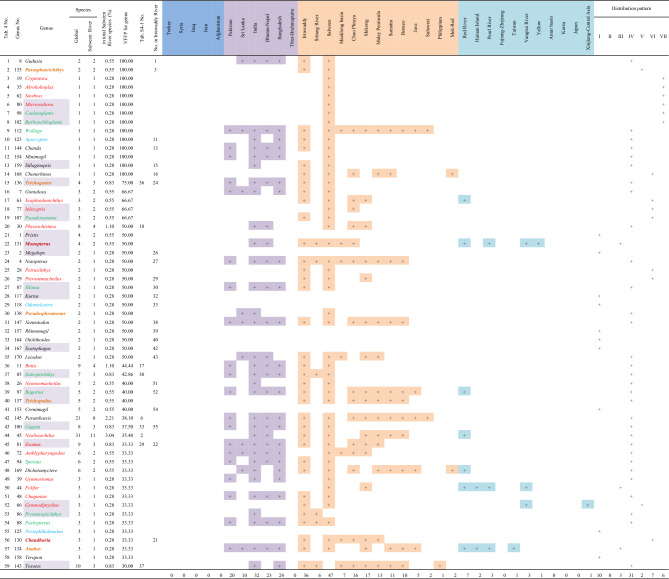
The genera with a VFFP score greater than 30% in the Salween River and their distributions worldwide.

Genus No. corresponds to the numbers in Supplementary Appendix 1-1. The taxa in red belong to the Cypriniformes, and comprise 18 genera. The taxa in green belong to the Siluriformes, and comprise 11 genera. The taxa in blue belong to the Gobiiformes, and comprise three genera. The taxa in dark red belong to the Synbranchiformes, and comprise two genera. The taxa in brownish yellow belong to the Anabantiformes, and comprise five genera. The taxa in black belong to other orders, together comprising 20 genera. Twenty-four genera are shared with the Irrawaddy River and are shown in the column “No. in Irrawaddy River”. The taxa with a light purple background, including *Microrasbora*, *Caelatoglanis*, *Barbeuchiloglanis*, and others, are also included in families with high VFFP scores (Table 2), totaling 21 genera. The distribution pattern definitions follow those given in Table 2.

A total of 57 representative genera with a VFFP score greater than 34% were selected from the Irrawaddy River basin (Table 5). These results were quite different from those found using TFFA (Table S4-2). These 57 genera constituted a total of 167 species, accounting for 33.27% of the total fish species and 29.53% of the total genera in the Irrawaddy River basin. However, the species included in these genera were representative species with distinct Irrawaddy characteristics. Thirty-seven genera were classified as the Cypriniformes (17 genera), the Siluriformes (eight), the Gobiiformes (six), the Anabantiformes (three), or the Synbranchiformes (three). Another 20 genera belonged to other orders. Comparing the two ranking methods, of the 30 genera selected by TFFA method (Table S4-2), only eleven had a VFFP score greater than 34% (Table S4-2, No. 3, 4, 6, 9, 11–12, 14, 16, 22, 28, 30 and 37). The ranking results based on the VFFP score (Table 5) were significantly different from those obtained using the TFFA method (Table S4-2). Thirteen of the 57 VFFP-selected genera were monotypic (Table 4, No. 4–16). Of the 57 genera selected using VFFP as being representative of the Irrawaddy fish fauna, 28 were included in 15 families (subfamilies) out of the 32 families (subfamilies) selected by the VFFP method, as shown in column “Number of genera included in Table 5” in Table 3.

**Table 5:**
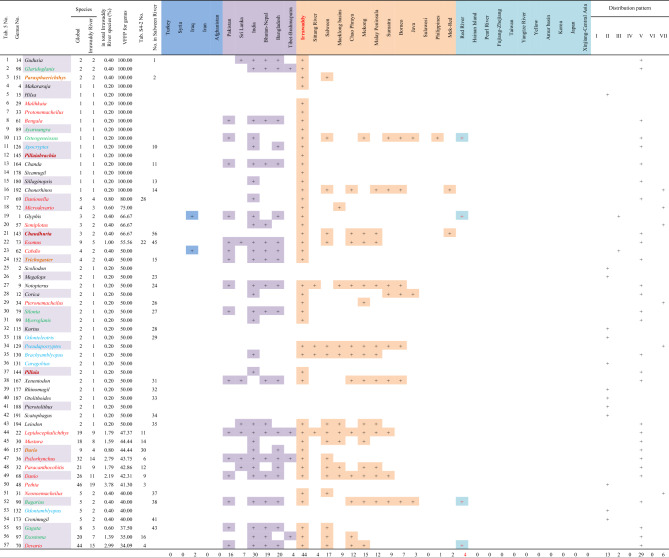
The genera with a VFFP score greater than 34% in the Irrawaddy River and their distributions worldwide.

Genus No. corresponds to the numbers in Supplementary Appendix 1-2. The taxa in red belong to the Cypriniformes, and comprise 17 genera. The taxa in green belong to the Siluriformes, and comprise 8 genera. The taxa in blue belong to the Gobiiformes, and comprise six genera. The taxa in dark red belong to the Synbranchiformes, and comprise three genera. The taxa in brownish yellow belong to the Anabantiformes, and comprise five genera. The taxa in black belong to other orders. Twenty-four genera are shared with the Salween River; these are shown in the column “No. in Salween River”. The taxa with a light purple background, including *Gudusia*, *Glaridoglanis*, *Makararaja*, and others, are also included in families with high VFFP scores (Table 3), totaling 28 genera. The distribution pattern definitions follow those given in Table 2.

Of these selected genera with high VFFP scores, twenty-four were common to both river basins, though they were placed in slightly different sequences. In the Salween River basin, thirty-five genera did not appear in the 57 genera selected as representative of the Irrawaddy (Table 4). Conversely, thirty-three genera found in the Irrawaddy did not appear in the 59 genera selected as representative of the Salween (Table 5). In both the Salween and the Irrawaddy River basins, about half of the fish genera with high VFFP scores came from families representing only about half the families (subfamilies) with high VFFP scores. This indicates that there was no significant correlation between the high-scoring genera selected by the VFFP method and the high-scoring families (subfamilies) selected by the same method.

In total, there were 21 monotypic genera in the two basins, and their VFFP values were all 100% (Table 4, No. 3–14; Table 5, No. 4–16). Of these monotypic genera, only four, *Apocryptes*, *Chanda*, *Sillaginopsis*, and *Chonerhinos*, were shared by the two basins, with the other monotypic genera being endemic to one of other basin. None of the monotypic genera was found in other regions of the world (Table 6). In addition to the monotypic genera, there were three genera, *Gudusia*, *Glaridoglanis* and *Parasphaerichthys*, which comprised only two species each. The species in the genera *Gudusia* and *Parasphaerichthys* were found in both the Salween and Irrawaddy River basins (Table 5, No. 1–3). However, the two species of the genus *Glaridoglanis* were found only in the Irrawaddy River basin (Table 6).

**Table 6:** Comparison of distributions of monotypic genera between the Irrawaddy and Salween rivers.

No.	Genus	Distribution	
		Irrawaddy	Salween
Genus (monotypic)			
1	*Makararaja*	+	
2	*Hilsa*	+	
3	*Malihkaia*	+	
4	*Protonemacheilus*	+	
5	*Cryptotora*		+
6	*Akrokolioplax*		+
7	*Sawbwa*		+
8	*Bengala*	+	
9	*Microrasbora*		+
10	*Ayarnangra*	+	
11	*Caelatoglanis*		+
12	*Barbeuchiloglanis*		+
13	*Wallago*		+
14	*Osteogeneiosus*	+	
15	*Apocryptes*	+	+
16	*Pillaiabrachia*	+	
17	*Chanda*	+	+
18	*Minimugil*		+
19	*Sicamugil*	+	
20	*Sillaginopsis*	+	+
21	*Chonerhinos*	+	+
	Total	13	12
Genus (comprising 2 species)			
1	*Gudusia*	+	+
2	*Glaridoglanis*	+	
3	*Parasphaerichthys*	+	+
	Total	3	2

The genus names are listed in the sequence they appear in Supplementary Appendix 1-1 and Supplementary Appendix 1-2.

### Distribution patterns

The VFFP scores of fish families (subfamilies) and genera in the Salween and Irrawaddy River basins (Tables 2, 3, 4, 5) were used to obtain their distribution patterns (Table 7). The characteristics of the distribution patterns were as follows.

1. The South Asia to Southeast Asia distribution pattern (IV) was the largest and most common of the distribution patterns. Of the 28 families/subfamilies with high VFFP scores in the Salween River, eleven had distribution pattern IV (Table 2, No. 1, 3, 4, 6–8, 10, 12 and 19–21). Of the 32 families/subfamilies with high VFFP scores in the Irrawaddy, fourteen had distribution pattern IV (Table 3, No. 1–3, 5, 6, 8, 9, 11, 12, 16, 18, 22, 25 and 28). There were 31 genera with this distribution pattern in the Salween River basin (Table 4, No. 1, 9–13, 15, 16, 20, 24, 27, 30, 31, 35–40, 42–49, 51, 53, 54 and 59), and 29 genera with this pattern in the Irrawaddy (Table 5, No. 1, 2, 8, 10, 11, 13, 15, 17, 21, 22, 24, 27, 28, 30, 31, 35, 37, 38, 43–49, 52 and 55–57).
2. The second most common distribution pattern was the pan-world distribution pattern (I). A total of eight families/subfamilies showed this pattern in the Salween River basin (Table 2, No. 2, 5, 9, 11, 18, 22, 27 and 28), as well as ten genera (Table 4, No. 21, 23, 28, 29, 32–34, 41, 55 and 58). A total of ten families/subfamilies with this pattern were found in the Irrawaddy (Table 3, No. 4, 10, 13, 17, 23, 24, 27 and 30–32), as well as 13 genera (Table 5, No. 5, 25, 26, 32, 33, 36, 39–42, 50, 53 and 54). These taxa required marine, brackish and freshwater habitats.
3. There were no fish families (subfamilies) endemic to the Salween or to the Irrawaddy, and therefore no families (subfamilies) with distribution pattern VII. However, six genera endemic to the Salween (Table 4, No. 3–8) and seven genera endemic to the Irrawaddy (Table 5, No. 3, 4, 6, 7, 9, 12 and 14) showed distribution pattern VII.
4. There were no families (subfamilies) endemic to Southeast Asia found in the Salween or the Irrawaddy, and therefore no families (subfamilies) with distribution pattern VI. However, seven genera found in the Salween River (Table 4, No. 14, 17–19, 25, 26 and 56), and six genera found in the Irrawaddy (Table 5, No. 16, 18, 20, 29, 34 & 51) showed distribution pattern VI.
5. The Irrawaddy River had no genera that were also found in East Asia, while the Salween River had three (Table 4, No. 50, 52 and 57).

**Table 7:**
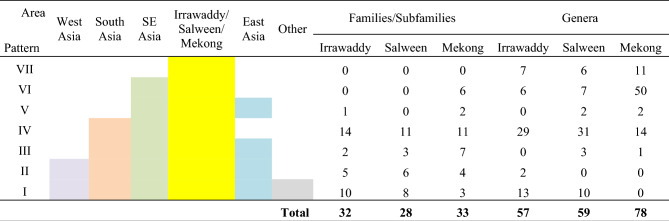
Comparison of distribution patterns of fish families (subfamilies) and genera among the Irrawaddy, Salween and Mekong Rivers.

Each horizontal succession of colored squares in the table represents a distribution pattern.

The data for the Irrawaddy and Salween Rivers is based on that presented in Tables 2, 3, 4, 5, and the data pertaining to the Mekong River was taken from Zhou and Li^35,36^.

## Discussion

### Origin and differentiation of fish fauna of the Salween and Irrawaddy Rivers

Although the Salween and Irrawaddy basins are geographically close to South Asia, their fish diversity was not dominated by that of South Asia. The distribution pattern with the highest representation in the fish families (subfamilies) and genera of the Salween and Irrawaddy basins was the South Asia-Southeast Asia distribution pattern (IV). If the genera with pattern VII (those genera endemic to the Salween and Irrawaddy basins) and with type VI (those taxa found in the Salween and Irrawaddy basins together with Southeast Asia) were added (Table 7), the influence of Southeast Asian patterns of fish diversity in these two basins becomes very clear. However, there is no doubt that the fish diversity of these two basins is also characteristic of the South Asian fish fauna. This result is consistent with the fish diversity of the Mekong River and Red River drainages^35,36^. Therefore, the distribution pattern of fish families (subfamilies) and genera is a good index to analyze the origin of fish diversity. Geological data show that the suture of the collision between the Eurasian plate and the Indian plate is located in the upper reaches of the Brahmaputra River and extends southwards to the Gaoligong Mountains^37–39^. Our results on fish fauna composition in the Salween and Irrawaddy basins agree with this. After the collision of the two plates, the Eurasian plate overlay the Indian plate, so the fish of Southeast Asia became the dominat fish fauna in the region, and then the fish of the Indian plate spread and penetrated into the major rivers of Southeast Asia. The taxa shared between the Salween and the Irrawaddy basins are the evidence that these areas used to be connected, while the taxa unique each basin are the result of their reduced connectivity after the separation of the two basins, and the subsequent divergence of the taxa.

### TFFA results showed specific taxa with rich fish diversity

The TFFA calculation (Tables S3-1, S3-2, S4-1, S4-2) in this study was able to interpret taxa that contribute most to fish diversity well. In several large river basins in Asia, such as the Yangtze, Pearl, Red and Mekong, almost all fishes fall into the Cypriniformes and the Siluriformes^35,36,40–44^, and this is reflected in the results from the Salween and the Irrawaddy (Tables S1, S2). The fish fauna in these basins thus reflected the general fish fauna in Asia. However, these results can only explain the proportion and contribution of different taxa (orders, families or genera) to the overall composition of the fish fauna in a particular basin, and do not reflect which taxa (orders, families or genera) are particularly representative of a particular basin, nor can they reflect the diversity and fundamental differences in fish composition from other basins.

### VFFP results showed the significance of monotypic genera

The status of monotypic genera in fish diversity conservation should not be ignored as monotypic taxa often have rather narrow distributions and are confined to the basins being studied or the adjacent regions. According to Eschmeyer's catalog of fishes^45^, the distribution of these monotypic genera is very narrow. Vargas^17^ suggested the concept of “endangered living fossils” (ELFs), integrating the highly endangered status and evolutionary singularity of any species, and proposed that monotypic genera met three ELF criteria, one of which was scarcity and narrow distribution of the population. The Salween and Irrawaddy River basins have a very high proportion of monotypic genera, with several of these representing unique species and genera endemic to the basins studied (Table 6). Once these become extinct, both the species and the genetic information they carry is lost forever. Therefore, their role in the ecosystems and their contribution to the diversity of fish species in their respective catchments should not be ignored, and neither should their conservation.

In addition to the monotypic genera, there were three genera, *Gudusia*, *Glaridoglanis* and *Parasphaerichthys*, which comprised only two species each. As a result, these genera are also excellent indicators of the diversity of fish in the two basins.

### VFFP scores used as indicators to reflect the characteristics of fish diversity and to identify key conservation targets

The VFFP analysis of genera was a good indicator of the differences in fish diversity between the Salween and the Irrawaddy, and also reflected the characteristics of fish diversity and the key conservation targets of the two basins.

The number of families and genera selected by the VFFP method represented only about a third of the total number of genera and species in the Salween and Irrawaddy River basins (Tables 2, 3, 4, 5). Most genera and species found in the Salween or the Irrawaddy had a distribution that covered the Salween, the Irrawaddy, and parts of Southeast Asia, and the Southeast Asian fish fauna reflected the characteristics of the fish fauna and diversity in the Salween and the Irrawaddy. However, the VFFP results for the families (subfamilies) (Tables 2 and 3) did not reflect the differences in fish diversity between the Salween and Irrawaddy Rivers. Of the selected representative and distinctive families (subfamilies), 26 families (subfamilies) were common to both river basins, while only two of the families (subfamilies) in the Salween River basin were not found in the Irrawaddy, while six families (subfamilies) from the Irrawaddy River basin were not found in the Salween. Therefore, the results of the VFFP analysis at the family (subfamily) level were not indicative of the differences in fish diversity between the two basins.

The VFFP results at the genus level were, however, able to reflect the differences in fish diversity between the Salween and Irrawaddy basins well (Tables 4 and 5). Of the selected representative and distinctive genera, 24 were common to the two basins, while 35 genera found in the Salween River basin were not found in the Irrawaddy (Table 4), and 33 genera from the Irrawaddy River basin were not found in the Salween (Table 5). The VFFP score for each genus could therefore potentially be used as an indicator to determine the conservation priority of that genus.

The VFFP scores could be used as a reference index for the planning and construction of fish reserves. It is difficult to make a conservation plan for the whole basin of the Salween or the Irrawaddy as fish reserves, however, it is possible to consider some of the tributaries or sections (reaches) of each river as fish reserves. The more genera with high VFFP scores in the selected tributaries or river sections, the greater the number of characteristic and representative taxa of the river would be protected were this area a reserve, and thus the higher the conservation value of reserves in these areas. Moreover, since the habitat requirements of these indicator taxa would also cover those of other genera and species, the protection of these indicator taxa would also provide an umbrella, benefiting other taxa and in turn, the whole ecosystem^35,46^.

## Materials and methods

### Study area

The Irrawaddy River basin is located between 15°30′–28°50′ N and 93°16′–98°42′ E (Fig. 1), and the source comprises both an eastern and a western branch. The eastern source, the Nmai Hka River, originates in the southwestern foot of the Boshula Mountains in Zayo County, Tibet, China, with a maximum elevation of 5881 m^47^. The western source, the Mailikai River, originates in the northern mountains of Myanmar. It is called the Irrawaddy (Burmese Ayeyarwady) after the two rivers meet at Myitsone, about 45 km north of Myitkyina. The Irrawaddy runs north to south through Myanmar, through the mountainous northern region, the dry central region and the southern delta (about 30,000 km^2^). It finally divides into the multiple branches of its distributaries and empties into the Andaman Sea in the Indian Ocean. The total length of the Irrawaddy is 2714 km, and it has a drainage area of about 410,000 km^2^. The Chindwin River is a main tributary of the Irrawaddy in northern Myanmar. The Chindwin is formed in the Pātkai and Kumon ranges of the Indo-Myanmar border by a network of headstreams including the Tanai, Tawan, and Taron. About 10 km below Myingyan, the Chindwin empties into the Irrawaddy^47–49^.

The Salween basin is located between 23°05′–32°48′N and 91°10′–100°15′E (Fig. 1). The river originates from the Jigegepa Mountains at the southern foot of the Tanggula Mountains on the Tibetan Plateau, China, and its source is the 5450 m Jiangmei Ergangdolou Glacier. From the source to Jiayuqio in Qamdo, Tibet, the Salween is called the Nagqu. This part of the river is located in the Tibetan plateau area, a relatively flat mountain landscape, where the river is mainly supplemented by snow and ice. In this area, the river has high water flow, the river bed is wide, and the velocity is slow. From Jiayuqiao in Tibet to Mangshi City in Yunnan, China, the river is called the Nu-Jiang. The section from Jiayuqiao in Tibet to Liuku in Yunnan has a steep gradient and the water runs fast, through high mountains and deep valleys. From Liuku to Mangshi, a large amount of rain is added into the Nu-Jiang River, and the mountains are open, forming an extensive agricultural area. The river passes through Tibet and Yunnan, and enters Myanmar at Mangshi City. In Myanmar, the river is called the Salween. The Salween forms the Myanmar-Thailand border across the Shan Plateau, and finally reaches the Andaman Sea in the Indian Ocean near Mawlamian (formerly Moulmein) in Myanmar. Throughout its length, the Salween runs between the Tenasserim Hills or Tenasserim Range (the upper part of which is called the Nu Mountains in Yunnan) and the Gaoligong Mountains. The length of the main stream of the Salween is 3680 km, and the basin covers an area of 325,000 km^2^. The length of the main stream in China is 2020 km, and the drainage area in China is 137,000 km^2^. The length of the main stream in Myanmar is 1540 km, with a drainage area of 170,000 km^2^. The length of the section of river forming the Myanmar-Thailand boundary is 120 km, and the drainage area in Thailand is 18,000 km^2  ^^48,50–52^.

### List of fish species

No new experiments were conducted for this study, but our previous experiments were conducted in China and comply with the current laws of the country in which they were performed. All experiments were carried out under the Institutional Animal Care and Use Committee (SWFU L20161211) at Southwest Forestry University. The list of fish of the Salween and Irrawaddy River basins presented here is derived from the fish specimens preserved in the Museum of Southwest Forestry University since the 1980s, together with a comprehensive literature survey^19–24,53–63^, and a total of 181 scientific articles and other documents retrieved from Eschmeyer's catalog of fishes: genera, species, and references therein^64^, and including fish taxonomic studies from the 1990s to 2022 (Supplementary Reference). The list of fish includes only living native fish species, and excludes fossil and introduced species, because the introduced species would interfere with the results of the fauna analysis of the living fish.

In this study, families and genera are used as the basic statistical and analytical units. If a family can be divided into subfamilies, the subfamily is used as the statistical unit. We classified the fish species into order and family/subfamily following Fricke et al.^64^. The division of the Cypriniformes follows Mayden et al.^65^, Saitoh et al.^66^, Tang et al.^67^, Kottelat^55^, Yang et al.^68^, and Tan and Armbruster^69^. The validity of species refers to the latest taxonomic data published by Fricke et al.^45^ and Froese and Pauly^70^, and the ranking of orders and families follows Fricke et al.^64^. The genera and species were arranged alphabetically. The distribution of species follows Fricke et al.^64^, and Froese and Pauly^70^.

### Calculations

#### Ranking by absolute number of included species: the TFFA method

The absolute percentage of the total of each family or genus in the study area was calculated using the TFFA (the traditional fish fauna analysis) method. However, because families (subfamilies) and genera comprised different numbers of genera and species, respectively, their contributions to fish composition were not equal. Using the method proposed by Zhou and Li^35,36^, the taxa with the highest contribution were selected by sorting the fish in the study area according to the number of species contained in the families (subfamilies) or genera. The selection principles for taxa were as follows. (1) The number of selected families (subfamilies) accounted for about 60% of the total number of families (subfamilies) in the study area, or the number of species included in the selected families (subfamilies) should account for 70% of the total number of species in the study area. (2) The number of species in the selected genus should account for about 50% of the total species in the study area. (3) When the specified percentage was reached or exceeded, if several families (subfamilies) or genera had an equal number of species, they were simultaneously selected or rejected. (4) The selected families (subfamilies) and genera were ranked according to the number of genera or species included. If the number of genera or species was equal, the sequence of families (subfamilies) and genera was determined from fish list of the basin.

#### Ranking by frequency of secondary taxa: the VFFP method

In fact, the families (subfamilies) or genera themselves did not contribute equally to the taxon diversity of the study area. Some families (subfamilies) or genera were represented by all their secondary taxa (genera or species, respectively) in the study area, while other families (subfamilies) or genera were represented by only few of their secondary taxa. In this study, the VFFP (value of fish fauna presence) method was used to describe this difference in contribution^35,36^. The formula is as follows:

$${\text{VFFP}} = \, \left( {n/m} \right) \, \times { 1}00\%$$

where *n* refers to the number of secondary taxa that appear in the target region and *m* refers to the total number of secondary taxa globally.

To ensure that the families (subfamilies) or genera selected according to VFFP scores were representative and regionally characteristic, the principles proposed by Zhou and Li^35,36^ were adopted: (1) The number of families (subfamilies) selected should only be approximately 30% of the total number of families (subfamilies) in the study area, or these families (subfamilies) should have a VFFP score equal to or greater than 40%. (2) The number of genera selected should only be approximately 25% of the total number of genera in the study area, or should be genera with a VFFP score equal to or greater than 50%. (3) The selected families (subfamilies) or genera were ranked based on their VFFP scores. (4) If the VFFP score was equal across several families (subfamilies) or genera, these would be ranked by the number of their secondary genera or species. If the number of genera or species was also equal among families or genera, the sequence of the families (subfamilies) or genera would be determined using the fish list of the basin (Supplementary Appendix 1–1 and 1–2).

### Division of distribution pattern

The distribution of a genus is a superposition of the distributions of all of its species. Similarly, the distribution of a family (subfamily) is a superposition of the distributions of all of its genera. The distributions of genera or families (subfamilies) found in the study area differ between taxa. Some have a narrow distribution and are limited to the study area, some of them are also distributed in adjacent regions, and others are widely distributed, and are found in multiple regions in Asia, or even have global distribution. Following the method of Zhou and Li^35,36^, and according to the distributions of families (subfamilies) or genera, the following seven distribution patterns were defined:

I. Pan-world distribution pattern.
II. West Asia, South Asia, Southeast Asia, and East Asia distribution pattern.
III. South Asia, Southeast Asia, and East Asia distribution pattern.
IV. South Asia to Southeast Asia distribution pattern.
V. Southeast Asia–East Asia distribution pattern.
VI. Southeast Asia distribution pattern.
VII. Salween River or Irrawaddy River distribution pattern. A special pattern separate from that of Southeast Asia.

### Supplementary Information

Supplementary Information 1.

Supplementary Information 2.

Supplementary Information 3.

Supplementary Table S1.

Supplementary Table S2.

Supplementary Table S3.

Supplementary Table S4.

## Data Availability

All data generated or analyzed during this study are included in this published article [and its supplementary information files].
