# Supplementary material for: Fish diversity and selection of taxa for conservation in the Salween and Irrawaddy Rivers, Southeast Asia
Source: Sci Rep. 2024 Jan 29;14:2393. doi: 10.1038/s41598-024-51205-5 (PMC10825156; doi:10.1038/s41598-024-51205-5)
Supplement: Supplementary file 1 — Supplementary Information 1. [file 41598_2024_51205_MOESM1_ESM.docx]

#### Supplementary Appendix 1-1 List of fish species known from the Salween River

| Family No. | Genus No. | Taxa (order, family, subfamily, genus, and species) | Family/subfamily | | Species in the genus |
| --- | --- | --- | --- | --- | --- |
|  |  |  | Genera | Species |  |
|  |  | **Elasmobranchii** |  |  |  |
|  |  | 1. RHINOPRISTIFORMES |  |  |  |
| 1 |  | Pristidae | 1 | 2 |  |
|  | 1 | *Pristis pectinata* Latham 1794 |  |  | 2 |
|  |  | *Pristis zijsron* Bleeker 1851 |  |  |  |
|  |  | **Actinopteri** |  |  |  |
|  |  | 2. ELOPIFORMES |  |  |  |
| 2 |  | Megalopidae | 1 | 1 |  |
|  | 2 | *Megalops cyprinoides* (Broussonet 1782) |  |  | 1 |
|  |  | 3. ANGUILLIFORMES |  |  |  |
| 3 |  | Anguillidae | 1 | 3 |  |
|  | 3 | *Anguilla bengalensis* (Gray 1831) |  |  | 3 |
|  |  | *Anguilla bicolor* McClelland 1844 |  |  |  |
|  |  | *Anguilla nebulosa* McClelland 1844 |  |  |  |
|  |  | 4. OSTEOGLOSSIFORMES |  |  |  |
| 4 |  | Notopteridae | 1 | 1 |  |
|  | 4 | *Notopterus notopterus* (Pallas 1769) |  |  | 1 |
|  |  | 5. CLUPEIFORMES |  |  |  |
| 5 |  | Engraulidae | 2 | 3 |  |
|  | 5 | *Coilia reynaldi* Valenciennes 1848 |  |  | 1 |
|  | 6 | *Setipinna phasa* (Hamilton 1822) |  |  | 2 |
|  |  | *Setipinna wheeleri* Wongratana 1983 |  |  |  |
| 6 |  | Clupeidae | 2 | 4 |  |
|  | 7 | *Gonialosa modesta* (Day 1870) |  |  | 2 |
|  |  | *Gonialosa whiteheadi* Wongratana 1983 |  |  |  |
|  | 8 | *Gudusia chapra* (Hamilton 1822) |  |  | 2 |
|  |  | *Gudusia variegata* (Day 1870) |  |  |  |
| 7 |  | Pristigasteridae | 2 | 3 |  |
|  | 9 | *Ilisha megaloptera* (Swainson 1838) |  |  | 2 |
|  |  | *Ilisha novacula* (Valenciennes 1847) |  |  |  |
|  | 10 | *Pellona ditchela* Valenciennes 1847 |  |  | 1 |
|  |  | 6. CYPRINIFORMES |  |  |  |
| 8 |  | Botiidae |  |  |  |
|  |  | Botiinae | 2 | 5 |  |
|  | 11 | *Botia almorhae* Gray 1831 |  |  | 4 |
|  |  | *Botia histrionica* Blyth 1860 |  |  |  |
|  |  | *Botia kubotai* Kottelat 2004 |  |  |  |
|  |  | *Botia rostrata* Günther 1868 |  |  |  |
|  | 12 | *Syncrossus berdmorei* Blyth 1860 |  |  | 1 |
| 9 |  | Cobitidae | 5 | 11 |  |
|  | 13 | *Acantopsis dialuzona* van Hasselt 1823 |  |  | 2 |
|  |  | *Acantopsis spectabilis* (Blyth 1860) |  |  |  |
|  | 14 | *Acanthopsoides delphax* Siebert 1991 |  |  | 1 |
|  | 15 | *Lepidocephalichthys alkaia* Havird & Page 2010 |  |  | 5 |
|  |  | *Lepidocephalichthys berdmorei* (Blyth 1860) |  |  |  |
|  |  | *Lepidocephalichthys goalparensis* Pillai & Yazdani 1976 |  |  |  |
|  |  | *Lepidocephalichthys guntea* (Hamilton 1822) |  |  |  |
|  |  | *Lepidocephalichthys hasselti* (Valenciennes 1846) |  |  |  |
|  | 16 | *Misgurnus anguillicaudatus* (Cantor 1842) |  |  | 1 |
|  | 17 | *Pangio fusca* (Blyth 1860) |  |  | 2 |
|  |  | *Pangio oblonga* (Valenciennes 1846) |  |  |  |
| 10 |  | Balitoridae | 5 | 9 |  |
|  | 18 | *Balitora brucei* Gray 1830 |  |  | 3 |
|  |  | *Balitora burmanica* Hora 1932 |  |  |  |
|  |  | *Balitora nantingensis* Chen, Cui & Yang 2005 |  |  |  |
|  | 19 | *Cryptotora thamicola* (Kottelat 1988) |  |  | 1 |
|  | 20 | *Hemimyzon nujiangensis* (Zhang & Zheng 1983) |  |  | 2 |
|  |  | *Hemimyzon tchangi* (Zheng 1982) |  |  |  |
|  | 21 | *Homaloptera bilineata* Blyth 1860 |  |  | 1 |
|  | 22 | *Homalopteroides modestus* (Vinciguerra 1890) |  |  | 2 |
|  |  | *Homalopteroides rupicola* (Prashad & Mukerji 1929) |  |  |  |
| 11 |  | Nemacheilidae | 10 | 36 |  |
|  | 23 | *Homatula anteridorsalis* Li, Che & Zhou 2019 |  |  | 3 |
|  |  | *Homatula cryptoclathrata* Li, Che & Zhou 2019 |  |  |  |
|  |  | *Homatula nigra* Li, Che & Zhou 2019 |  |  |  |
|  | 24 | *Mustura maepaiensis* (Kottelat 1990) |  |  | 2 |
|  |  | *Mustura shanensis* (Hora 1929) |  |  |  |
|  | 25 | *Nemacheilus nandingensis* Zhu & Wang 1985 |  |  | 1 |
|  | 26 | *Neonoemacheilus labeosus* (Kottelat 1982) |  |  | 2 |
|  |  | *Neonoemacheilus mengdingensis* Zhu & Guo 1989 |  |  |  |
|  | 27 | *Paracanthocobitis botia* (Hamilton 1822) |  |  | 3 |
|  |  | *Paracanthocobitis rubidipinnis* (Blyth 1860) |  |  |  |
|  |  | *Paracanthocobitis zonalternans* (Blyth 1860) |  |  |  |
|  | 28 | *Petruichthys brevis* (Boulenger 1893) |  |  | 1 |
|  | 29 | *Pteronemacheilus lucidorsum* Bohlen & Šlechtová 2011 |  |  | 1 |
|  | 30 | *Physoschistura brunneana* (Annandale 1918) |  |  | 4 |
|  |  | *Physoschistura raoi* (Hora 1929) |  |  |  |
|  |  | *Physoschistura rivulicola* (Hora 1929) |  |  |  |
|  |  | *Physoschistura shuangjiangensis* (Zhu et Wang 1985) |  |  |  |
|  | 31 | *Schistura absumbra* (Endruweit 2017) |  |  | 17 |
|  |  | *Schistura alticrista* Kottelat 1990 |  |  |  |
|  |  | *Schistura cincticauda* (Blyth 1860) |  |  |  |
|  |  | *Schistura cryptofasciata* Chen, Kong & Yang 2005 |  |  |  |
|  |  | *Schistura disparizona* Zhou & Kottelat 2005 |  |  |  |
|  |  | *Schistura greenei* Endruweit 2017 |  |  |  |
|  |  | *Schistura kengtungensis* (Fowler 1936) |  |  |  |
|  |  | *Schistura longa* (Zhu 1982) |  |  |  |
|  |  | *Schistura mahnerti* Kottelat 1990 |  |  |  |
|  |  | *Schistura moeiensis* Kottelat 1990 |  |  |  |
|  |  | *Schistura nandingensis* Zhu & Wang 1985 |  |  |  |
|  |  | *Schistura paucicincta* Kottelat 1990 |  |  |  |
|  |  | *Schistura poculi* (Smith 1945) |  |  |  |
|  |  | *Schistura prolixifasciata* Zheng, Yang & Chen 2012 |  |  |  |
|  |  | *Schistura reidi* (Smith 1945) |  |  |  |
|  |  | *Schistura similis* Kottelat 1990 |  |  |  |
|  |  | *Schistura vinciguerrae* (Hora 1935) |  |  |  |
|  | 32 | *Triplophysa microps* (Steindachner 1866) |  |  | 4 |
|  |  | *Triplophysa nujiangensa* Chen, Cui & Yang 2004 |  |  |  |
|  |  | *Triplophysa stenura* (Herzenstein 1888) |  |  |  |
|  |  | *Triplophysa stoliczkae* (Steindachner 1866) |  |  |  |
| 12 |  | Psilorhynchidae | 1 | 1 |  |
|  | 33 | *Psilorhynchus robustus* Conway & Kottelat 2007 |  |  | 1 |
| 13 |  | Cyprinidae |  |  |  |
| A |  | Labeoninae | 10 | 22 |  |
|  | 34 | *Ageneiogarra nujiangensis* (Chen & Yang 2009) |  |  | 1 |
|  | 35 | *Akrokolioplax bicornis* (Wu 1977) |  |  | 1 |
|  | 36 | *Bangana almorae* (Chaudhuri 1912) |  |  | 2 |
|  |  | *Bangana devdevi* (Hora 1936) |  |  |  |
|  | 37 | *Cirrhinus cirrhosus* (Bloch 1795) |  |  | 1 |
|  | 38 | *Garra gotyla* (Gray 1830) |  |  | 7 |
|  |  | *Garra gravelyi* (Annandale 1919) |  |  |  |
|  |  | *Garra imberbis* (Vinciguerra 1890) |  |  |  |
|  |  | *Garra lamta* (Hamilton 1822) |  |  |  |
|  |  | *Garra nasuta* (McClelland 1838) |  |  |  |
|  |  | *Garra notata* (Blyth 1860) |  |  |  |
|  |  | *Garra salweenica* Hora & Mukerji 1934 |  |  |  |
|  | 39 | *Gymnostomus horai* (Bănărescu 1986) |  |  | 1 |
|  | 40 | *Labeo catla* (Hamilton 1822) |  |  | 6 |
|  |  | *Labeo curchius* (Hamilton 1822) |  |  |  |
|  |  | *Labeo dyocheilus* (McClelland 1839) |  |  |  |
|  |  | *Labeo fimbriatus* (Bloch 1795) |  |  |  |
|  |  | *Labeo pierrei* (Sauvage 1880) |  |  |  |
|  |  | *Labeo rohita* (Hamilton 1822) |  |  |  |
|  | 41 | *Labiobarbus leptocheilus* (Valenciennes 1842) |  |  | 1 |
|  | 42 | *Placocheilus cryptonemus* Cui & Li 1984 |  |  | 1 |
|  | 43 | *Tariqilabeo burmanicus* (Hora 1936) |  |  | 1 |
| B |  | Torinae | 3 | 17 |  |
|  | 44 | *Folifer brevifilis* (Peters 1881) |  |  | 1 |
|  | 45 | *Neolissochilus baoshanensis* (Chen & Yang 1999) |  |  | 11 |
|  |  | *Neolissochilus compressus* (Day 1870) |  |  |  |
|  |  | *Neolissochilus dukai* (Day 1878) |  |  |  |
|  |  | *Neolissochilus hemispinus* (Chen & Chu 1985) |  |  |  |
|  |  | *Neolissochilus hexagonolepis* (McClelland 1839) |  |  |  |
|  |  | *Neolissochilus hexastichus* (McClelland 1839) |  |  |  |
|  |  | *Neolissochilus nigrovittatus* (Boulenger 1893) |  |  |  |
|  |  | *Neolissochilus paucisquamatus* (Smith 1945) |  |  |  |
|  |  | *Neolissochilus stevensonii* (Day 1870) |  |  |  |
|  |  | *Neolissochilus stracheyi* (Day 1870) |  |  |  |
|  |  | *Neolissochilus vittatus* (Smith 1945) |  |  |  |
|  | 46 | *Tor mosal* (Hamilton 1822) |  |  | 5 |
|  |  | *Tor putitora* (Hamilton 1822) |  |  |  |
|  |  | *Tor tambra* (Valenciennes 1842) |  |  |  |
|  |  | *Tor tambroides* (Bleeker 1854) |  |  |  |
|  |  | *Tor tor* (Hamilton 1822) |  |  |  |
| C |  | Smiliogastrinae | 8 | 15 |  |
|  | 47 | *Barbodes binotatus* (Valenciennes 1842) |  |  | 1 |
|  | 48 | *Chagunius baileyi* Rainboth 1986 |  |  | 1 |
|  | 49 | *Hampala salweenensis* Doi & Taki 1994 |  |  | 1 |
|  | 50 | *Osteobrama alfrediana* (Valenciennes 1844) |  |  | 2 |
|  |  | *Osteobrama feae* Vinciguerra 1890 |  |  |  |
|  | 51 | *Osteochilus vittatus* (Valenciennes 1842) |  |  | 1 |
|  | 52 | *Pethia phutunio* (Hamilton 1822) |  |  | 2 |
|  |  | *Pethia stoliczkana* (Day 1871) |  |  |  |
|  | 53 | *Puntius burmanicus* (Day 1878) |  |  | 4 |
|  |  | *Puntius chola* (Hamilton 1822) |  |  |  |
|  |  | *Puntius puntio* (Hamilton 1822) |  |  |  |
|  |  | *Puntius sophore* (Hamilton 1822) |  |  |  |
|  | 54 | *Systomus compressiformis* (Cockerell 1913) |  |  | 3 |
|  |  | *Systomus orphoides* (Valenciennes 1842) |  |  |  |
|  |  | *Systomus sarana* (Hamilton 1822) |  |  |  |
| D |  | Cyprininae | 8 | 17 |  |
|  | 55 | *Barbonymus gonionotus* (Bleeker 1849) |  |  | 1 |
|  | 56 | *Carassius auratus* (Linnaeus 1758) |  |  | 1 |
|  | 57 | *Cyclocheilichthys apogon* (Valenciennes 1842) |  |  | 2 |
|  |  | *Cyclocheilichthys repasson* (Bleeker 1853) |  |  |  |
|  | 58 | *Cyprinus intha* Annandale 1918 |  |  | 1 |
|  | 59 | *Hypsibarbus oatesii* (Boulenger 1893) |  |  | 2 |
|  |  | *Hypsibarbus salweenensis* Rainboth 1996 |  |  |  |
|  | 60 | *Mystacoleucus argenteus* (Day 1888) |  |  | 2 |
|  |  | *Mystacoleucus obtusirostris* (Valenciennes 1842) |  |  |  |
|  | 61 | *Poropuntius carinatus* (Wu & Lin 1977) |  |  | 8 |
|  |  | *Poropuntius chondrorhynchus* (Fowler 1934) |  |  |  |
|  |  | *Poropuntius genyognathus* Roberts 1998 |  |  |  |
|  |  | *Poropuntius hampaloides* (Vinciguerra 1890) |  |  |  |
|  |  | *Poropuntius heterolepidotus* Roberts 1998 |  |  |  |
|  |  | *Poropuntius opisthopterus* (Wu 1977) |  |  |  |
|  |  | *Poropuntius schanicus* (Boulenger 1893) |  |  |  |
|  |  | *Poropuntius shanensis* (Hora & Mukerji 1934) |  |  |  |
|  | 62 | *Sawbwa resplendens* Annandale 1918 |  |  | 1 |
| E |  | Barbinae | 1 | 2 |  |
|  | 63 | *Scaphiodonichthys acanthopterus* (Fowler 1934) |  |  | 2 |
|  |  | *Scaphiodonichthys burmanicus* Vinciguerra 1890 |  |  |  |
| F |  | Schizothoracinae | 2 | 5 |  |
|  | 64 | *Percocypris retrodorslis* Cui & Chu 1990 |  |  | 1 |
|  | 65 | *Schizothorax gongshanensis* Tsao 1964 |  |  | 4 |
|  |  | *Schizothorax lissolabiatus* Tsao 1964 |  |  |  |
|  |  | *Schizothorax nukiangensis* Tsao 1964 |  |  |  |
|  |  | *Schizothorax paoshanensis* Tsao 1964 |  |  |  |
| G |  | Schizopygopsinae | 2 | 2 |  |
|  | 66 | *Gymnodiptychus integrigymnatus* Mo 1989 |  |  | 1 |
|  | 67 | *Schizopygopsis thermalis* Herzenstein 1891 |  |  | 1 |
| 14 |  | Danionidae |  |  |  |
| A |  | Chedrinae | 4 | 10 |  |
|  | 68 | *Cabdio morar* (Hamilton 1822) |  |  | 1 |
|  | 69 | *Opsarius barna* (Hamilton 1822) |  |  | 5 |
|  |  | *Opsarius barnoides* (Vinciguerra 1890) |  |  |  |
|  |  | *Opsarius bendelisis* (Hamilton 1807) |  |  |  |
|  |  | *Opsarius caudiocellatus* (Chu 1984) |  |  |  |
|  |  | *Opsarius ornatus* (Sauvage 1883) |  |  |  |
|  | 70 | *Raiamas bola* (Hamilton 1822) |  |  | 2 |
|  |  | *Raiamas guttatus* (Day 1870) |  |  |  |
|  | 71 | *Salmostoma sardinella* (Valenciennes 1844) |  |  | 2 |
|  |  | *Salmostoma sladoni* (Day 1870) |  |  |  |
| B |  | Rasborinae | 2 | 4 |  |
|  | 72 | *Amblypharyngodon atkinsonii* (Blyth 1860) |  |  | 2 |
|  |  | *Amblypharyngodon mola* (Hamilton 1822) |  |  |  |
|  | 73 | *Rasbora daniconius* (Hamilton 1822) |  |  | 2 |
|  |  | *Rasbora hobelmani* Kottelat 1984 |  |  |  |
| C |  | Danioninae | 7 | 20 |  |
|  | 74 | *Danio albolineatus* (Blyth 1860) |  |  | 6 |
|  |  | *Danio dangila* (Hamilton 1822) |  |  |  |
|  |  | *Danio erythromicron* (Annandale 1918) |  |  |  |
|  |  | *Danio margaritatus* (Roberts 2007) |  |  |  |
|  |  | *Danio nigrofasciatus* (Day 1870) |  |  |  |
|  |  | *Danio rerio* (Hamilton 1822) |  |  |  |
|  | 75 | *Danionella translucida* Roberts 1986 |  |  | 1 |
|  | 76 | *Devario aequipinnatus* (McClelland 1839) |  |  | 8 |
|  |  | *Devario ahlanderi* Kullander & Norén 2022 |  |  |  |
|  |  | *Devario annandalei* (Chaudhuri 1908) |  |  |  |
|  |  | *Devario browni* (Regan 1907) |  |  |  |
|  |  | *Devario kakhienensis* (Anderson 1879) |  |  |  |
|  |  | *Devario shanensis* (Hora 1928) |  |  |  |
|  |  | *Devario sondhii* (Hora & Mukerji 1934) |  |  |  |
|  |  | *Devario spinosus* (Day 1870) |  |  |  |
|  | 77 | *Inlecypris auropurpureus* (Annandale 1918) |  |  | 2 |
|  |  | *Inlecypris jayarami* (Barman 1985) |  |  |  |
|  | 78 | *Laubuka laubuca* (Hamilton 1822) |  |  | 1 |
|  | 79 | *Microdevario nana* (Kottelat & Witte 1999) |  |  | 1 |
|  | 80 | *Microrasbora rubescens* Annandale 1918 |  |  | 1 |
| D |  | Esominae | 1 | 3 |  |
|  | 81 | *Esomus altus* (Blyth 1860) |  |  | 3 |
|  |  | *Esomus danrica* (Hamilton 1822) |  |  |  |
|  |  | *Esomus metallicus* Ahl 1924 |  |  |  |
|  |  | 7. SILURIFORMES |  |  |  |
| 15 |  | Chacidae | 1 | 1 |  |
|  | 82 | *Chaca burmensis* Brown & Ferraris 1988 |  |  | 1 |
| 16 |  | Plotosidae | 1 | 1 |  |
|  | 83 | *Plotosus canius* Hamilton 1822 |  |  | 1 |
| 17 |  | Ailiidae | 4 | 7 |  |
|  | 84 | *Clupisoma prateri* Hora 1937 |  |  | 2 |
|  |  | *Clupisoma yunnanensis* (He, Huang & Li 1995) |  |  |  |
|  | 85 | *Eutropiichthys burmannicus* Day 1877 |  |  | 3 |
|  |  | *Eutropiichthys salweenensis* Ferraris & Vari 2007 |  |  |  |
|  |  | *Eutropiichthys vacha* (Hamilton 1822) |  |  |  |
|  | 86 | *Proeutropiichthys macropthalmos* (Blyth 1860) |  |  | 1 |
|  | 87 | *Silonia silondia* (Hamilton 1822) |  |  | 1 |
| 18 |  | Horabagridae | 1 | 1 |  |
|  | 88 | *Pachypterus acutirostris* (Day 1870) |  |  | 1 |
| 19 |  | Bagridae | 6 | 13 |  |
|  | 89 | *Batasio affinis* Blyth 1860 |  |  | 3 |
|  |  | *Batasio dayi* (Vinciguerra 1890) |  |  |  |
|  |  | *Batasio feruminatus* Ng & Kottelat 2008 |  |  |  |
|  | 90 | *Hemibagrus imbrifer* Ng & Ferraris 2000 |  |  | 2 |
|  |  | *Hemibagrus microphthalmus* (Day 1877) |  |  |  |
|  | 91 | *Mystus cavasius* (Hamilton 1822) |  |  | 3 |
|  |  | *Mystus falcarius* Chakrabarty & Ng 2005 |  |  |  |
|  |  | *Mystus rufescens* (Vinciguerra 1890) |  |  |  |
|  | 92 | *Olyra horae* (Prashad & Mukerji 1929) |  |  | 2 |
|  |  | *Olyra longicaudata* McClelland 1842 |  |  |  |
|  | 93 | *Rita sacerdotum* Anderson 1879 |  |  | 1 |
|  | 94 | *Sperata acicularis* Ferraris & Runge 1999 |  |  | 2 |
|  |  | *Sperata aor* (Hamilton 1822) |  |  |  |
| 20 |  | Akysidae |  |  |  |
|  |  | Akysinae | 1 | 1 |  |
|  | 95 | *Akysis vespa* Ng & Kottelat 2004 |  |  | 1 |
| 21 |  | Amblycipitidae | 1 | 5 |  |
|  | 96 | *Amblyceps caecutiens* Blyth 1858 |  |  | 5 |
|  |  | *Amblyceps carinatum* Ng 2005 |  |  |  |
|  |  | *Amblyceps foratum* Ng & Kottelat 2000 |  |  |  |
|  |  | *Amblyceps mangois* (Hamilton 1822) |  |  |  |
|  |  | *Amblyceps platycephalus* Ng & Kottelat 2000 |  |  |  |
| 22 |  | Sisoridae |  |  |  |
|  |  | Sisorinae | 5 | 19 |  |
|  | 97 | *Bagarius bagarius* (Hamilton 1822) |  |  | 2 |
|  |  | *Bagarius yarrelli* (Sykes 1839) |  |  |  |
|  | 98 | *Caelatoglanis zonatus* Ng & Kottelat 2005 |  |  | 1 |
|  | 99 | *Erethistes filamentosus* (Blyth 1860) |  |  | 2 |
|  |  | *Erethistes pusillus* Müller & Troschel 1849 |  |  |  |
|  | 100 | *Gagata cenia* (Hamilton 1822) |  |  | 3 |
|  |  | *Gagata dolichonema* He 1996 |  |  |  |
|  |  | *Gagata melanopterus* Roberts & Ferraris 1998 |  |  |  |
|  | 101 | *Glyptothorax burmanicus* Prashad & Mukerji 1929 |  |  | 11 |
|  |  | *Glyptothorax dorsalis* Vinciguerra 1890 |  |  |  |
|  |  | *Glyptothorax fucatus* Jiang, Ng, Wang & Chen 2012 |  |  |  |
|  |  | *Glyptothorax granosus* Jiang, Ng, Yang & Chen 2012 |  |  |  |
|  |  | *Glyptothorax lanceatus* Ng, Jiang & Chen 2012 |  |  |  |
|  |  | *Glyptothorax longinema* Li 1984 |  |  |  |
|  |  | *Glyptothorax ngapang* Vishwanath & Linthoingambi 2007 |  |  |  |
|  |  | *Glyptothorax obliquimaculatus* Jiang, Chen & Yang 2010 |  |  |  |
|  |  | *Glyptothorax rugimentum* Ng & Kottelat 2008 |  |  |  |
|  |  | *Glyptothorax trilineatus* Blyth 1860 |  |  |  |
|  |  | *Glyptothorax zanaensis* Wu, He & Chu 1981 |  |  |  |
|  |  | Glyptosterninae | 6 | 16 |  |
|  | 102 | *Barbeuchiloglanis feae* (Vinciguerra 1890) |  |  | 1 |
|  | 103 | *Creteuchiloglanis gongshanensis* (Chu 1981) |  |  | 2 |
|  |  | *Creteuchiloglanis macropterus* (Ng 2004) |  |  |  |
|  | 104 | *Exostoma berdmorei* Blyth 1860 |  |  | 4 |
|  |  | *Exostoma gaoligongense* Chen, Poly, Catania & Jiang 2017 |  |  |  |
|  |  | *Exostoma labiatum* (McClelland 1842) |  |  |  |
|  |  | *Exostoma vinciguerrae* Regan 1905 |  |  |  |
|  | 105 | *Oreoglanis heteropogon* Vidthayanon, Saenjundaeng & Ng 2009 |  |  | 5 |
|  |  | *Oreoglanis immaculatus* Kong, Chen & Yang 2007 |  |  |  |
|  |  | *Oreoglanis insignis* Ng & Rainboth 2001 |  |  |  |
|  |  | *Oreoglanis laciniosus* Vidthayanon, Saenjundaeng & Ng 2009 |  |  |  |
|  |  | *Oreoglanis macroptera* (Vinciguerra 1890) |  |  |  |
|  | 106 | *Pseudecheneis longipectoralis* Zhou, Li & Yang 2008 |  |  | 3 |
|  |  | *Pseudecheneis paucipunctatus* Zhou, Li & Yang 2008 |  |  |  |
|  |  | *Pseudecheneis sulcata* (McClelland 1842) |  |  |  |
|  | 107 | *Pseudexostoma brachysoma* Chu 1979 |  |  | 2 |
|  |  | *Pseudexostoma longipterus* Zhou, Yang, Li & Li 2007 |  |  |  |
| 23 |  | Pangasiidae | 1 | 1 |  |
|  | 108 | *Pangasius myanmar* Roberts & Vidthayanon 1991 |  |  | 1 |
| 24 |  | Siluridae | 4 | 5 |  |
|  | 109 | *Ompok bimaculatus* (Bloch 1794) |  |  | 2 |
|  |  | *Ompok pabo* (Hamilton 1822) |  |  |  |
|  | 110 | *Pterocryptis berdmorei* (Blyth 1860) |  |  | 1 |
|  | 111 | *Silurus burmanensis* Thant 1966 |  |  | 1 |
|  | 112 | *Wallago attu* (Bloch & Schneider) |  |  | 1 |
| 25 |  | Clariidae | 1 | 3 |  |
|  | 113 | *Clarias batrachus* (Linnaeus 1877) |  |  | 3 |
|  |  | *Clarias fuscus* (Lacepède 1803) |  |  |  |
|  |  | *Clarias gariepinus* (Burchell 1822) |  |  |  |
| 26 |  | Heteropneustidae | 1 | 1 |  |
|  | 114 | *Heteropneustes fossilis* (Bloch 1794) |  |  | 1 |
| 27 |  | Ariidae |  |  |  |
|  |  | Ariinae | 2 | 2 |  |
|  | 115 | *Arius acutirostris* Day 1877 |  |  | 1 |
|  | 116 | *Cochlefelis burmanica* (Day 1870) |  |  | 1 |
|  |  | 8. KURTIFORMES |  |  |  |
| 28 |  | Kurtidae | 1 | 1 |  |
|  | 117 | *Kurtus indicus* Bloch 1786 |  |  | 1 |
|  |  | 9. GOBIIFORMES |  |  |  |
| 29 |  | Eleotridae |  |  |  |
|  |  | Butinae | 1 | 1 |  |
|  | 118 | *Odonteleotris macrodon* (Bleeker 1853) |  |  | 1 |
| 30 |  | Gobiidae |  |  |  |
| A |  | Gobionellinae | 4 | 4 |  |
|  | 119 | *Brachygobius nunus* (Hamilton 1822) |  |  | 1 |
|  | 120 | *Gobiopterus chuno* (Hamilton 1822) |  |  | 1 |
|  | 121 | *Mugilogobius rambaiae* (Smith 1945) |  |  | 1 |
|  | 122 | *Stigmatogobius sadanundio* (Hamilton 1822) |  |  | 1 |
| B |  | Oxudercinae | 3 | 3 |  |
|  | 123 | *Apocryptes bato* (Hamilton 1822) |  |  | 1 |
|  | 124 | *Boleophthalmus boddarti* (Pallas 1770) |  |  | 1 |
|  | 125 | *Periophthalmodon septemradiatus* (Hamilton 1822) |  |  | 1 |
| C |  | Amblyopinae | 1 | 1 |  |
|  | 126 | *Odontamblyopus tenuis* (Day 1876) |  |  | 1 |
| D |  | Gobiinae | 1 | 1 |  |
|  | 127 | *Glossogobius giuris* (Hamilton 1822) |  |  | 1 |
|  |  | 10. SYNBRANCHIFORMES |  |  |  |
| 31 |  | Mastacembelidae | 2 | 7 |  |
|  | 128 | *Macrognathus caudiocellatus* (Boulenger 1893) |  |  | 2 |
|  |  | *Macrognathus zebrinus* (Blyth 1858) |  |  |  |
|  | 129 | *Mastacembelus alboguttatus* Boulenger 1893 |  |  | 5 |
|  |  | *Mastacembelus armatus* (Lacepède 1800) |  |  |  |
|  |  | *Mastacembelus caudiocellatus* (Boulenger 1893) |  |  |  |
|  |  | *Mastacembelus oatesii* Boulenger 1893 |  |  |  |
|  |  | *Mastacembelus tinwini* Britz 2007 |  |  |  |
| 32 |  | Chaudhuriidae | 1 | 1 |  |
|  | 130 | *Chaudhuria caudata* Annandale 1918 |  |  | 1 |
| 33 |  | Synbranchidae | 3 | 4 |  |
|  | 131 | *Monopterus albus* (Zuiew 1793) |  |  | 2 |
|  |  | *Monopterus javanensis* Lacepède 1800 |  |  |  |
|  | 132 | *Ophichthys cuchia* (Hamilton 1822) |  |  | 1 |
|  | 133 | *Ophisternon bengalense* McClelland 1844 |  |  | 1 |
|  |  | 12. ANABANTIFORMES |  |  |  |
| 34 |  | Anabantidae | 1 | 1 |  |
|  | 134 | *Anabas testudineus* (Bloch 1792) |  |  | 1 |
| 35 |  | Osphronemidae |  |  |  |
| A |  | Luciocephalinae | 1 | 2 |  |
|  | 135 | *Parasphaerichthys lineatus* Britz & Kottelat 2002 |  |  | 2 |
|  |  | *Parasphaerichthys ocellatus* Prashad & Mukerji 1929 |  |  |  |
| B |  | Trichogastrinae | 2 | 5 |  |
|  | 136 | *Trichogaster fasciata* Bloch & Schneider 1801 |  |  | 3 |
|  |  | *Trichogaster labiosa* Day 1877 |  |  |  |
|  |  | *Trichogaster lalius* (Hamilton 1822) |  |  |  |
|  | 137 | *Trichopodus pectoralis* Regan 1910 |  |  | 2 |
|  |  | *Trichopodus trichopterus* (Pallas 1770) |  |  |  |
| C |  | Macropodusinae | 1 | 1 |  |
|  | 138 | *Pseudosphromenus cupanus* (Cuvier 1831) |  |  | 1 |
| 36 |  | Channidae | 1 | 7 |  |
|  | 139 | *Channa aurolineata* (Day 1870) |  |  | 7 |
|  |  | *Channa gachua* (Hamilton 1822) |  |  |  |
|  |  | *Channa harcourtbutleri* (Annandale 1918) |  |  |  |
|  |  | *Channa marulius* (Hamilton 1822) |  |  |  |
|  |  | *Channa punctata* (Bloch 1793) |  |  |  |
|  |  | *Channa shingon* Endruweit 2017 |  |  |  |
|  |  | *Channa striata* (Bloch 1793) |  |  |  |
| 37 |  | Nandidae | 1 | 1 |  |
|  | 140 | *Nandus nandus* (Hamilton 1822) |  |  | 1 |
| 38 |  | Badidae | 1 | 1 |  |
|  | 141 | *Badis ruber* Schreitmüller 1923 |  |  | 1 |
|  |  | 13. CARANGIFORMES |  |  |  |
| 39 |  | Latidae | 1 | 2 |  |
|  | 142 | *Lates calcarifer* (Bloch 1790) |  |  | 2 |
|  |  | *Lates uwisara* Pethiyagoda & Gill 2012 |  |  |  |
| 40 |  | Toxotidae | 1 | 3 |  |
|  | 143 | *Toxotes blythii* Boulenger 1892 |  |  | 3 |
|  |  | *Toxotes chatareus* (Hamilton 1822) |  |  |  |
|  |  | *Toxotes jaculatrix* (Pallas 1767) |  |  |  |
|  |  | 14. CICHLIFORMES |  |  |  |
| 41 |  | Ambassidae | 2 | 9 |  |
|  | 144 | *Chanda nama* Hamilton 1822 |  |  | 1 |
|  | 145 | *Parambassis alleni alleni* (Datta & Chaudhuri 1993) |  |  | 8 |
|  |  | *Parambassis baculis* (Hamilton 1822) |  |  |  |
|  |  | *Parambassis lala* (Hamilton 1822) |  |  |  |
|  |  | *Parambassis pulcinella* Kottelat 2003 |  |  |  |
|  |  | *Parambassis ranga* (Hamilton 1822) |  |  |  |
|  |  | *Parambassis robertsi* (Datta & Chaudhuri 1993) |  |  |  |
|  |  | *Parambassis tenasserimensis* Roberts 1995 |  |  |  |
|  |  | *Parambassis vollmeri* Roberts 1995 |  |  |  |
|  |  | 15. CYPRINODONTIFORMES |  |  |  |
| 42 |  | Aplocheilidae | 1 | 1 |  |
|  | 146 | *Aplocheilus panchax* (Hamilton 1822) |  |  | 1 |
|  |  | 16. BELONIFORMES |  |  |  |
| 43 |  | Belonidae | 1 | 1 |  |
|  | 147 | *Xenentodon cancila* (Hamilton 1822) |  |  | 1 |
| 44 |  | Hemiramphidae | 1 | 1 |  |
|  | 148 | *Hyporhamphus limbatus* (Valenciennes 1847) |  |  | 1 |
| 45 |  | Zenarchopteridae | 2 | 2 |  |
|  | 149 | *Dermogenys burmanica* Mukerji 1935 |  |  | 1 |
|  | 150 | *Zenarchopterus ectuntio* (Hamilton 1822) |  |  | 1 |
| 46 |  | Adrianichthyidae |  |  |  |
|  |  | Oryziinae | 1 | 4 |  |
|  | 151 | *Oryzias dancena* (Hamilton 1822) |  |  | 4 |
|  |  | *Oryzias minutillus* Smith 1945 |  |  |  |
|  |  | *Oryzias sinensis* Chen, Uwa & Chu 1989 |  |  |  |
|  |  | *Oryzias uwai* Roberts 1998 |  |  |  |
|  |  | 17. MUGILIFORMES |  |  |  |
| 47 |  | Mugilidae | 6 | 8 |  |
|  | 152 | *Chelon melinopterus* (Valenciennes 1836) |  |  | 1 |
|  | 153 | *Crenimugil buchanani* (Bleeker 1853) |  |  | 2 |
|  |  | *Crenimugil seheli* (Forsskål 1775) |  |  |  |
|  | 154 | *Minimugil cascasia* (Hamilton 1822) |  |  | 1 |
|  | 155 | *Mugil cephalus* Linnaeus 1758 |  |  | 1 |
|  | 156 | *Planiliza macrolepis* (Smith 1846) |  |  | 2 |
|  |  | *Planiliza subviridis* (Valenciennes 1836) |  |  |  |
|  | 157 | *Rhinomugil corsula* (Hamilton 1822) |  |  | 1 |
|  |  | 18. CENTRARCHIFORMES |  |  |  |
| 48 |  | Terapontidae | 1 | 1 |  |
|  | 158 | *Terapon jarbua* (Forsskål 1775) |  |  | 1 |
|  |  | 19. ACANTHURIFORMES |  |  |  |
| 49 |  | Sillaginidae | 2 | 2 |  |
|  | 159 | *Sillaginopsis domina* (Cuvier 1816) |  |  | 1 |
|  | 160 | *Sillago sihama* (Forsskål 1775) |  |  | 1 |
| 50 |  | Lutjanidae |  |  |  |
|  |  | Lutjaninae | 1 | 1 |  |
|  | 161 | *Lutjanus argentimaculatus* (Forsskål 1775) |  |  | 1 |
| 51 |  | Sparidae | 1 | 1 |  |
|  | 162 | *Acanthopagrus berda* (Forsskål 1775) |  |  | 1 |
| 52 |  | Sciaenidae | 2 | 2 |  |
|  | 163 | *Johnius borneensis* (Bleeker 1851) |  |  | 1 |
|  | 164 | *Otolithoides pama* (Hamilton 1822) |  |  | 1 |
| 53 |  | Monodactylidae | 1 | 1 |  |
|  | 165 | *Monodactylus argenteus* (Linnaeus 1758) |  |  | 1 |
| 54 |  | Lobotidae | 1 | 1 |  |
|  | 166 | *Datnioides polota* (Hamilton 1822) |  |  | 1 |
| 55 |  | Scatophagidae | 1 | 1 |  |
|  | 167 | *Scatophagus argus* (Linnaeus 1766) |  |  | 1 |
|  |  | 20. TETRAODONTIFORMES |  |  |  |
| 56 |  | Tetraodontidae | 3 | 4 |  |
|  | 168 | *Chonerhinos naritus* (Richardson 1848) |  |  | 1 |
|  | 169 | *Dichotomyctere fluviatilis* (Hamilton 1822) |  |  | 2 |
|  |  | *Dichotomyctere nigroviridis* (Marion de Procé 1822) |  |  |  |
|  | 170 | *Leiodon cutcutia* (Hamilton 1822) |  |  | 1 |
|  |  | 合计Total | 170 | 362 | 362 |

Note:

1. For taxa (order, family, and subfamily), the name of order is in capital letters, the name of family and subfamily is in lowercase letters, and the family name is written to the left of the cell and the subfamily name is to the right.

2. The total number of families/subfamilies is 71.
