# Supplementary material for: Fish diversity and selection of taxa for conservation in the Salween and Irrawaddy Rivers, Southeast Asia
Source: Sci Rep. 2024 Jan 29;14:2393. doi: 10.1038/s41598-024-51205-5 (PMC10825156; doi:10.1038/s41598-024-51205-5)
Supplement: Supplementary file 2 — Supplementary Information 2. [file 41598_2024_51205_MOESM2_ESM.docx]

#### Supplementary Appendix 1-2 List of fish species found in the Irrawaddy River.

| Family No. | Genus No. | Taxa (order, family, subfamily, genus, and species) | Family/subfamily | | Species in the genus |
| --- | --- | --- | --- | --- | --- |
|  |  |  | Genera | Species |  |
|  |  | **Elasmobranchii** |  |  |  |
|  |  | 1. CARCHARHINIFORMES |  |  |  |
| 1 |  | Carcharhinidae | 2 | 3 |  |
|  | 1 | *Glyphis gangeticus* (Müller & Henle 1839) |  |  | 2 |
|  |  | *Glyphis glyphis* (Müller & Henle 1839) |  |  |  |
|  | 2 | *Scoliodon laticaudus* Müller & Henle 1838 |  |  | 1 |
|  |  | 2. RHINOPRISTIFORMES |  |  |  |
| 2 |  | Pristidae | 1 | 1 |  |
|  | 3 | *Pristis pristis* (Linnaeus 1758) |  |  | 1 |
|  |  | 3. MYLIOBATIFORMES |  |  |  |
| 3 |  | Dasyatidae |  |  |  |
| A |  | Hypolophinae | 1 | 1 |  |
|  | 4 | *Makararaja chindwinensis* Roberts 2007 |  |  | 1 |
|  |  | **Actinopteri** |  |  |  |
|  |  | 4. ELOPIFORMES |  |  |  |
| 4 |  | Megalopidae | 1 | 1 |  |
|  | 5 | *Megalops cyprinoides* (Broussonet 1782) |  |  | 1 |
|  |  | 5. ANGUILLIFORMES |  |  |  |
| 5 |  | Muraenidae |  |  |  |
|  |  | Muraeninae | 1 | 1 |  |
|  | 6 | *Gymnothorax tile* (Hamilton 1822) |  |  | 1 |
| 6 |  | Moringuidae | 1 | 1 |  |
|  | 7 | *Moringua raitaborua* (Hamilton 1822) |  |  | 1 |
| 7 |  | Anguillidae | 1 | 3 |  |
|  | 8 | *Anguilla bengalensis* (Gray 1831) |  |  | 3 |
|  |  | *Anguilla bicolor* McClelland 1844 |  |  |  |
|  |  | *Anguilla nebulosa* McClelland 1844 |  |  |  |
|  |  | 6. OSTEOGLOSSIFORMES |  |  |  |
| 8 |  | Notopteridae | 1 | 1 |  |
|  | 9 | *Notopterus notopterus* (Pallas 1769) |  |  | 1 |
|  |  | 7. CLUPEIFORMES |  |  |  |
| 9 |  | Engraulidae | 2 | 4 |  |
|  | 10 | *Coilia dussumieri* Valenciennes 1848 |  |  | 2 |
|  |  | *Coilia reynaldi* Valenciennes, 1848 |  |  |  |
|  | 11 | *Setipinna tenuifilis* (Valenciennes, 1848) |  |  | 2 |
|  |  | *Setipinna wheeler*i Wongratana, 1983 |  |  |  |
| 10 |  | Clupeidae | 4 | 5 |  |
|  | 12 | *Corica soborna* Hamilton, 1822 |  |  | 1 |
|  | 13 | *Gonialosa modesta* (Day, 1870) |  |  | 1 |
|  | 14 | *Gudusia chapra* (Hamilton, 1822) |  |  | 2 |
|  |  | *Gudusia variegata* (Day, 1870) |  |  |  |
|  | 15 | *Hilsa kelee* (Cuvier, 1829) |  |  | 1 |
| 11 |  | Pristigasteridae | 2 | 3 |  |
|  | 16 | *Ilisha megaloptera* (Swainson 1839) |  |  | 2 |
|  |  | *Ilisha novacula* (Valenciennes, 1847) |  |  |  |
|  | 17 | *Pellona ditchela* Valenciennes, 1847 |  |  | 1 |
| 12 |  | Dorosomatidae | 1 | 1 |  |
|  | 18 | *Tenualosa ilisha* (Hamilton, 1822) |  |  | 1 |
|  |  | 8. CYPRINIFORMES |  |  |  |
| 13 |  | Botiidae |  |  |  |
|  |  | Botiinae | 2 | 4 |  |
|  | 19 | *Botia histrionica* Blyth 1860 |  |  | 3 |
|  |  | *Botia kubotai* Kottelat 2004 |  |  |  |
|  |  | *Botia rostrata* Günther 1868 |  |  |  |
|  | 20 | *Syncrossus berdmorei* Blyth 1860 |  |  | 1 |
| 14 |  | Cobitidae | 4 | 16 |  |
|  | 21 | *Acantopsis spectabilis* (Blyth 1860) |  |  | 1 |
|  | 22 | *Lepidocephalichthys alkaia* Havird & Page 2010 |  |  | 9 |
|  |  | *Lepidocephalichthys berdmorei* (Blyth 1860) |  |  |  |
|  |  | *Lepidocephalichthys eleios* Kottelat 2017 |  |  |  |
|  |  | *Lepidocephalichthys goalparensis* Pillai & Yazdani 1976 |  |  |  |
|  |  | *Lepidocephalichthys guntea* (Hamilton 1822) |  |  |  |
|  |  | *Lepidocephalichthys hasselti* (Valenciennes 1846) |  |  |  |
|  |  | *Lepidocephalichthys* *irrorata* Hora 1921 |  |  |  |
|  |  | *Lepidocephalichthys longipinnis* (Menon 1992) |  |  |  |
|  |  | *Lepidocephalichthys micropogon* (Blyth 1860) |  |  |  |
|  | 23 | *Misgurnus anguillicaudatus* (Cantor 1842) |  |  | 1 |
|  | 24 | *Pangio elongata* Britz & Maclaine 2007 |  |  | 5 |
|  |  | *Pangio lumbriciformis* Britz & Maclaine 2007 |  |  |  |
|  |  | *Pangio oblonga* (Valenciennes 1846) |  |  |  |
|  |  | *Pangio pangia* (Hamilton 1822) |  |  |  |
|  |  | *Pangio signicauda* Britz & Maclaine 2007 |  |  |  |
| 15 |  | Balitoridae | 4 | 6 |  |
|  | 25 | *Aborichthys kempi* Chaudhuri 1913 |  |  | 1 |
|  | 26 | *Balitora brucei* Gray 1830 |  |  | 2 |
|  |  | *Balitora burmanica* Hora 1932 |  |  |  |
|  | 27 | *Hemimyzon yingjiangensis* Chen 2006 |  |  | 1 |
|  | 28 | *Homalopteroides manipurensis* (Arunkumar 1999) |  |  | 2 |
|  |  | *Homalopteroides rupicola* (Prashad & Mukerji 1929) |  |  |  |
| 16 |  | Nemacheilidae | 7 | 45 |  |
|  | 29 | *Malihkaia aligera* Kottelat 2017 |  |  | 1 |
|  | 30 | *Mustura celata* Kottelat 2018 |  |  | 8 |
|  |  | *Mustura chindwinensis* (Lokeshwor & Vishwanath 2012) |  |  |  |
|  |  | *Mustura prashadi* (Hora 1921) |  |  |  |
|  |  | *Mustura shuensis* (Bohlen & Šlechtová 2014) |  |  |  |
|  |  | *Mustura taretensis* Chinglemba, Rameshori & Vishwanath 2021 |  |  |  |
|  |  | *Mustura tigrina* (Lokeshwor & Vishwanath 2012) |  |  |  |
|  |  | *Mustura yangi* Qin, Kottelat, Kyaw & Chen 2022 |  |  |  |
|  |  | *Mustura yunnaniloides* (Chen, Kottelat & Neely 2011) |  |  |  |
|  | 31 | *Neonoemacheilus morehensis* Arunkumar 2000 |  |  | 2 |
|  |  | *Neonoemacheilus peguensis* (Hora 1929) |  |  |  |
|  | 32 | *Paracanthocobitis adelaideae* Singer & Page 2015 |  |  | 9 |
|  |  | *Paracanthocobitis botia* (Hamilton 1822) |  |  |  |
|  |  | *Paracanthocobitis linypha* Singer & Page 2015 |  |  |  |
|  |  | *Paracanthocobitis mandalayensis* (Rendahl 1948) |  |  |  |
|  |  | *Paracanthocobitis marmorata* Singer, Pfeiffer & Page 2017 |  |  |  |
|  |  | *Paracanthocobitis nigrolineata* Singer, Pfeiffer & Page 2017 |  |  |  |
|  |  | *Paracanthocobitis putaoensis* Lin, Chen & Chen 2023 |  |  |  |
|  |  | *Paracanthocobitis rubidipinnis* (Blyth 1860) |  |  |  |
|  |  | *Paracanthocobitis zonalternans* (Blyth 1860) |  |  |  |
|  | 33 | *Protonemacheilus longipectoralis* Yang & Chu 1990 |  |  | 1 |
|  | 34 | *Pteronemacheilus lucidorsum* Bohlen & Šlechtová 2011 |  |  | 1 |
|  | 35 | *Schistura acuticephala* (Hora 1929) |  |  | 23 |
|  |  | *Schistura albirostris* Chen & Neely 2012 |  |  |  |
|  |  | *Schistura altuscauda* Chen, Myint, Chu & Chen 2020 |  |  |  |
|  |  | *Schistura callidora* Bohlen & Šlechtová 2011 |  |  |  |
|  |  | *Schistura indawgyiana* Kottelat 2017 |  |  |  |
|  |  | *Schistura kangjupkhulensis* (Hora 1921) |  |  |  |
|  |  | *Schistura khugae* Vishwanath & Shanta 2004 |  |  |  |
|  |  | *Schistura malaisei* Kottelat 1990 |  |  |  |
|  |  | *Schistura manipurensis* (Chaudhuri 1912) |  |  |  |
|  |  | *Schistura megalodon* Endruweit 2014 |  |  |  |
|  |  | *Schistura nagaensis* (Menon 1987) |  |  |  |
|  |  | *Schistura nubigena* Kottelat 2017 |  |  |  |
|  |  | *Schistura paucifasciata* (Hora 1929) |  |  |  |
|  |  | *Schistura pawensis* Bohlen & Šlechtová 2013 |  |  |  |
|  |  | *Schistura phamhringi* Shangningam, Lokeshwor & Vishwanath 2014 |  |  |  |
|  |  | *Schistura polytaenia* (Zhu 1982) |  |  |  |
|  |  | *Schistura puncticeps* Bohlen & Šlechtová 2013. |  |  |  |
|  |  | *Schistura reticulata* Vishwanath & Nebeshwar Sharma 2004 |  |  |  |
|  |  | *Schistura rubrimaculata* Bohlen & Šlechtová 2013 |  |  |  |
|  |  | *Schistura sikmaiensis* (Hora 1921) |  |  |  |
|  |  | *Schistura vinciguerrae* (Hora 1935) |  |  |  |
|  |  | *Schistura wanlainensis* Kottelat 2017 |  |  |  |
|  |  | *Schistura yingjiangensis* (Zhu 1982) |  |  |  |
| 17 |  | Psilorhynchidae | 1 | 14 |  |
|  | 36 | *Psilorhynchus balitora* (Hamilton 1822) |  |  | 14 |
|  |  | *Psilorhynchus brachyrhynchus* Conway & Britz 2010 |  |  |  |
|  |  | *Psilorhynchus breviminor* Conway & Mayden 2008 |  |  |  |
|  |  | *Psilorhynchus chakpiensis* Shangningam & Vishwanath 2013. |  |  |  |
|  |  | *Psilorhynchus gokkyi* Conway & Britz 2010 |  |  |  |
|  |  | *Psilorhynchus homaloptera* Hora & Mukerji 1935 |  |  |  |
|  |  | *Psilorhynchus konemi* Shangningam & Vishwanath 2016 |  |  |  |
|  |  | *Psilorhynchus maculatus* Shangningam & Vishwanath 2013 |  |  |  |
|  |  | *Psilorhynchus magnaoculus* Shangningam & Kosygin 2021 |  |  |  |
|  |  | *Psilorhynchus microphthalmus* Vishwanath & Manojkumar 1995 |  |  |  |
|  |  | *Psilorhynchus ngathanu* Shangningam & Vishwanath 2013 |  |  |  |
|  |  | *Psilorhynchus olliei* Conway & Britz 2015 |  |  |  |
|  |  | *Psilorhynchus piperatus* Conway & Britz 2010 |  |  |  |
|  |  | *Psilorhynchus rowleyi* Hora & Misra 1941 |  |  |  |
| 18 |  | Cyprinidae |  |  |  |
| A |  | Labeoninae | 5 | 41 |  |
|  | 37 | *Bangana devdevi* (Hora 1936) |  |  | 1 |
|  | 38 | *Cirrhinus cirrhosus* (Bloch 1795) |  |  | 1 |
|  | 39 | *Garra abhoyai* Hora 1921 |  |  | 25 |
|  |  | *Garra bispinosa* Zhang 2005 |  |  |  |
|  |  | *Garra chakpiensis* Nebeshwar & Vishwanath 2015 |  |  |  |
|  |  | *Garra compressus* Kosygin & Vishwanath 1998 |  |  |  |
|  |  | *Garra cornigera* Shangningam & Vishwanath 2015 |  |  |  |
|  |  | *Garra dulongensis* (Chen, Pan, Kong & Yang 2006) |  |  |  |
|  |  | *Garra elongata* Vishwanath & Kosygin 2000 |  |  |  |
|  |  | *Garra flavatra* Kullander & Fang 2004 |  |  |  |
|  |  | *Garra gotyla* (Gray 1830) |  |  |  |
|  |  | *Garra gravelyi* (Annandale 1919) |  |  |  |
|  |  | *Garra lamta* (Hamilton 1822) |  |  |  |
|  |  | *Garra litanensis* Vishwanath 1993 |  |  |  |
|  |  | *Garra manipurensis* Vishwanath & Sarjnalini 1988 |  |  |  |
|  |  | *Garra nambulica* Vishwanath & Joyshree 2005 |  |  |  |
|  |  | *Garra* *namyaensis* Shangningam & Vishwanath 2012 |  |  |  |
|  |  | *Garra nasuta* (McClelland 1838) |  |  |  |
|  |  | *Garra paralissorhynchus* Vishwanath & Shanta Devi 2005 |  |  |  |
|  |  | *Garra* *poecilura* Kullander & Fang 2004 |  |  |  |
|  |  | *Garra* *qiaojiensis* Wu & Yao 1977 |  |  |  |
|  |  | *Garra rotundinasus* Zhang 2006 |  |  |  |
|  |  | *Garra salweenica* Hora & Mukerji 1934 |  |  |  |
|  |  | *Garra* *spilota* Kullander & Fang 2004 |  |  |  |
|  |  | *Garra tengchongensis* Zhang & Chen 2002 |  |  |  |
|  |  | *Garra trilobata* Shangningam & Vishwanath 2015 |  |  |  |
|  |  | *Garra ukhrulensis* Nebeshwar & Vishwanath 2015 |  |  |  |
|  | 40 | *Labeo angra* (Hamilton 1822) |  |  | 13 |
|  |  | *Labeo bata* (Hamilton 1822) |  |  |  |
|  |  | *Labeo boga* (Hamilton 1822) |  |  |  |
|  |  | *Labeo calbasu* (Hamilton 1822) |  |  |  |
|  |  | *Labeo catla* (Hamilton 1822) |  |  |  |
|  |  | *Labeo curchius* (Hamilton 1822) |  |  |  |
|  |  | *Labeo dyocheilus* (McClelland 1839) |  |  |  |
|  |  | *Labeo gonius* (Hamilton 1822) |  |  |  |
|  |  | *Labeo inornatus* (Roberts 1997) |  |  |  |
|  |  | *Labeo nandina* (Hamilton 1822) |  |  |  |
|  |  | *Labeo pierrei* (Sauvage 1880) |  |  |  |
|  |  | *Labeo rohita* (Hamilton 1822) |  |  |  |
|  |  | *Labeo stolizkae* Steindachner 1870 |  |  |  |
|  | 41 | *Tariqilabeo burmanicus* (Hora 1936) |  |  | 1 |
| B |  | Torinae | 2 | 7 |  |
|  | 42 | *Neolissochilus baoshanensis* (Chen & Yang 1999) |  |  | 5 |
|  |  | *Neolissochilus compressus* (Day 1870) |  |  |  |
|  |  | *Neolissochilus heterostomus* (Chen & Yang 1999) |  |  |  |
|  |  | *Neolissochilus hexagonolepis* (McClelland 1839) |  |  |  |
|  |  | *Neolissochilus qiaojiensis* (Wu 1977) |  |  |  |
|  | 43 | *Tor putitora* (Hamilton 1822) |  |  | 2 |
|  |  | *Tor yingjiangensis* Chen & Yang 2004 |  |  |  |
| C |  | Smiliogastrinae | 7 | 32 |  |
|  | 44 | *Chagunius nicholsi* (Myers 1924) |  |  | 1 |
|  | 45 | *Oreichthys cosuatis* (Hamilton 1822) |  |  | 1 |
|  | 46 | *Osteobrama belangeri* (Valenciennes 1844) |  |  | 3 |
|  |  | *Osteobrama cunma* (Day 1888) |  |  |  |
|  |  | *Osteobrama feae* Vinciguerra 1890 |  |  |  |
|  | 47 | *Osteochilus vittatus* (Valenciennes 1842) |  |  | 1 |
|  | 48 | *Pethia atra* (Linthoingambi & Vishwanath 2007) |  |  | 19 |
|  |  | *Pethia castor* Conway, Pinion & Kottelat 2021 |  |  |  |
|  |  | *Pethia conchonius* (Hamilton 1822) |  |  |  |
|  |  | *Pethia didi* (Kullander & Fang 2005) |  |  |  |
|  |  | *Pethia erythromycter* (Kullander 2008) |  |  |  |
|  |  | *Pethia khugae* (Linthoingambi & Vishwanath 2007) |  |  |  |
|  |  | *Pethia macrogramma* (Kullander 2008) |  |  |  |
|  |  | *Pethia manipurensis* (Menon, Rema Devi & Vishwanath 2000) |  |  |  |
|  |  | *Pethia meingangbii* (Arunkumar & Tombi Singh 2003) |  |  |  |
|  |  | *Pethia nankyweensis* (Kullander 2008) |  |  |  |
|  |  | *Pethia ornata* (Vishwanath & Laisram 2004) |  |  |  |
|  |  | *Pethia padamya* (Kullander & Britz 2008) |  |  |  |
|  |  | *Pethia poiensis* Shangningam & Vishwanath 2018 |  |  |  |
|  |  | *Pethia pollux* Conway, Pinion & Kottelat 2021 |  |  |  |
|  |  | *Pethia stoliczkana* (Day 1870) |  |  |  |
|  |  | *Pethia thelys* (Kullander 2008) |  |  |  |
|  |  | *Pethia tiantian* (Kullander & Fang 2005) |  |  |  |
|  |  | *Pethia ticto* (Hamilton 1822) |  |  |  |
|  |  | *Pethia yuensis* (Arunkumar & Tombi Singh 2003) |  |  |  |
|  | 49 | *Puntius chola* (Hamilton 1822) |  |  | 4 |
|  |  | *Puntius pugio* Kullander 2008 |  |  |  |
|  |  | *Puntius puntio* (Hamilton 1822) |  |  |  |
|  |  | *Puntius sophore* (Hamilton 1822) |  |  |  |
|  | 50 | *Systomus binduchitra* (Hora 1937) |  |  | 3 |
|  |  | *Systomus sarana* (Hamilton 1822) |  |  |  |
|  |  | *Systomus sewelli* (Prashad & Mukerji 1929) |  |  |  |
| D |  | Cyprininae | 4 | 8 |  |
|  | 51 | *Carassius auratus* (Linnaeus 1758) |  |  | 1 |
|  | 52 | *Cyclocheilichthys apogon* (Valenciennes 1842) |  |  | 1 |
|  | 53 | *Hypsibarbus myitkyinae* (Prashad & Mukerji 1929) |  |  | 2 |
|  |  | *Hypsibarbus oatesii* (Boulenger 1893) |  |  |  |
|  | 54 | *Poropuntius bantamensis* (Rendahl 1920) |  |  | 4 |
|  |  | *Poropuntius burtoni* (Mukerji 1933) |  |  |  |
|  |  | *Poropuntius margarianus* (Anderson 1879) |  |  |  |
|  |  | *Poropuntius shanensis* (Hora & Mukerji 1934) |  |  |  |
| E |  | Barbinae | 3 | 4 |  |
|  | 55 | *Cyprinion semiplotum* (McClelland 1839) |  |  | 1 |
|  | 56 | *Scaphiodonichthys burmanicus* Vinciguerra 1890 |  |  | 1 |
|  | 57 | *Semiplotus cirrhosus* Chaudhuri 1919 |  |  | 2 |
|  |  | *Semiplotus manipurensis* Vishwanath & Kosygin 2000 |  |  |  |
| F |  | Schizothoracinae | 1 | 14 |  |
|  | 58 | *Schizothorax chivae* Arunkumar & Moyon 2016 |  |  | 14 |
|  |  | *Schizothorax dulongensis* Huang 1985 |  |  |  |
|  |  | *Schizothorax elongatus* Huang 1985 |  |  |  |
|  |  | *Schizothorax griseus* Pellegrin 1931 |  |  |  |
|  |  | *Schizothorax heteri* Yang, Zhen, Chen & Yang 2013 |  |  |  |
|  |  | *Schizothorax leukus* Yang, Zhen, Chen & Yang 2013 |  |  |  |
|  |  | *Schizothorax lissolabiatus* Tsao 1964 |  |  |  |
|  |  | *Schizothorax malacanthus* Huang 1985 |  |  |  |
|  |  | *Schizothorax meridionalis* Tsao 1964 |  |  |  |
|  |  | *Schizothorax myzostomus* Tsao 1964 |  |  |  |
|  |  | *Schizothorax nukiangensis* Tsao 1964 |  |  |  |
|  |  | *Schizothorax oligolepis* Huang 1985 |  |  |  |
|  |  | *Schizothorax rotundimaxillaris* Wu & Wu 1992 |  |  |  |
|  |  | *Schizothorax yunnanensis* Norman 1923 |  |  |  |
| I |  | Schizopygopsinae | 1 | 1 |  |
|  | 59 | *Gymnodiptychus integrigymnatus* Mo 1989 |  |  | 1 |
| 19 |  | Danionidae |  |  |  |
| A |  | Chedrinae | 6 | 16 |  |
|  | 60 | *Barilius barila* (Hamilton 1822) |  |  | 1 |
|  | 61 | *Bengala elanga* (Hamilton 1822) |  |  | 1 |
|  | 62 | *Cabdio morar* (Hamilton 1822) |  |  | 2 |
|  |  | *Cabdio ukhrulensis* (Selim & Vishwanath 2001) |  |  |  |
|  | 63 | *Opsarius barna* (Hamilton 1822) |  |  | 8 |
|  |  | *Opsarius barnoides* (Vinciguerra 1890) |  |  |  |
|  |  | *Opsarius bendelisis* (Hamilton 1807) |  |  |  |
|  |  | *Opsarius chatricensis* (Selim & Vishwanath 2002) |  |  |  |
|  |  | *Opsarius dogarsinghi* (Hora 1921) |  |  |  |
|  |  | *Opsarius lairokensis* (Arunkumar & Tombi Singh 2000) |  |  |  |
|  |  | *Opsarius ngawa* (Vishwanath & Manojkumar 2002) |  |  |  |
|  |  | *Opsarius putaoensis* Qin, Maung & Chen 2019 |  |  |  |
|  | 64 | *Raiamas guttatus* (Day 1870) |  |  | 1 |
|  | 65 | *Salmostoma balookee* (Sykes 1839) |  |  | 3 |
|  |  | *Salmostoma sardinella* (Valenciennes 1844) |  |  |  |
|  |  | *Salmostoma sladoni* (Day 1870) |  |  |  |
| B |  | Rasborinae | 2 | 5 |  |
|  | 66 | *Amblypharyngodon atkinsonii* (Blyth 1860) |  |  | 2 |
|  |  | *Amblypharyngodon mola* (Hamilton 1822) |  |  |  |
|  | 67 | *Rasbora daniconius* (Hamilton 1822) |  |  | 3 |
|  |  | *Rasbora ornata* Vishwanath & Laisram 2005 |  |  |  |
|  |  | *Rasbora rasbora* (Hamilton 1822) |  |  |  |
| C |  | Danioninae | 5 | 35 |  |
|  | 68 | *Danio albolineatus* (Blyth 1860) |  |  | 11 |
|  |  | *Danio choprae* Hora 1928 |  |  |  |
|  |  | *Danio dangila* (Hamilton 1822) |  |  |  |
|  |  | *Danio flagrans* Kullander 2012 |  |  |  |
|  |  | *Danio htamanthinus* Kullander & Norén 2016 |  |  |  |
|  |  | *Danio kyathit* Fang 1998 |  |  |  |
|  |  | *Danio nigrofasciatus* (Day 1870) |  |  |  |
|  |  | *Danio quagga* Kullander, Liao & Fang 2009 |  |  |  |
|  |  | *Danio rerio* (Hamilton 1822) |  |  |  |
|  |  | *Danio tinwini* Kullander & Fang 2009 |  |  |  |
|  |  | *Danio tweediei* Brittan 1956 |  |  |  |
|  | 69 | *Danionella cerebrum* Britz, Conway & Rüber 2021 |  |  | 4 |
|  |  | *Danionella dracula* Britz, Conway & Rüber 2009 |  |  |  |
|  |  | *Danionella mirifica* Britz 2003 |  |  |  |
|  |  | *Danionella translucida* Roberts 1986 |  |  |  |
|  | 70 | *Devario acuticephala* (Hora 1921) |  |  | 15 |
|  |  | *Devario aequipinnatus* (McClelland 1839) |  |  |  |
|  |  | *Devario apogon* (Chu 1981) |  |  |  |
|  |  | *Devario browni* (Regan 1907) |  |  |  |
|  |  | *Devario deruptotalea* Ramananda & Vishwanath 2014 |  |  |  |
|  |  | *Devario fangae* Kullander 2017 |  |  |  |
|  |  | *Devario interruptus* (Day 1870) |  |  |  |
|  |  | *Devario kakhienensis* (Anderson 1879) |  |  |  |
|  |  | *Devario manipurensis* (Barman 1987) |  |  |  |
|  |  | *Devario myitkyinae* Kullander 2017 |  |  |  |
|  |  | *Devario naganensis* (Chaudhuri 1912) |  |  |  |
|  |  | *Devario shanensis* (Hora 1928) |  |  |  |
|  |  | *Devario spinosus* (Day 1870) |  |  |  |
|  |  | *Devario strigillifer* (Myers 1924) |  |  |  |
|  |  | *Devario yuensis* (Arunkumar & Tombi Singh 1998) |  |  |  |
|  | 71 | *Laubuka laubuca* (Hamilton 1822) |  |  | 2 |
|  |  | *Laubuka tenella* Kullander, Rahman, Norén & Mollah 2018 |  |  |  |
|  | 72 | *Microdevario gatesi* (Herre 1939) |  |  | 3 |
|  |  | *Microdevario microphthalma* (Jiang, Chen & Yang 2008) |  |  |  |
|  |  | *Microdevario nanus* (Kottelat & Witte 1999) |  |  |  |
| D |  | Esominae | 1 | 5 |  |
|  | 73 | *Esomus ahli* Hora & Mukerji 1928 |  |  | 5 |
|  |  | *Esomus altus* (Blyth 1860) |  |  |  |
|  |  | *Esomus caudiocellatus* Ahl 1923 |  |  |  |
|  |  | *Esomus danrica* (Hamilton 1822) |  |  |  |
|  |  | *Esomus longimanus* (Lunel 1881) |  |  |  |
|  |  | 9. SILURIFORMES |  |  |  |
| 20 |  | Chacidae | 1 | 1 |  |
|  | 74 | *Chaca burmensis* Brown & Ferraris 1988 |  |  | 1 |
| 21 |  | Plotosidae | 1 | 1 |  |
|  | 75 | *Plotosus canius* Hamilton 1822 |  |  | 1 |
| 22 |  | Ailiidae | 4 | 6 |  |
|  | 76 | *Clupisoma prateri* Hora 1937 |  |  | 2 |
|  |  | *Clupisoma roosae* Ferraris 2004 |  |  |  |
|  | 77 | *Eutropiichthys britzi* Ferraris & Vari 2007 |  |  | 2 |
|  |  | *Eutropiichthys burmannicus* Day 1877 |  |  |  |
|  | 78 | *Proeutropiichthys macropthalmos* (Blyth 1860) |  |  | 1 |
|  | 79 | *Silonia silondia* (Hamilton 1822) |  |  | 1 |
| 23 |  | Horabagridae | 1 | 1 |  |
|  | 80 | *Pachypterus acutirostris* (Day 1870) |  |  | 1 |
| 24 |  | Bagridae | 6 | 20 |  |
|  | 81 | *Batasio affinis* Blyth 1860 |  |  | 4 |
|  |  | *Batasio dayi* (Vinciguerra 1890) |  |  |  |
|  |  | *Batasio procerus* Ng 2008 |  |  |  |
|  |  | *Batasio tengana* (Hamilton 1822) |  |  |  |
|  | 82 | *Hemibagrus microphthalmus* (Day 1877) |  |  | 2 |
|  |  | *Hemibagrus peguensis* (Boulenger 1894) |  |  |  |
|  | 83 | *Mystus bleekeri* (Day 1877) |  |  | 9 |
|  |  | *Mystus cavasius* (Hamilton 1822) |  |  |  |
|  |  | *Mystus cineraceus* Ng & Kottelat 2009 |  |  |  |
|  |  | *Mystus falcarius* Chakrabarty & Ng 2005 |  |  |  |
|  |  | *Mystus gulio* (Hamilton 1822) |  |  |  |
|  |  | *Mystus leucophasis* (Blyth 1860) |  |  |  |
|  |  | *Mystus ngasep* Darshan, Vishwanath, Mahanta & Barat 2011 |  |  |  |
|  |  | *Mystus pulcher* (Chaudhuri 1911) |  |  |  |
|  |  | *Mystus rufescens* (Vinciguerra 1890) |  |  |  |
|  | 84 | *Olyra burmanica* Day 1872 |  |  | 2 |
|  |  | *Olyra horae* (Prashad & Mukerji 1929) |  |  |  |
|  | 85 | *Rita sacerdotum* Anderson 1879 |  |  | 1 |
|  | 86 | *Sperata acicularis* Ferraris & Runge 1999 |  |  | 2 |
|  |  | *Sperata aor* (Hamilton 1822) |  |  |  |
| 25 |  | Akysidae |  |  |  |
|  |  | Akysinae | 1 | 2 |  |
|  | 87 | *Akysis manipurensis* (Arunkumar 2000) |  |  | 2 |
|  |  | *Akysis prashadi* Hora 1936 |  |  |  |
| 26 |  | Amblycipitidae | 1 | 6 |  |
|  | 88 | *Amblyceps carinatum* Ng 2005 |  |  | 6 |
|  |  | *Amblyceps improcerum* Ng & Kottelat 2018 |  |  |  |
|  |  | *Amblyceps murraystuarti* Chaudhuri 1919 |  |  |  |
|  |  | *Amblyceps torrentis* Linthoingambi & Vishwanath 2008 |  |  |  |
|  |  | *Amblyceps tuberculatum* Linthoingambi & Vishwanath 2008 |  |  |  |
|  |  | *Amblyceps yunnanense* Zhang, Long, Xiao & Chen 2016 |  |  |  |
| 27 |  | Sisoridae |  |  |  |
| A |  | Sisorinae | 7 | 26 |  |
|  | 89 | *Ayarnangra estuarius* Roberts 2001 |  |  | 1 |
|  | 90 | *Bagarius bagarius* (Hamilton 1822) |  |  | 2 |
|  |  | *Bagarius yarrelli* (Sykes 1839) |  |  |  |
|  | 91 | *Erethistes longissimus* (Ng & Kottelat 2007) |  |  | 1 |
|  | 92 | *Erethistoides longispinis* Ng, Ferraris & Neely 2012 |  |  | 2 |
|  |  | *Erethistoides vesculus* Ng, Ferraris & Neely 2012 |  |  |  |
|  | 93 | *Gagata dolichonema* He 1996 |  |  | 3 |
|  |  | *Gagata gagata* (Hamilton 1822) |  |  |  |
|  |  | *Gagata melanopterus* Roberts & Ferraris 1998 |  |  |  |
|  | 94 | *Glyptothorax burmanicus* Prashad & Mukerji 1929 |  |  | 15 |
|  |  | *Glyptothorax chavomensis* Arunkumar & Moyon 2017 |  |  |  |
|  |  | *Glyptothorax dorsalis* Vinciguerra 1890 |  |  |  |
|  |  | *Glyptothorax granulus* Vishwanath & Linthoingambi 2007 |  |  |  |
|  |  | *Glyptothorax igniculus* Ng & Kullander 2013 |  |  |  |
|  |  | *Glyptothorax longicauda* Li 1984 |  |  |  |
|  |  | *Glyptothorax longjiangensis* Mo & Chu 1986 |  |  |  |
|  |  | *Glyptothorax manipurensis* Menon 1955 |  |  |  |
|  |  | *Glyptothorax minimaculatus* Li 1984 |  |  |  |
|  |  | *Glyptothorax ngapang* Vishwanath & Linthoingambi 2007 |  |  |  |
|  |  | *Glyptothorax panda* Ferraris & Britz 2005 |  |  |  |
|  |  | *Glyptothorax senapatiensis* Premananda, Kosygin & Saidullah 2015 |  |  |  |
|  |  | *Glyptothorax trilineatus* Blyth 1860 |  |  |  |
|  |  | *Glyptothorax ventrolineatus* Vishwanath & Linthoingambi 2006 |  |  |  |
|  |  | *Glyptothorax yuensis* Shangningam & Kosygin 2022 |  |  |  |
|  | 95 | *Pseudolaguvia tenebricosa* Britz & Ferraris 2003 |  |  | 2 |
|  |  | *Pseudolaguvia tuberculata* (Prashad & Mukerji 1929) |  |  |  |
| B |  | Glyptosterninae | 7 | 21 |  |
|  | 96 | *Creteuchiloglanis brachypterus* Zhou, Li & Thomson 2011 |  |  | 2 |
|  |  | *Creteuchiloglanis macropterus* (Ng 2004) |  |  |  |
|  | 97 | *Exostoma chaudhurii* (Hora 1923) |  |  | 7 |
|  |  | *Exostoma dujangense* Shangningam & Kosygin 2020 |  |  |  |
|  |  | *Exostoma dulongensis* Luo et Chen 2020 |  |  |  |
|  |  | *Exostoma ericinum* Ng 2018 |  |  |  |
|  |  | *Exostoma sectile* Ng & Kottelat 2018 |  |  |  |
|  |  | *Exostoma stuarti* (Hora 1923) |  |  |  |
|  |  | *Exostoma vinciguerrae* Regan 1905 |  |  |  |
|  | 98 | *Glaridoglanis andersonii* (Day 1870) |  |  | 2 |
|  |  | *Glaridoglanis ramosa* Ng & Kottelat 2022 |  |  |  |
|  | 99 | *Myersglanis jayarami* Vishwanath & Kosygin 1999 |  |  | 1 |
|  | 100 | *Oreoglanis hponkanensis* Chen, Qin & Chen 2017 |  |  | 3 |
|  |  | *Oreoglanis insignis* Ng & Rainboth 2001 |  |  |  |
|  |  | *Oreoglanis macroptera* (Vinciguerra 1890) |  |  |  |
|  | 101 | *Pseudecheneis brachyura* Zhou, Li & Yang 2008 |  |  | 5 |
|  |  | *Pseudecheneis gracilis* Zhou, Li & Yang 2008 |  |  |  |
|  |  | *Pseudecheneis stenura* Ng 2006 |  |  |  |
|  |  | *Pseudecheneis sulcata* (McClelland 1842) |  |  |  |
|  |  | *Pseudecheneis ukhrulensis* Vishwanath & Darshan 2007 |  |  |  |
|  | 102 | *Pseudexostoma yunnanense* (Tchang 1935) |  |  | 1 |
| 28 |  | Pangasiidae | 1 | 2 |  |
|  | 103 | *Pangasius myanmar* Roberts & Vidthayanon 1991 |  |  | 2 |
|  |  | *Pangasius pangasius* (Hamilton 1822) |  |  |  |
| 29 |  | Siluridae | 3 | 5 |  |
|  | 104 | *Ompok bimaculatus* (Bloch 1794) |  |  | 3 |
|  |  | *Ompok pabda* (Hamilton 1822) |  |  |  |
|  |  | *Ompok pabo* (Hamilton 1822) |  |  |  |
|  | 105 | *Pterocryptis berdmorei* (Blyth 1860) |  |  | 1 |
|  | 106 | *Wallago attu* (Bloch & Schneider 1801) |  |  | 1 |
| 30 |  | Clariidae | 1 | 1 |  |
|  | 107 | *Clarias fuscus* (Lacepède 1803) |  |  | 1 |
| 31 |  | Heteropneustidae | 1 | 1 |  |
|  | 108 | *Heteropneustes fossilis* (Bloch 1794) |  |  | 1 |
| 32 |  | Ariidae |  |  |  |
|  |  | Ariinae | 5 | 7 |  |
|  | 109 | *Arius arius* (Hamilton 1822) |  |  | 3 |
|  |  | *Arius nenga* (Hamilton 1822) |  |  |  |
|  |  | *Arius venosus* Valenciennes 1840 |  |  |  |
|  | 110 | *Cephalocassis jatius* (Hamilton 1822) |  |  | 1 |
|  | 111 | *Cochlefelis burmanicus* (Day 1870) |  |  | 1 |
|  | 112 | *Netuma bilineata* (Valenciennes 1840) |  |  | 1 |
|  | 113 | *Osteogeneiosus militaris* (Linnaeus 1758) |  |  | 1 |
|  |  | 10. SYNGNATHIFORMES |  |  |  |
| 33 |  | Syngnathidae |  |  |  |
|  |  | Nerophinae | 1 | 1 |  |
|  | 114 | *Microphis dunckeri* (Prashad & Mukerji 1929) |  |  | 1 |
|  |  | 11. KURTIFORMES |  |  |  |
| 34 |  | Kurtidae | 1 | 1 |  |
|  | 115 | *Kurtus indicus* Bloch 1786 |  |  | 1 |
|  |  | 12. GOBIIFORMES |  |  |  |
| 35 |  | Eleotridae |  |  |  |
|  |  | Butinae | 3 | 3 |  |
|  | 116 | *Butis gymnopomus* (Bleeker 1853) |  |  | 1 |
|  | 117 | *Eleotris melanosoma* Bleeker 1853 |  |  | 1 |
|  | 118 | *Odonteleotris macrodon* (Bleeker 1853) |  |  | 1 |
| 36 |  | Gobiidae |  |  |  |
| A |  | Gobionellinae | 7 | 8 |  |
|  | 119 | *Awaous grammepomus* (Bleeker 1849) |  |  | 1 |
|  | 120 | *Brachygobius nunus* (Hamilton 1822) |  |  | 1 |
|  | 121 | *Gobiopterus chuno* (Hamilton 1822) |  |  | 1 |
|  | 122 | *Mugilogobius rambaiae* (Smith 1945) |  |  | 1 |
|  | 123 | *Oligolepis acutipennis* (Valenciennes 1837) |  |  | 1 |
|  | 124 | *Redigobius balteatus* (Herre 1935) |  |  | 2 |
|  |  | *Redigobius bikolanus* (Herre 1927) |  |  |  |
|  | 125 | *Stigmatogobius sadanundio* (Hamilton 1822) |  |  | 1 |
| B |  | Oxudercinae | 4 | 4 |  |
|  | 126 | *Apocryptes bato* (Hamilton 1822) |  |  | 1 |
|  | 127 | *Boleophthalmus boddarti* (Pallas 1770) |  |  | 1 |
|  | 128 | *Periophthalmodon septemradiatus* (Hamilton 1822) |  |  | 1 |
|  | 129 | *Pseudapocryptes elongatus* (Cuvier 1816) |  |  | 1 |
| C |  | Amblyopinae | 4 | 6 |  |
|  | 130 | *Brachyamblyopus burmanicus* Hora 1926 |  |  | 1 |
|  | 131 | *Caragobius urolepis* (Bleeker 1852) |  |  | 1 |
|  | 132 | *Odontamblyopus rubicundus* (Hamilton 1822) |  |  | 2 |
|  |  | *Odontamblyopus tenuis* (Day 1876) |  |  |  |
|  | 133 | *Taenioides anguillaris* (Linnaeus 1758) |  |  | 2 |
|  |  | *Taenioides buchanani* (Day 1873) |  |  |  |
| D |  | Gobiinae | 7 | 7 |  |
|  | 134 | *Amblyeleotris gymnocephala* (Bleeker 1853) |  |  | 1 |
|  | 135 | *Bathygobius fuscus* (Rüppell 1830) |  |  | 1 |
|  | 136 | *Drombus globiceps* (Hora 1923) |  |  | 1 |
|  | 137 | *Favonigobius reichei* (Bleeker 1854) |  |  | 1 |
|  | 138 | *Glossogobius giuris* (Hamilton 1822) |  |  | 1 |
|  | 139 | *Istigobius ornatus* (Rüppell 1830) |  |  | 1 |
|  | 140 | *Yongeichthys nebulosus* (Forsskål 1775) |  |  | 1 |
|  |  | 13. SYNBRANCHIFORMES |  |  |  |
| 37 |  | Mastacembelidae | 2 | 11 |  |
|  | 141 | *Macrognathus aral* (Bloch & Schneider 1801) |  |  | 6 |
|  |  | *Macrognathus aureus* Britz 2010 |  |  |  |
|  |  | *Macrognathus dorsiocellatus* Britz 2010 |  |  |  |
|  |  | *Macrognathus morehensis* Arunkumar & Tombi Singh 2000 |  |  |  |
|  |  | *Macrognathus obscurus* Britz 2010 |  |  |  |
|  |  | *Macrognathus zebrinus* (Blyth 1858) |  |  |  |
|  | 142 | *Mastacembelus alboguttatus* Boulenger 1893 |  |  | 5 |
|  |  | *Mastacembelus armatus* (Lacepède 1800) |  |  |  |
|  |  | *Mastacembelus pantherinus* Britz 2007 |  |  |  |
|  |  | *Mastacembelus strigiventus* Zhou & Yang 2011 |  |  |  |
|  |  | *Mastacembelus triolobus* Zhou & Yang 2011 |  |  |  |
| 38 |  | Chaudhuriidae | 3 | 4 |  |
|  | 143 | *Chaudhuria caudata* Annandale 1918 |  |  | 2 |
|  |  | *Chaudhuria ritvae* Britz 2010 |  |  |  |
|  | 144 | *Pillaia kachinica* Kullander, Britz & Fang 2000 |  |  | 1 |
|  | 145 | *Pillaiabrachia siniae* Britz 2016 |  |  | 1 |
| 39 |  | Indostomidae | 1 | 1 |  |
|  | 146 | *Indostomus paradoxus* Prashad & Mukerji 1929 |  |  | 1 |
| 40 |  | Synbranchidae | 3 | 3 |  |
|  | 147 | *Monopterus albus* (Zuiew 1793) |  |  | 1 |
|  | 148 | *Ophichthys cuchia* (Hamilton 1822) |  |  | 1 |
|  | 149 | *Ophisternon bengalense* McClelland 1844 |  |  | 1 |
|  |  | 14. ANABANTIFORMES |  |  |  |
| 41 |  | Anabantidae | 1 | 1 |  |
|  | 150 | *Anabas testudineus* (Bloch 1792) |  |  | 1 |
| 42 |  | Osphronemidae |  |  |  |
| A |  | Luciocephalinae | 1 | 2 |  |
|  | 151 | *Parasphaerichthys lineatus* Britz & Kottelat 2002 |  |  | 2 |
|  |  | *Parasphaerichthys ocellatus* Prashad & Mukerji 1929 |  |  |  |
| B |  | Trichogastrinae | 2 | 3 |  |
|  | 152 | *Trichogaster fasciata* Bloch & Schneider 1801 |  |  | 2 |
|  |  | *Trichogaster labiosa* Day 1877 |  |  |  |
|  | 153 | *Trichopodus pectoralis* Regan 1910 |  |  | 1 |
| 43 |  | Channidae | 1 | 10 |  |
|  | 154 | *Channa aurolineata* (Day 1870) |  |  | 10 |
|  |  | *Channa barca* (Hamilton 1822) |  |  |  |
|  |  | *Channa burmanica* Chaudhuri 1919 |  |  |  |
|  |  | *Channa gachua* (Hamilton 1822) |  |  |  |
|  |  | *Channa marulius* (Hamilton 1822) |  |  |  |
|  |  | *Channa ornatipinnis* Britz 2008 |  |  |  |
|  |  | *Channa panaw* Musikasinthorn 1998 |  |  |  |
|  |  | *Channa punctata* (Bloch 1793) |  |  |  |
|  |  | *Channa shingon* Endruweit 2017 |  |  |  |
|  |  | *Channa striata* (Bloch 1793) |  |  |  |
| 44 |  | Nandidae | 1 | 1 |  |
|  | 155 | *Nandus nandus* (Hamilton 1822) |  |  | 1 |
| 45 |  | Badidae | 2 | 9 |  |
|  | 156 | *Badis corycaeus* Kullander & Britz 2002 |  |  | 5 |
|  |  | *Badis ferrarisi* Kullander & Britz 2002 |  |  |  |
|  |  | *Badis kyar* Kullander & Britz 2002 |  |  |  |
|  |  | *Badis pyema* Kullander & Britz 2002 |  |  |  |
|  |  | *Badis ruber* Schreitmüller 1923 |  |  |  |
|  | 157 | *Dario dayingensis* Kullander & Britz 2002 |  |  | 4 |
|  |  | *Dario hysginon* Kullander & Britz 2002 |  |  |  |
|  |  | *Dario melanogrammus* Britz, Kullander & Rüber 2022 |  |  |  |
|  |  | *Dario tigris* Britz, Kullander & Rüber 2022 |  |  |  |
|  |  | 15. CARANGIFORMES |  |  |  |
| 46 |  | Latidae | 1 | 2 |  |
|  | 158 | *Lates calcarifer* (Bloch 1790) |  |  | 2 |
|  |  | *Lates uwisara* Pethiyagoda & Gill 2012 |  |  |  |
| 47 |  | Polynemidae | 1 | 1 |  |
|  | 159 | *Polynemus paradiseus* Linnaeus 1758 |  |  | 1 |
| 48 |  | Soleidae | 2 | 2 |  |
|  | 160 | *Brachirus orientalis* (Bloch & Schneider 1801) |  |  | 1 |
|  | 161 | *Dagetichthys albomaculatus* (Kaup 1858) |  |  | 1 |
| 49 |  | Toxotidae | 1 | 2 |  |
|  | 162 | *Toxotes chatareus* (Hamilton 1822) |  |  | 2 |
|  |  | *Toxotes jaculatrix* (Pallas 1767) |  |  |  |
|  |  | 16. CICHLIFORMES |  |  |  |
| 50 |  | Ambassidae | 2 | 7 |  |
|  | 163 | *Chanda nama* Hamilton 1822 |  |  | 1 |
|  | 164 | *Parambassis alleni* (Datta & Chaudhuri 1993) |  |  | 6 |
|  |  | *Parambassis baculis* (Hamilton 1822) |  |  |  |
|  |  | *Parambassis lala* (Hamilton 1822) |  |  |  |
|  |  | *Parambassis ranga* (Hamilton 1822) |  |  |  |
|  |  | *Parambassis robertsi* (Datta & Chaudhuri 1993) |  |  |  |
|  |  | *Parambassis waikhomi* Geetakumari & Basudha 2012 |  |  |  |
|  |  | 17. CYPRINODONTIFORMES |  |  |  |
| 51 |  | Aplocheilidae | 1 | 1 |  |
|  | 165 | *Aplocheilus panchax* (Hamilton 1822) |  |  | 1 |
|  |  | 18. BELONIFORMES |  |  |  |
| 52 |  | Belonidae | 1 | 1 |  |
|  | 166 | *Xenentodon cancila* (Hamilton 1822) |  |  | 1 |
| 53 |  | Hemiramphidae | 1 | 1 |  |
|  | 167 | *Hyporhamphus limbatus* (Valenciennes 1847) |  |  | 1 |
| 54 |  | Zenarchopteridae | 2 | 2 |  |
|  | 168 | *Dermogenys burmanica* Mukerji 1935 |  |  | 1 |
|  | 169 | *Zenarchopterus ectuntio* (Hamilton 1822) |  |  | 1 |
| 55 |  | Adrianichthyidae |  |  |  |
|  |  | Oryziinae | 1 | 3 |  |
|  | 170 | *Oryzias dancena* (Hamilton 1822) |  |  | 3 |
|  |  | *Oryzias sinensis* Chen, Uwa & Chu 1989 |  |  |  |
|  |  | *Oryzias uwai* Roberts 1998 |  |  |  |
|  |  | 19. MUGILIFORMES |  |  |  |
| 56 |  | Mugilidae | 7 | 9 |  |
|  | 171 | *Chelon melinopterus* (Valenciennes 1836) |  |  | 1 |
|  | 172 | *Crenimugil buchanani* (Bleeker 1853) |  |  | 2 |
|  |  | *Crenimugil seheli* (Forsskål 1775) |  |  |  |
|  | 173 | *Mugil cephalus* Linnaeus 1758 |  |  | 1 |
|  | 174 | *Osteomugil speigleri* (Bleeker 1858) |  |  | 1 |
|  | 175 | *Planiliza macrolepis* (Smith 1846) |  |  | 2 |
|  |  | *Planiliza subviridis* (Valenciennes 1836) |  |  |  |
|  | 176 | *Rhinomugil corsula* (Hamilton 1822) |  |  | 1 |
|  | 177 | *Sicamugil hamiltonii* (Day 1870) |  |  | 1 |
|  |  | 20. CENTRARCHIFORMES |  |  |  |
| 57 |  | Terapontidae | 1 | 1 |  |
|  | 178 | *Terapon jarbua* (Forsskål 1775) |  |  | 1 |
|  |  | 21. ACANTHURIFORMES |  |  |  |
| 58 |  | Sillaginidae | 2 | 2 |  |
|  | 179 | *Sillaginopsis domina* (Cuvier 1816) |  |  | 1 |
|  | 180 | *Sillago sihama* (Forsskål 1775) |  |  | 1 |
| 59 |  | Lutjanidae |  |  |  |
|  |  | Lutjaninae | 1 | 1 |  |
|  | 181 | *Lutjanus argentimaculatus* (Forsskål 1775) |  |  | 1 |
| 60 |  | Gerreidae | 1 | 1 |  |
|  | 182 | *Gerres filamentosus* Cuvier 1829 |  |  | 1 |
| 61 |  | Sparidae | 1 | 1 |  |
|  | 183 | *Acanthopagrus berda* (Forsskål 1775) |  |  | 1 |
| 62 |  | Sciaenidae | 4 | 5 |  |
|  | 184 | *Bahaba chaptis* (Hamilton 1822) |  |  | 1 |
|  | 185 | *Johnius borneensis* (Bleeker 1851) |  |  | 2 |
|  |  | *Johnius coitor* (Hamilton 1822) |  |  |  |
|  | 186 | *Otolithoides pama* (Hamilton 1822) |  |  | 1 |
|  | 187 | *Pterotolithus maculatus* (Cuvier 1830) |  |  | 1 |
| 63 |  | Monodactylidae | 1 | 1 |  |
|  | 188 | *Monodactylus argenteus* (Linnaeus 1758) |  |  | 1 |
| 64 |  | Lobotidae | 1 | 1 |  |
|  | 189 | *Datnioides polota* (Hamilton 1822) |  |  | 1 |
| 65 |  | Scatophagidae | 1 | 1 |  |
|  | 190 | *Scatophagus argus* (Linnaeus 1766) |  |  | 1 |
|  |  | 22. TETRAODONTIFORMES |  |  |  |
| 66 |  | Tetraodontidae | 3 | 4 |  |
|  | 191 | *Chonerhinos naritus* (Richardson 1848) |  |  | 1 |
|  | 192 | *Dichotomyctere fluviatilis* (Hamilton 1822) |  |  | 2 |
|  |  | *Dichotomyctere nigroviridis* (Marionde Procé 1822) |  |  |  |
|  | 193 | *Leiodon cutcutia* (Hamilton 1822) |  |  | 1 |
|  |  | 合计Total | 193 | 502 | 502 |

Note:

1. For taxa (order, family, and subfamily), the name of order is written in capital letters, the name of family and subfamily is written in lowercase letters, and the family name is to the left of the cell and the subfamily name is to the right.

2. The total number of families/subfamilies is 80.
