# Supplementary material for: Fish diversity and selection of taxa for conservation in the Salween and Irrawaddy Rivers, Southeast Asia
Source: Sci Rep. 2024 Jan 29;14:2393. doi: 10.1038/s41598-024-51205-5 (PMC10825156; doi:10.1038/s41598-024-51205-5)
Supplement: Supplementary file 3 — Supplementary Information 3. [file 41598_2024_51205_MOESM3_ESM.docx]

### **SUPPLEMENTARY REFERENCES**

1. Arunkumar, L. 1999. *Homaloptera manipurensis*, a new homalopterid fish from Manipur, India. Uttar Pradesh Journal of Zoology, 19(3), 201–205.
2. Arunkumar, L., 2000a. *Neonoemacheilus morehensis*, a new species of Nemacheiline loach (Balitoridae: Nemacheilinae) from the Yu-River system of Manipur. Indian Journal of Fisheries, 47(1), 43–47.
3. Arunkumar, L., 2000b. *Laguvia manipurensis*, a new species of sisorid cat fish (Pisces: Sisoridae) from the Yu River system of Manipur. Indian Journal of Fisheries, 47(3), 193–200.
4. Arunkumar, L., Moyon, W.A., 2016. *Schizothorax chivae*, a new schizothoracid fish from Chindwin basin, Manipur, India (Teleostei: Cyprinidae). International Journal of Fauna and Biological Studies, 3(2)Part B, 65–70.
5. Arunkumar, L., Moyon, W.A., 2017. *Glyptothorax chavomensis* sp. nov. (Teleostei: Sisoridae) with its congeners from Manipur, North-Eastern India. International Journal of Zoology Studies, 2(5), 242–254.
6. Arunkumar, L., Moyon, W.A., 2019. *Paracanthocobitis tumitensis*, a new species of zipper loach from Manipur, north-eastern India (Cypriniformes: Nemacheilidae). Species, 20(64), 101–109.
7. Arunkumar, L., Singh, H.T., 1998. Fishes of the genus *Danio* (Hamilton-Buchanan) from Manipur, with description of a new species. Journal of Nature Conservation, 10(1), 1–6.
8. Arunkumar, L., Singh, H.T., 2000a. Spiny eels of the genus *Macrognathus* Lacepède from Manipur, with description of a new species. Journal of the Bombay Natural History Society, 97(1), 117–122.
9. Arunkumar, L., Singh, H.T., 2000b. Bariliine fishes of Manipur, India, with description of a new species: *Barilius lairokensis*. Journal of the Bombay Natural History Society, 97(2), 247–252.
10. Arunkumar, L., Singh, H.T., 2003. Two new species of puntiid fish from the Yu River system of Manipur. Journal of the Bombay Natural History Society, 99(3), 481–487.
11. Bhowmik, S., Pal, S., Das, D., Chakraborty, K. 2016. Ichthyofaunal diversity at lower part of sub Himalayan terai region of west Bengal. Proceedings- “Biodiversity”- Prospect and Threats: Present Scenario https://www.researchgate.net/publication/308725722.
12. Bohlen, J., Šlechtová, V., 2011. A new genus and two new species of loaches (Teleostei: Nemacheilidae) from Myanmar. Ichthyological Exploration of Freshwaters, 22(1), 1–10.
13. Bohlen, J., Šlechtová, V., 2013. *Schistura puncticeps*, a new species of loach from Myanmar (Cypriniformes: Nemacheilidae). Ichthyological Exploration of Freshwaters, 24(1), 85–92.
14. Bohlen, J., Šlechtová, V., 2013. Two new species of *Schistura* from Myanmar (Teleostei: Nemacheilidae). Ichthyological Exploration of Freshwaters, 24(1), 21–30.
15. Bohlen, J., Šlechtová, V., 2014. *Schistura shuensis*, a new species of loach from Myanmar (Teleostei: Nemacheilidae). Ichthyological Exploration of Freshwaters, 24(3), 217–223.
16. Britz, R., 2003. *Danionella mirifica*, a new species of miniature fish from Upper Myanmar (Ostariophysi: Cyprinidae). Ichthyological Exploration of Freshwaters, 14(3), 217–222.
17. Britz, R., 2007. Two new species of *Mastacembelus* from Myanmar (Teleostei: Synbranchiformes: Mastacembelidae). Ichthyological Exploration of Freshwaters, 18(3), 257–268.
18. Britz, R., 2008. *Channa ornatipinnis* and *C. pulchra*, two new species of dwarf snakeheads from Myanmar (Teleostei: Channidae). Ichthyological Exploration of Freshwaters, 18(4), 335–344.
19. Britz, R., 2010. A new earthworm eel of the genus *Chaudhuria* from the Ayeyarwaddy River drainage, Myanmar (Teleostei: Synbranchiformes: Chaudhuriidae). Zootaxa, 2571, 62–68.
20. Britz, R., 2010. *Macrognathus aureus*, a new spiny eel of the *M. aculeatus* species group from the Upper Ayeyarwaddy River drainage, Myanmar (Teleostei: Synbranchiformes: Mastacembelidae). Zootaxa, 2514, 55–60.
21. Britz, R., 2010. Species of the *Macrognathus aculeatus* group in Myanmar with remarks on *M. caudiocellatus* (Teleostei: Synbranchiformes: Mastacembelidae). Ichthyological Exploration of Freshwaters, 20(4), 295–308.
22. Britz, R., 2016. *Pillaiabrachia siniae*, a new species of earthworm eel from northern Myanmar (Teleostei: Synbranchiformes: Chaudhuriidae). Ichthyological Exploration of Freshwaters, 27(1), 41–47.
23. Britz, R., Conway, K.W., Rüber, L., 2009. Spectacular morphological novelty in a miniature cyprinid fish, *Danionella dracula* n. sp. Proceedings of the Royal Society of London, Series B - Biological Sciences. http://doi 10.1098/rspb.2009.0141.
24. Britz, R., Conway, K.W., Rüber, L., 2021. The emerging vertebrate model species for neurophysiological studies is *Danionella cerebrum*, new species (Teleostei: Cyprinidae). Scientific Reports, 11: 18942: 1–11.
25. Britz, R., Ferraris, C.J., 2003. A new species of the Asian catfish genus *Pseudolaguvia* from Myanmar (Teleostei: Ostariophysi: Siluriformes: Erethistidae). Zootaxa, 388, 1–8.
26. Britz, R., Kottelat, M., 2002. *Parasphaerichthys lineatus*, a new species of labyrinth fish from southern Myanmar (Teleostei: Osphronemidae). Ichthyological Exploration of Freshwaters, 13(3), 243–250.
27. Britz, R., Maclaine, J.S., 2007. A review of the eel-loaches, genus *Pangio*, from Myanmar (Teleostei: Cypriniformes: Cobitidae). Ichthyological Exploration of Freshwaters, 18(1), 17–30.
28. Brown, B.A., Ferraris, C.J., 1988. Comparative osteology of the Asian catfish family Chacidae, with the description of a new species from Burma. American Museum Novitates, 2907, 1–16.
29. Chakrabarty, P., Ng, H.H., 2005. The identity of catfishes identified as *Mystus cavasius* (Hamilton, 1822) (Teleostei: Bagridae), with a description of a new species from Myanmar. Zootaxa, 1093, 1–24.
30. Chen, M.F., Myint, K.M., Chu, L., Chen, X.Y., 2020. Two new species of loaches from the Irrawaddy River basin, Chin State, Myanmar (Teleostei: Cypriniformes: Nemacheilidae). Zootaxa, 4895(1), 86–102.
31. Chen, X.Y., Neely D.A., 2012. *Schistura albirostris*, a new nemacheiline loach (Teleostei: Balitoridae) from the Irrawaddy River drainage of Yunnan Province, China. Zootaxa, 3586, 222–227.
32. Chen, X.Y., Ferraris, C.J., Yang, J.X., 2005. A new species of catfish of the genus *Clupisoma* (Siluriformes: Schilbidae) from the Salween River, Yunnan, China. Copeia, 2005(3), 566–570.
33. Chen, X.Y., Kong, D.P., Yang J.X., 2005. *Schistura cryptofasciata*, a new loach (Cypriniformes: Balitoridae) from Salween drainage in Yunnan, southwestern China. Raffles Bulletin of Zoology, Suppl. 13, 27–32.
34. Chen, X.Y., Cui, G.H., Yang, J.X., 2004. A new fish species of genus *Triplophysa* (Balitoridae) from Nu Jiang, Yunnan, China. Zoological Research, 25(6), 504–509. (In Chinese, English abstract.)
35. Chen, X.Y., Cui, G.H., Yang, J.X., 2005. *Balitora nantingensis* (Teleostei: Balitoridae), a new hillstream loach from Salween drainage in Yunnan, southwestern China. Raffles Bulletin of Zoology, Suppl. 13, 21–26.
36. Chen, X.Y., Yang, J.X., Chen, Y.R., 1999. A review of the cyprinoid fish genus *Barbodes* Bleeker, 1859, from Yunnan, China, with descriptions of two new species. Zoological Studies, 38(1), 82–88.
37. Chen, X.Y., Kottelat, M., Neely, D.A., 2011. *Physoschistura yunnaniloides*, a new species of loach from Myanmar (Teleostei: Nemacheilidae). Ichthyological Exploration of Freshwaters, 22(2), 179–183.
38. Chen, X.Y., Qin, T., Chen, Z.Y., 2017. *Oreoglanis hponkanensis*, a new sisorid catfish from north Myanmar (Actinopterygii, Sisoridae). ZooKeys, 646, 95–108.
39. Chen, X.Y., Poly, W.J., Catania, D., Jiang, W.S., 2017. A new species of sisorid catfish of the genus *Exostoma* from the Salween drainage, Yunnan, China. Zoological Research, 38(5), 1–11.
40. Chen, Z.-M., Yang, J.X., 2004. A new species of the genus *Tor* from Yunnan, China (Teleostei: Cyprinidae). Environmental Biology of Fishes, 70(2), 185–191.
41. Chen, Z.M., Zhao, S., Yang, J.X., 2009. A new species of the genus *Garra* from Nujiang River Basin, Yunnnan, China (Telesotei: Cyprinidae). Zoological Research, 30(4), 438–444.
42. Chen, Z.M., Pan, X.F., Xiao, H., Yang, J.X., 2012. A new cyprinid species, *Placocheilus dulongensis*, from the upper Irrawaddy system in northwestern Yunnan, China. Zoologischer Anzeiger, 251(3), 215–222.
43. Chinglemba, Y., Rameshori, Y., Vishwanath, W., 2021. A new species of stone loach of the genus *Mustura* (Teleostei: Nemacheilidae) from Chindwin River drainage, Manipur, north-eastern India. Zootaxa, 5081(4), 551–565.
44. Conway, K.W., Britz, R., 2010. Three new species of *Psilorhynchus* from the Ayeyarwaddy River drainage, Myanmar (Teleostei: Psilorhynchidae). Zootaxa, 2616, 31–47.
45. Conway, K.W., Britz, R., 2015. *Psilorhynchus olliei*, a new species of torrent minnow from eastern Myanmar (Ostariophysi: Psilorhynchidae). Ichthyological Exploration of Freshwaters, 25(4), 347–356.
46. Conway, K.W., Kottelat, M., 2007. A new species of *Psilorhynchus* (Teleostei: Psilorhynchidae) from the Ataran River basin, Myanmar, with comments on the generic name *Psilorhynchoides*. Zootaxa, 1663, 47–57.
47. Conway, K.W., Mayden, R.L. 2008. *Psilorhynchus breviminor*, a new species of psilorhynchid fish from Myanmar (Ostariophysi: Psilorhynchidae). Ichthyological Exploration of Freshwaters, 19(2), 111–120.
48. Conway, K.W., Pinion, A.K., Kottelat, M., 2021. Two new species of *Pethia* (Teleostei: Cyprinidae), representing a sympatric species pair, from the Ayeyarwady drainage, Myanmar. Raffles Bulletin of Zoology, 69, 80–101.
49. Cui, G.H., Chu, X.L., 1990. Differentiation and distribution of the cyprinid fish *Percocypris pingi* (Tchang). Acta Zootaxonomica Sinica, 15(1), 118–123. (In Chinese, English Abstract)
50. Darshan, A., Vishwanath, W., Mahanta, P.C., Barat, A., 2011. *Mystus ngasep*, a new catfish species (Teleostei: Bagridae) from the headwaters of Chindwin drainage in Manupur, India. Journal of Threatened Taxa, 3(11), 2177–2183.
51. Datta, N.C., Chaudhuri, S., 1993. Two new glassfishes from Myanmar (Burma) (Perciformes: Ambassidae). Indian Biologist, 25(1), 1–4.
52. Doi, A., Taki, Y., 1994. A new cyprinid fish, *Hampala salweenensis*, from the Mae Pai River system, Salween Basin, Thailand. Japanese Journal of Ichthyology, 40(4), 405–412.
53. Endruweit, M., 2014. *Schistura megalodon* species nova, a new river loach from the Irrawaddy basin in Dehong, Yunnan, China (Teleostei: Cypriniformes: Nemacheilidae). Zoological Research, 35(5), 353–361.
54. Endruweit, M., 2017a. A new *Physoschistura* from a Salween affluent in western Yunnan (Teleostei: Nemacheilidae). Zootaxa, 4263(2), 378–386.
55. Endruweit, M., 2017b. A new *Schistura* from the Salween basin in western Yunnan (Teleostei: Nemacheilidae). Zootaxa 4243 (no. 2): 394–400.
56. Endruweit, M., 2017c. Description of a new dwarf snakehead (Perciformes: Channidae) from western Yunnan. Vertebrate Zoology, 67(2), 173–178.
57. Fang, F., 1998. *Danio kyathit*, a new species of cyprinid fish from Myitkyina, northern Myanmar. Ichthyological Exploration of Freshwaters, 8(3), 273–280.
58. Ferraris, C.J., 2004. A new species of the Asian schilbid catfish genus *Clupisoma* from Myanmar, with a redescription of *Clupisoma prateri* Hora (Osteichthyes: Siluriformes: Schilbidae). Zootaxa, 437, 1–10.
59. Ferraris, C.J., Britz, R., 2005. A diminutive new species of *Glyptothorax* (Siluriformes: Sisoridae) from the upper Irrawaddy River basin, Myanmar, with comments on sisorid and erethistid phylogenetic relationships. Ichthyological Exploration of Freshwaters, 16(4), 375–383.
60. Ferraris, C.J., Runge K.E., 1999. Revision of the South Asian bagrid catfish genus *Sperata*, with the description of a new species from Myanmar. Proceedings of the California Academy of Sciences, 51(10), 397–424.
61. Ferraris, C.J., Vari, R.P., 2007. Revision of catfishes of the genus *Eutropiichthys*, with the description of two new species (Siluriformes: Schilbidae). Copeia, 2007(4), 866–885.
62. Geetakumari, K., Basudha, C., 2012. *Parambassis waikhomi*, a new species of glassfish (Teleostei: Ambassidae) from Loktak Lake, northeastern India. Journal of Threatened Taxa, 4(14), 3327–3332.
63. Havird, J.C., Page, L.M., 2010. A revision of *Lepidocephalichthys* (Teleostei: Cobitidae) with descriptions of two new species from Thailand, Laos, Vietnam, and Myanmar. Copeia, 2010(1), 137–159.
64. He, J.C., Huang, K.W., Li, H., 1995. On numerical classification of fishes of the genus *Platytropius* with descriptions of one new species. Journal of Yunnan University (Natural Science), 17(3), 278–283.
65. He, S.P., 1996. A new species of the genus *Gagata* (Pisces: Sisoridae). Acta Zootaxonomica Sinica, 21(3), 380–382. (In Chinese, English abstract.)
66. Jiang W.S., Du, L.N., Jiang, Y.E., Yang, J.X., Chen, X.Y., 2010. Fish Composition，Fauna and Life History of Ruili River Drainage. Journal of Hydroecology, 3(5), 1–9. (in Chinese, with English abstract).
67. Jiang, W.S., Chen, X.Y., Yang, J.X., 2010. A new species of sisorid catfish genus *Glyptothorax* (Teleostei: Sisoridae) from Salween drainage of Yunnan, China. Environmental Biology of Fishes, 87(2), 125–133.
68. Jiang, W.S., Ng, H.H., Yang, J.X., Chen, X.Y., 2012. A taxonomic review of the catfish identified as *Glyptothorax zanaensis* (Teleostei: Siluriformes: Sisoridae), with the descriptions of two new species. Zoological Journal of the Linnean Society, 165(2), 363–389.
69. Jiang, Y.E., Chen, X.Y., Yang, J.X., 2008. *Microrasbora* Annandale, a new genus record in China, with description of a new species (Teleostei: Cyprinidae). Environmental Biology of Fishes, 83, 299–304.
70. Kong, D.P., Chen, X.Y., Yang, J.X., 2007. Two new species of the sisorid genus *Oreoglanis* Smith from Yunnan, China (Teleostei: Sisoridae). Environmental Biology of Fishes, 78, 223–230.
71. Kosygin, L., Vishwanath, W., 1998. A new cyrpinid fish *Garra compressus* from Manipur, India. Journal of Freshwater Biology, 10(1–2), 45–48.
72. Kottelat, M., 1990. Indochinese nemacheilines. A revision of nemacheiline loaches (Pisces: Cypriniformes) of Thailand, Burma, Laos, Cambodia and southern Viet Nam. München: Verlag Dr. Friedrich Pfeil, 1–262.
73. Kottelat, M., 2003. *Parambassis pulcinella*, a new species of glassperch (Teleostei: Ambassidae) from the Ataran River basin (Myanmar), with comments on the family-group names Ambassidae, Chandidae and Bogodidae. Ichthyological Exploration of Freshwaters, 14(1), 9–18.
74. Kottelat, M., 2004. *Botia kubotai*, a new species of loach (Teleostei: Cobitidae) from the ataran River basin (Myanmar), with comments on botiinae nomenclature and diagnosis of a new genus. Zootaxa, 401, 1–18.
75. Kottelat, M., 2017. A new genus and three new species of nemacheilid loaches from northern Irrawaddy drainage, Myanmar (Teleostei: Cypriniformes). Raffles Bulletin of Zoology, 65, 80–99.
76. Kottelat, M., 2017. *Lepidocephalichthys eleios*, a new loach from Lake Indawgyi basin, Kachin State, Myanmar (Teleostei: Cobitidae). Raffles Bulletin of Zoology, 65, 707–714.
77. Kottelat, M., 2017. *Schistura indawgyiana*, a new loach from Lake Indawgyi basin, Myanmar (Teleostei: Nemacheilidae). Ichthyological Exploration of Freshwaters, 28(1), 1–8.
78. Kottelat, M., 2018. *Mustura celata*, a new genus and species of loaches from northern Myanmar, and an overview of *Physoschistura* and related taxa (Teleostei: Nemacheilidae). Ichthyological Exploration of Freshwaters, 28(4), 289–314.
79. Kottelat, M., Witte, K.E., 1999. Two new species of *Microrasbora* from Thailand and Myanmar, with two new generic names for small southeast Asian cyprinid fishes (Teleostei: Cyprinidae). Journal of South Asian Natural History, 4(1), 49–56.
80. Kullander, S.O., 2008. Five new species of *Puntius* from Myanmar (Teleostei: Cyprinidae). Ichthyological Exploration of Freshwaters, 19(1), 59–84.
81. Kullander, S.O., 2012. Description of *Danio flagrans*, and redescription of *D. choprae*, two closely related species from the Ayeyarwaddy River drainage in northern Myanmar (Teleostei: Cyprinidae). Ichthyological Exploration of Freshwaters, 23(3), 245–262.
82. Kullander, S.O., 2017. *Devario fangae* and *Devario myitkyinae*, two new species of danionin cyprinids from northern Myanmar (Teleostei: Cyprinidae: Danioninae). Zootaxa, 4227(3), 407–421.
83. Kullander, S.O., Britz, R., 2002. Revision of the family Badidae (Teleostei: Perciformes), with description of a new genus and ten new species. Ichthyological Exploration of Freshwaters, 13(4), 295–372.
84. Kullander, S.O., Britz, R., 2008. *Puntius padamya*, a new species of cyprinid fish from Myanmar (Teleostei: Cyprinidae). Electronic Journal of Ichthyology, Bulletin of the European Ichthyology Society, 2, 56–66.
85. Kullander, S.O., Britz, R., Fang, F., 2000. *Pillaia kachinica*, a new chaudhuriid fish from Myanmar, with observations on the genus *Garo* (Teleostei: Chaudhuriidae). Ichthyological Exploration of Freshwaters, 11(4), 327–334.
86. Kullander, S.O., Fang, F., 2004. Seven new species of *Garra* (Cyprinidae: Cyprininae) from the Rakhine Yoma, southern Myanmar. Ichthyological Exploration of Freshwaters, 15(3), 257–278.
87. Kullander, S.O., Fang, F., 2005. Two new species of *Puntius* from northern Myanmar (Teleostei: Cyprinidae). Copeia 2005(2), 290–302.
88. Kullander, S.O., Fang, F., 2009. *Danio tinwini*, a new species of spotted danio from northern Myanmar (Teleostei: Cyprinidae). Ichthyological Exploration of Freshwaters, 20(3), 223–228.
89. Kullander, S.O., Liao, T.Y., Fang, F., 2009. *Danio quagga*, a new species of striped danio from western Myanmar (Teleostei: Cyprinidae). Ichthyological Exploration of Freshwaters, 20(3), 193–199.
90. Kullander, S.O., Norén, M., 2016. *Danio htamanthinus* (Teleostei: Cyprinidae), a new species of miniature cyprinid fish from the Chindwin River in Myanmar. Zootaxa, 4178(4), 535–546.
91. Kullander, S.O., Norén, M., 2022. The real *Devario browni* from the Irrawaddy River basin, and the new *Devario ahlanderi* from the Salween River basin in Myanmar (Teleostei: Cyprinidae: Danioninae). Zootaxa, 5100(1), 54–72.
92. Kullander, S.O., Rahman, Md. M., Norén, M., Mollah, A.R., 2018. *Laubuka tenella*, a new species of cyprinid fish from southeastern Bangladesh and southwestern Myanmar (Teleostei, Cyprinidae, Danioninae). ZooKeys, 742, 105–126.
93. Li, X., Che. X.J.. Zhou. W.. 2019. Loaches of *Homatula* (Teleostei: Nemacheilidae) from the upper Salween River in Yunnan, China with description of three new species. Zootaxa. 4711(2). 330–348.
94. Lin, F., Chen, Z.Y., Myint, K.M., Chen, X.Y., 2023. *Paracanthocobitis putaoensis*, a new loach species (Cypriniformes: Nemacheilidae) from the Irrawaddy basin in northern Myanmar. Zootaxa, 5227(2), 265–278.
95. Linthoingambi, I., Vishwanath, W., 2007. Two new fish species of the genus *Puntius* Hamilton (Cyprinidae) from Manipur, India, with notes on *P. ticto* (Hamilton) and *P. stoliczkanus* (Day). Zootaxa, 1450, 45–56.
96. Linthoingambi, I., Vishwanath, W., 2008. Two new catfish species of the genus *Amblyceps* from Manipur, India (Teleostei: Amblycipitidae). Ichthyological Exploration of Freshwaters, 19(2), 167–174.
97. Lokeshwor, Y., Vishwanath, W., 2012. A new loach of the genus *Physoschistura* Bănărescu & Nalbant (Teleostei: Nemacheilidae) from Chindwin basin, Manipur, India. Zootaxa, 3586, 95–102.
98. Lokeshwor, Y., Vishwanath, W., 2012. *Physoschistura chindwinensis*, a new balitorid loach from Chindwin basin, Manipur, India. Ichthyological Research, 59(3), 230–234.
99. Luo, Z.J., Chen, X.Y., 2020. *Exostoma dulongensis*, a new glyptosternine catfish from the Irrawaddy basin, Yunnan, China (Siluriformes: Sisoridae). Zootaxa, 4802(1), 99–110.
100. Menon, A.G.K., 1992. The fauna of India and the adjacent countries. Pisces. Vol. IV. Teleostei - Cobitoidea. Part 2. Cobitidae. viii + 1–113, Pls. 1–10.
101. Menon, A.G.K., Devi, K.R., Vishwanath, W., 2000. A new species of *Puntius* (Cyprinidae: Cyprininae) from Manipur, India. Journal of the Bombay Natural History Society, 97(2), 263–268.
102. Musikasinthorn, P., 1998. *Channa panaw*, a new channid fish from the Irrawaddy and Sittang River basins, Myanmar. Ichthyological Research, 45(4), 355–362.
103. Nebeshwar, K., Vishwanath, W., 2015. Two new species of *Garra* (Pisces: Cyprinidae) from the Chindwin River basin in Manipur, India, with notes on some nominal *Garra* species of the Himalayan foothills. Ichthyological Exploration of Freshwaters, 25(4), 305–321.
104. Ng, H.H., 2004. Two new glyptosternine catfishes (Teleostei: Sisoridae) from Vietnam and China. Zootaxa, 428, 1–12.
105. Ng, H.H., 2005. *Amblyceps carinatum*, a new species of hillstream catfish from Myanmar (Teleostei: Amblycipitidae). Raffles Bulletin of Zoology, 53(2), 243–249.
106. Ng, H.H., 2008. *Batasio procerus*, a new species of catfish from northern Myanmar (Siluriformes: Bagridae). Ichthyological Exploration of Freshwaters, 19(1), 1–6.
107. Ng, H.H., 2018. *Exostoma ericinum*, a new glyptosternine catfish from southwestern China (Teleostei: Siluriformes: Sisoridae). Zootaxa, 4420(3), 405–414.
108. Ng, H.H., Ferraris, C.J., 2000. A review of the genus *Hemibagrus* in southern Asia, with descriptions of two new species. Proceedings of the California Academy of Sciences, 52(11), 125–142.
109. Ng, H.H., Ferraris, C.J., Neely, D.A., 2012. The catfish genus *Erethistoides* (Siluriformes: Sisoridae) in Myanmar, with descriptions of three new species. Zootaxa, 3254, 55–68.
110. Ng, H.H., Jiang, W.S., Chen, X.Y., 2012. *Glyptothorax lanceatus*, a new species of sisorid catfish (Teleostei: Siluriformes) from southwestern China. Zootaxa, 3250, 54–62.
111. Ng, H.H., Kottelat M., 2004. *Akysis vespa*, a new species of catfish (Siluriformes: Akysidae) from the Ataran River drainage (Myanmar). Ichthyological Exploration of Freshwaters, 15(3), 193–200.
112. Ng, H.H., Kottelat M., 2005. *Caelatoglanis zonatus*, a new genus and species of the Erethistidae (Teleostei: Siluriformes) from Myanmar, with comments on the nomenclature of *Laguvia* and *Hara* species. Ichthyological Exploration of Freshwaters, 16(1), 13–22.
113. Ng, H.H., Kottelat M., 2007. A review of the catfish genus *Hara*, with the description of four new species (Siluriformes: Erethistidae). Revue Suisse de Zoologie, 114(3), 471–505.
114. Ng, H.H., Kottelat M., 2008. *Batasio feruminatus*, a new species of bagrid catfish from Myanmar (Siluriformes: Bagridae), with notes on the identity of *B. affinis* and *B. fluviatilis*. Ichthyological Exploration of Freshwaters, 18(4), 289–300.
115. Ng, H.H., Kottelat M., 2008. *Glyptothorax rugimentum*, a new species of catfish from Myanmar and western Thailand (Teleostei: Sisoridae). Raffles Bulletin of Zoology, 56(1), 129–134.
116. Ng, H.H., Kottelat M., 2009. A new species of *Mystus* from Myanmar (Siluriformes: Bagridae). Copeia, 2009(2), 245–250.
117. Ng, H.H., Kottelat M., 2018. A new Glyptosternine catfish from northern Myanmar (Teleostei: Siluriformes: Sisoridae). Copeia, 106(1), 63–69.
118. Ng, H.H., Kottelat M., 2022. A new glyptosternine catfish from Myanmar (Actinopterygii: Siluriformes: Sisoridae). Ichthyology & Herpetology, 110(2), 262–267.
119. Ng, H.H., Kottelat M.,2018. *Amblyceps improcerum*, a new sisoroid catfish from Kachin State, Myanmar (Teleostei: Siluriformes: Amblycipitidae). Environmental Biology of Fishes, 101(3), 459–467.
120. Ng, H.H., Kottelat, M., 2000. A review of the genus *Amblyceps* (Osteichthyes: Amblycipitidae) in Indochina, with descriptions of five new species. Ichthyological Exploration of Freshwaters, 11(4), 335–348.
121. Ng, H.H., Kullander, S.O., 2013. *Glyptothorax igniculus*, a new species of sisorid catfish (Teleostei: Siluriformes) from Myanmar. Zootaxa, 3681(5), 552–562.
122. Ng, H.H., Rainboth, W.J., 2001. A review of the sisorid catfish genus *Oreoglanis* (Siluriformes: Sisoridae) with descriptions of four new species. Occasional Papers of the Museum of Zoology University of Michigan, 732, 1–34.
123. Pethiyagoda, R., Gill, A.C., 2012. Description of two new species of sea bass (Teleostei: Latidae: *Lates*) from Myanmar and Sri Lanka. Zootaxa, 3314, 1–16.
124. Premananda, N., Kosygin, L., Saidullah, B., 2015. *Glyptothorax senapatiensis*, a new species of catfish (Teleostei: Sisoridae) from Manipur, India. Ichthyological Exploration of Freshwaters, 25(4), 323–329.
125. Qin, T., Maung, K.W., Chen, X.Y., 2019. *Opsarius putaoensis*, a new species of subfamily Danioninae (Actinopterygii, Cyprinidae) from the Irrawaddy River basin in northern Myanmar. Zootaxa, 4615(3), 585–593.
126. Qin, T., Kottelat, M., Kyaw, Y.M.M., Chen, X., 2022. *Mustura yangi*, a new species of loach (Teleostei: Nemacheilidae) from Putao, Irrawaddy drainage, northern Myanmar. Ichthyological Exploration of Freshwaters, IEF-1181, 1–11.
127. Rainboth, W.J., 1996. The taxonomy, systematics, and zoogeography of *Hypsibarbus*, a new genus of large barbs (Pisces, Cyprinidae) from the rivers of southeastern Asia. University of California Publications in Zoology, 129, 1–199.
128. Ramananda, Y., Vishwanath, W., 2014. *Devario deruptotalea*, a new species of cyprinid fish from Manipur, India (Teleostei: Cyprinidae). Zootaxa, 3827(1), 78–86.
129. Roberts, T.R., 1995. Systematic revision of tropical Asian freshwater glassperches (Ambassidae), with descriptions of three new species. Natural History Bulletin of the Siam Society, 42, 263–290.
130. Roberts, T.R., 1997. Systematic revision of the tropical Asian labeoin cyprinid fish genus *Cirrhinus*, with descriptions of new species and biological observations on *C. lobatus*. Natural History Bulletin of the Siam Society, 45(2), 171–203.
131. Roberts, T.R., 1998. Review of the tropical Asian cyprinid fish genus *Poropuntius*, with descriptions of new species and trophic morphs. Natural History Bulletin of the Siam Society, 46(1), 105–135.
132. Roberts, T.R., 1998. Systematic observations on tropical Asian medakas or ricefishes of the genus *Oryzias*, with descriptions of four new species. Ichthyological Research, 45(3), 213–224.
133. Roberts, T.R., 2001. *Ayarnangra estuarius*, a new genus and species of sisorid catfish from the Ayeyarwaddy basin, Myanmar. Natural History Bulletin of the Siam Society, 49(1), 81–87.
134. Roberts, T.R., 2007. *Makararaja chindwinensis*, a new genus and species of freshwater dasyatidid Pastinachine stingray from upper Myanmar. Natural History Bulletin of the Siam Society, 54(2), 285–293.
135. Roberts, T.R., 2007. The "Celestial Pearl Danio", a new genus and species of colourful minute cyprinid fish from Myanmar (Pisces: Cypriniformes). Raffles Bulletin of Zoology, 55(1), 131–140.
136. Roberts, T.R., Ferraris, C.J., 1998. Review of South Asian sisorid catfish genera *Gagata* and *Nangra*, with descriptions of a new genus and five new species. Proceedings of the California Academy of Sciences, 50(14), 315–345.
137. Roberts, T.R., Vidthayanon, C., 1991. Systematic revision of the Asian catfish family Pangasiidae, with biological observations and descriptions of three new species. Proceedings of the Academy of Natural Sciences of Philadelphia, 143, 97–143.
138. Selim, K., Vishwanath, W., 2001. A new freshwater cyprinid fish *Aspidoparia* from the Chatrickong River, Manipur, India. Journal of the Bombay Natural History Society, 98(2), 254–257.
139. Shangningam, B., Kosygin, L., 2020. A new sisorid catfish of the genus *Exostoma* Blyth from the Chindwin-Irrawaddy drainage in northeastern India (Teleostei: Siluriformes). Copeia, 108(3), 545–550.
140. Shangningam, B., Kosygin, L., 2021. *Psilorhynchus magnaoculus*, a new species of torrent minnow (Teleostei: Psilorhynchidae) from Myanmar. Records of the Zoological Survey of India, 121(3), 319–324.
141. Shangningam, B., Kosygin, L., 2022. *Glyptothorax yuensis*, a new species of sisorid catfish (Teleostei: Sisoridae) from Myanmar. Zootaxa, 5129(1), 118–128.
142. Shangningam, B., Vishwanath, W., 2012. Validation of *Garra namyaensis* Shangningam & Vishwanath, 2012 (Teleostei: Cyprinidae: Labeioninae). Ichthyological Exploration of Freshwaters, 2(1), 10.
143. Shangningam, B., Vishwanath, W., 2013. A new species of *Psilorhynchus* (Teleostei: Psilorhynchidae) from the Chindwin basin of Manipur, India. Zootaxa, 3694(4), 381–390.
144. Shangningam, B., Vishwanath, W., 2013. *Psilorhynchus maculatus*, a new species of torrent minnow from the Chindwin basin, Manipur, India (Teleostei: Psilorhynchidae). Ichthyological Exploration of Freshwaters, 24(1), 57–62.
145. Shangningam, B., Vishwanath, W., 2014. *Psilorhynchus ngathanu*, a new torrent minnow species (Teleostei: Psilorhynchidae) from the Chindwin Basin, Manipur, India. Ichthyological Research, 61, 27–31.
146. Shangningam, B., Vishwanath, W., 2015. Two new species of *Garra* from the Chindwin basin, India (Teleostei: Cyprinidae). Ichthyological Exploration of Freshwaters, 26(3), 263–272.
147. Shangningam, B., Vishwanath, W., 2016. *Psilorhynchus konemi*, a new species of torrent minnow from northeast India (Teleostei: Psilorhynchidae). Ichthyological Exploration of Freshwaters, 27(4), 289–296.
148. Shangningam, B., Vishwanath, W., 2018. *Pethia poiensis*, a new species of cyprinid fish from the Chindwin Basin of Manipur, India. Zootaxa, 4379(4), 585–593.
149. Shangningam, B., Lokeshwor, Y., Vishwanath, W., 2014. *Schistura phamhringi*, a new stone loach from Chindwin Basin in Manipur, India (Cypriniformes: Nemacheilidae). Zootaxa, 3786(2), 181–191.
150. Siebert, D.J., 1991. Revision of *Acanthopsoides* Fowler, 1934 (Cypriniformes: Cobitidae), with the description of new species. Japanese Journal of Ichthyology, 38(2), 97–114.
151. Singer, R.A., Page, L.M., 2015. Revision of the zipper loaches, *Acanthocobitis* and *Paracanthocobitis* (Teleostei: Nemacheilidae), with descriptions of five new species. Copeia, 103(2), 378–401.
152. Singer, R.A., Pfeiffer, J.M., Page, L.M., 2017. A revision of the *Paracanthocobitis zonalternans* (Cypriniformes: Nemacheilidae) species complex with descriptions of three new species. Zootaxa, 4324(1), 85–107.
153. Suvarnaraksha, A., 2010. Fish diversity in Salween watershed in Thai water. Maejo Univesity, https://doi: 10.13140/2.1.2699.7761
154. Vidthayanon, C., Saenjundaeng, P., Ng, H.H., 2009. Eight new species of the torrent catfish genus *Oreoglanis* (Teleosei: Sisoridae) from Thailand. Ichthyological Exploration of Freshwaters, 20(2), 127–156.
155. Vishwanath, W., 1993. On a collection of fishes of the genus *Garra* Hamilton from Manipur, India, with a description of a new species. Journal of Freshwater Biology, 5(1), 59–68.
156. Vishwanath, W., Darshan, A., 2007. Two new catfish species of the genus *Pseudecheneis* Blyth (Teleostei: Siluriformes) from northeastern India. Zoos' Print Journal, 22(3), 2627–2631.
157. Vishwanath, W., Joyshree, H., 2005. A new species of genus *Garra* Hamilton-Buchanan (Teleostei: Cyprinidae) from Manipur, India. Zoos' Print Journal, 20(4), 1832–1834.
158. Vishwanath, W., Kosygin, L., 1999. A new sisorid catfish of the genus *Myersglanis* Hora & Silas 1951, from Manipur, India. Journal of the Bombay Natural History Society, 96(2), 291–296.
159. Vishwanath, W., Kosygin, L., 2000. Fishes of the cyprinid genus *Semiplotus* Bleeker 1859, with description of a new species from Manipur, India. Journal of the Bombay Natural History Society, 97(1), 92–102.
160. Vishwanath, W., Kosygin, L., 2000. *Garra elongata*, a new species of the subfamily Garrinae from Manipur, India (Cyprinidae, Cypriniformes). Journal of the Bombay Natural History Society, 97(3), 408–414.
161. Vishwanath, W., Laisram, J., 2004. Two new species of *Puntius* Hamilton-Buchanan (Cypriniformes: Cyprinidae) from Manipur, India, with an account of *Puntius* species from the state. Journal of the Bombay Natural History Society, 101(1), 130–137.
162. Vishwanath, W., Laisram, J., 2005. A new species of *Rasbora* Bleeker (Cypriniformes: Cyprinidae) from Manipur, India. Journal of the Bombay Natural History Society, 101(3), 429–432.
163. Vishwanath, W., Linthoingambi, I., 2006. A new sisorid catfish of the genus *Glyptothorax* Blyth from Manipur, India. Journal of the Bombay Natural History Society, 102(2), 201–203.
164. Vishwanath, W., Linthoingambi, I., 2007. Fishes of the genus *Glyptothorax* Blyth (Teleostei: Sisoridae) from Manipur, India, with description of three new species. Zoos' Print Journal, 22(3), 2617–2626.
165. Vishwanath, W., Manojkumar, W., 1995. Fishes of the cyprinoid genus *Psilorhynchus* McClelland from Manipur, India, with description of a new species. Japanese Journal of Ichthyology, 42(3/4), 249–253.
166. Vishwanath, W., Shanta, K., 2005. A new fish species of the genus *Garra* Hamilton-Buchanan (Cypriniformes: Cyprinidae) from Manipur, India. Journal of the Bombay Natural History Society, 102(1), 86–88.
167. Vishwanath, W., Sharma, K.N., 2004. *Schistura reticulata*, a new species of balitorid loach from Manipur, India, with redescription of *S. chindwinica*. Ichthyological Exploration of Freshwaters, 15(4), 323–330.
168. Wu, Y.F., Wu, C.Z., 1992. The Fishes of the Qinghai-Xizang Plateau. Chengdu: Sichuan Publishing House of Science & Technology, 1–599. (In Chinese, English abstract).
169. Yang, J., Zheng, L.P., Chen, X.Y., Yang, J.X., 2013. Description of two new species and revision of *Schizothoroax* distributed in the Irrawaddy drainage area in China. Zoological Research, 34(4), 361–367. (in Chinese, English abstract).
170. Yang, J.X., Du, L.N., 2006. Chapter 17. Fish resources in the reserve. In: Yang, Y.M. Yunnan. Integrated Scientific Studies of Yunnan Tongbiguan Nature Reserve. Kunming: Yunnan Science Technology Press, Pp. 267–268. (in Chinese).
171. Yang, J.X., Chu, X.L., 1990. A new genus and a new species of Nemacheilinae from Yunnan Province, China. Zoological Research, 11(2), 109–114. (In Chinese, English abstract.)
172. Yang, L.P., Zhou, W., 2011. A review of the genus *Mastacembelus* (Perciformes, Mastacembelidae) in China with description of two new species and one new record. Acta Zootaxonomica Sinica, 36(2), 325–331.
173. Zhang, E, 2005. *Garra bispinosa*, a new species of cyprinid fish (Teleostei: Cypriniformes) from Yunnan, southwest China. Raffles Bulletin of Zoology, Suppl.,13: 9–15.
174. Zhang, E, 2006. *Garra rotundinasus*, a new species of cyprinid fish (Pisces: Teleostei) from the Upper Irrawaddy River basin, China. Raffles Bulletin of Zoology, 54(2), 447–453.
175. Zhang, E, Chen, Y.Y., 2002. *Garra tengchongensis*, a new cyprinid species from the upper Irrawaddy River basin in Yunnan, China (Pisces: Teleostei). Raffles Bulletin of Zoology, 50(2), 459–464.
176. Zhang, X.Y., Long, Y., Xiao, H., Chen, Z.M., 2016. First record of the catfish genus *Amblyceps* Blyth, 1858 from China with the description of a new species (Osteichthyes: Amblycipitidae). Folia Zoologica: international journal of vertebrate zoology, 65(2), 81–86.
177. Zheng, L.P., Yang, J.X., Chen, X.Y., 2012. *Schistura prolixifasciata*, a new species of loach (Teleostei: Nemacheilidae) from the Salween basin in Yunnan, China. Ichthyological Exploration of Freshwaters, 23(1), 63–68.
178. Zhou, W., Kottelat, M., 2005. *Schistura disparizona*, a new species of loach from Salween drainage in Yunnan (Teleostei: Balitoridae). Raffles Bulletin of Zoology, Suppl. 13, 17–20.
179. Zhou, W., Li, X., Thomson, A.W., 2011. A new genus of glyptosternine catfish (Siluriformes: Sisoridae) with descriptions of two new species from Yunnan, China. Copeia, 2011(2), 226–241.
180. Zhou, W., Li, X., Yang, Y., 2008. A review of the catfish genus *Pseudecheneis* (Siluriformes: Sisoridae) from China, with the description of four new species. Raffles Bulletin of Zoology, 56(1), 107–124.
181. Zhou, W., Yang, Y., Li, X., Li, M.H., 2007. A review of the catfish genus *Pseudexostoma* (Siluriformes: Sisoridae) with description of a new species from the upper Salween (Nujiang) Basin of China. Raffles Bulletin of Zoology, 55(1), 147–155.
