# Supplementary material for: Fish diversity and selection of taxa for conservation in the Salween and Irrawaddy Rivers, Southeast Asia
Source: Sci Rep. 2024 Jan 29;14:2393. doi: 10.1038/s41598-024-51205-5 (PMC10825156; doi:10.1038/s41598-024-51205-5)
Supplement: Supplementary file 4 — Supplementary Table S1. [file 41598_2024_51205_MOESM4_ESM.docx]

#### Table S1-1 The fish orders of the Salween river, and the percentage of the total number of genera and species represented by each order

| Order No. | Order | Genera | Species | % of total | |
| --- | --- | --- | --- | --- | --- |
|  |  |  |  | Genera | Species |
| 1 | RHINOPRISTIFORMES | 1 | 2 | 0.59 | 0.55 |
| 2 | ELOPIFORMES | 1 | 1 | 0.59 | 0.28 |
| 3 | ANGUILLIFORMES | 1 | 3 | 0.59 | 0.83 |
| 4 | OSTEOGLOSSIFORMES | 1 | 1 | 0.59 | 0.28 |
| 5 | CLUPEIFORMES | 6 | 10 | 3.53 | 2.76 |
| 6 | **CYPRINIFORMES** | **71** | **182** | **41.76** | **50.28** |
| 7 | **SILURIFORMES** | **35** | **77** | **20.59** | **21.27** |
| 8 | KURTIFORMES | 1 | 1 | 0.59 | 0.28 |
| 9 | **GOBIIFORMES** | **10** | **10** | **5.88** | **2.76** |
| 10 | SYNBRANCHIFORMES | 6 | 12 | 3.53 | 3.31 |
| 11 | **ANABANTIFORMES** | **8** | **18** | **4.71** | **4.97** |
| 12 | CARANGIFORMES | 2 | 5 | 1.18 | 1.38 |
| 13 | CICHLIFORMES | 2 | 9 | 1.18 | 2.49 |
| 14 | CYPRINODONTIFORMES | 1 | 1 | 0.59 | 0.28 |
| 15 | BELONIFORMES | 5 | 8 | 2.94 | 2.21 |
| 16 | MUGILIFORMES | 6 | 8 | 3.53 | 2.21 |
| 17 | CENTRARCHIFORMES | 1 | 1 | 0.59 | 0.28 |
| 18 | ACANTHURIFORMES | 9 | 9 | 5.29 | 2.49 |
| 19 | TETRAODONTIFORMES | 3 | 4 | 1.76 | 1.10 |
|  | **Total** | **170** | **362** | **100.0** | **100.00** |

Data in bold show the four main fish orders of the Salween river basin.

#### Table S1-2 The fish orders of the Irrawaddy river, and the percentage of the total numbers of genera and species represented by each order

| Order No. | Order | Genera | Species | % in total | |
| --- | --- | --- | --- | --- | --- |
|  |  |  |  | Genera | Species |
| 1 | CARCHARHINIFORMES | 2 | 3 | 1.04 | 0.60 |
| 2 | RHINOPRISTIFORMES | 1 | 1 | 0.52 | 0.20 |
| 3 | MYLIOBATIFORMES | 1 | 1 | 0.52 | 0.20 |
| 4 | ELOPIFORMES | 1 | 1 | 0.52 | 0.20 |
| 5 | ANGUILLIFORMES | 3 | 5 | 1.55 | 1.00 |
| 6 | OSTEOGLOSSIFORMES | 1 | 1 | 0.52 | 0.20 |
| 7 | CLUPEIFORMES | 9 | 13 | 4.66 | 2.59 |
| 8 | **CYPRINIFORMES** | **55** | **253** | **28.50** | **50.40** |
| 9 | **SILURIFORMES** | **40** | **100** | **20.73** | **19.92** |
| 10 | SYNGNATHIFORMES | 1 | 1 | 0.52 | 0.20 |
| 11 | KURTIFORMES | 1 | 1 | 0.52 | 0.20 |
| 12 | **GOBIIFORMES** | **25** | **28** | **12.95** | **5.58** |
| 13 | SYNBRANCHIFORMES | 9 | 19 | 4.66 | 3.78 |
| 14 | **ANABANTIFORMES** | **8** | **26** | **4.15** | **5.18** |
| 15 | CARANGIFORMES | 5 | 7 | 2.59 | 1.39 |
| 16 | CICHLIFORMES | 2 | 7 | 1.04 | 1.39 |
| 17 | CYPRINODONTIFORMES | 1 | 1 | 0.52 | 0.20 |
| 18 | BELONIFORMES | 5 | 7 | 2.59 | 1.39 |
| 19 | MUGILIFORMES | 7 | 9 | 3.63 | 1.79 |
| 20 | CENTRARCHIFORMES | 1 | 1 | 0.52 | 0.20 |
| 21 | ACANTHURIFORMES | 12 | 13 | 6.22 | 2.59 |
| 22 | TETRAODONTIFORMES | 3 | 4 | 1.55 | 0.80 |
|  | **Total** | **193** | **502** | **100.0** | **100.0** |

Data in bold show the four main fish orders of the Irrawaddy river basin.
