# Supplementary material for: Fish diversity and selection of taxa for conservation in the Salween and Irrawaddy Rivers, Southeast Asia
Source: Sci Rep. 2024 Jan 29;14:2393. doi: 10.1038/s41598-024-51205-5 (PMC10825156; doi:10.1038/s41598-024-51205-5)
Supplement: Supplementary file 5 — Supplementary Table S2. [file 41598_2024_51205_MOESM5_ESM.docx]

#### Table S2-1 The four main orders of fish (Cypriniformes, Siluriformes, Gobiiformes and Anabantiformes) in the Salween river, as well as the families, genera and species of these orders found in the Salween fish fauna

| Family No. | Order and family | Genera | Species | family % in the order | |
| --- | --- | --- | --- | --- | --- |
|  |  |  |  | Genera | Species |
|  | 6. CYPRINIFORMES |  |  |  |  |
| 8 | Botiidae | 2 | 5 | 2.82 | 2.75 |
| 9 | Cobitidae | 5 | 11 | 7.04 | 6.04 |
| 10 | Balitoridae | 5 | 9 | 7.04 | 4.95 |
| **11** | **Nemacheilidae** | **10** | **38** | **14.08** | **20.88** |
| 12 | Psilorhynchidae | 1 | 1 | 1.41 | 0.55 |
| **13** | **Cyprinidae** | **34** | **81** | **47.89** | **44.51** |
| **14** | **Danionidae** | **14** | **37** | **19.72** | **20.33** |
|  | **Subtotal** |  |  | **100.00** | **100.00** |
|  | 7. SILURIFORMES |  |  |  |  |
| 15 | Chacidae | 1 | 1 | 2.86 | 1.30 |
| 16 | Plotosidae | 1 | 1 | 2.86 | 1.30 |
| 17 | Ailiidae | 4 | 7 | 11.43 | 9.09 |
| 18 | Horabagridae | 1 | 1 | 2.86 | 1.30 |
| **19** | **Bagridae** | **6** | **13** | **17.14** | **16.88** |
| 20 | Akysidae | 1 | 1 | 2.86 | 1.30 |
| 21 | Amblycipitidae | 1 | 5 | 2.86 | 6.49 |
| **22** | **Sisoridae** | **11** | **36** | **31.43** | **46.75** |
| 23 | Pangasiidae | 1 | 1 | 2.86 | 1.30 |
| 24 | Siluridae | 4 | 5 | 11.43 | 6.49 |
| 25 | Clariidae | 1 | 3 | 2.86 | 3.90 |
| 26 | Heteropneustidae | 1 | 1 | 2.86 | 1.30 |
| 27 | Ariidae | 2 | 2 | 5.71 | 2.60 |
|  | **Subtotal** |  |  | **100.00** | **100.00** |
|  | 12. GOBIIFORMES |  |  |  |  |
| 29 | Eleotridae | 1 | 1 | 10.00 | 10.00 |
| 30 | Gobiidae | 9 | 9 | 90.00 | 90.00 |
|  | **Subtotal** | **10** | **10** | **100.00** | **100.00** |
|  | 11. ANABANTIFORMES |  |  |  |  |
| 34 | Anabantidae | 1 | 1 | 12.50 | 5.56 |
| 35 | Osphronemidae | 4 | 8 | 50.00 | 44.44 |
| 36 | Channidae | 1 | 7 | 12.50 | 38.89 |
| 37 | Nandidae | 1 | 1 | 12.50 | 5.56 |
| 38 | Badidae | 1 | 1 | 12.50 | 5.56 |
|  | **Subtotal** |  |  | **100.00** | **100.00** |

Family No. corresponds to numbers in Supplementary Appendix 1-1.

#### Table S2-2 The four main orders of fish (Cypriniformes, Siluriformes, Gobiiformes and Anabantiformes) in the Irrawaddy river, as well as the families, genera and species of these orders found in the Irrawaddy fish fauna

| Family No. | Order and family | Genera | Species | family % in the order | |
| --- | --- | --- | --- | --- | --- |
|  |  |  |  | Genera | Species |
|  | 8. CYPRINIFORMES |  |  |  |  |
| 13 | Botiidae | 2 | 4 | 3.64 | 1.58 |
| 14 | Cobitidae | 4 | 16 | 7.27 | 6.32 |
| 15 | Balitoridae | 4 | 6 | 7.27 | 2.37 |
| **16** | **Nemacheilidae** | **7** | **45** | **12.73** | **17.79** |
| 17 | Psilorhynchidae | 1 | 14 | 1.82 | 5.53 |
| **18** | **Cyprinidae** | **23** | **107** | **41.82** | **42.29** |
| **19** | **Danionidae** | **14** | **61** | **25.45** | **24.11** |
|  | **Subtotal** |  |  | **100.00** | **100.00** |
|  | 9. SILURIFORMES |  |  |  |  |
| 20 | Chacidae | 1 | 1 | 2.50 | 1.00 |
| 21 | Plotosidae | 1 | 1 | 2.50 | 1.00 |
| 22 | Ailiidae | 4 | 6 | 10.00 | 6.00 |
| 23 | Horabagridae | 1 | 1 | 2.50 | 1.00 |
| **24** | **Bagridae** | **6** | **20** | **15.00** | **20.00** |
| 25 | Akysidae | 1 | 2 | 2.50 | 2.00 |
| 26 | Amblycipitidae | 1 | 6 | 2.50 | 6.00 |
| **27** | **Sisoridae** | **14** | **47** | **35.00** | **47.00** |
| 28 | Pangasiidae | 1 | 2 | 2.50 | 2.00 |
| 29 | Siluridae | 3 | 5 | 7.50 | 5.00 |
| 30 | Clariidae | 1 | 1 | 2.50 | 1.00 |
| 31 | Heteropneustidae | 1 | 1 | 2.50 | 1.00 |
| 32 | Ariidae | 5 | 7 | 12.50 | 7.00 |
|  | **Subtotal** |  |  | **100.00** | **100.00** |
|  | 12. GOBIIFORMES |  |  |  |  |
| 35 | Eleotridae | 3 | 3 | 12.00 | 10.71 |
| 36 | Gobiidae | 22 | 25 | 88.00 | 89.29 |
|  | **Subtotal** | **25** | **28** | **100.00** | **100.00** |
|  | 14. ANABANTIFORMES |  |  |  |  |
| 41 | Anabantidae | 1 | 1 | 12.50 | 3.85 |
| 42 | Osphronemidae | 3 | 5 | 37.50 | 19.23 |
| 43 | Channidae | 1 | 10 | 12.50 | 38.46 |
| 44 | Nandidae | 1 | 1 | 12.50 | 3.85 |
| 45 | Badidae | 2 | 9 | 25.00 | 34.62 |
|  | **Subtotal** |  |  | **100.00** | **100.00** |

Family No. corresponds to numbers in Supplementary Appendix 1-2.
