# Supplementary material for: Fish diversity and selection of taxa for conservation in the Salween and Irrawaddy Rivers, Southeast Asia
Source: Sci Rep. 2024 Jan 29;14:2393. doi: 10.1038/s41598-024-51205-5 (PMC10825156; doi:10.1038/s41598-024-51205-5)
Supplement: Supplementary file 6 — Supplementary Table S3. [file 41598_2024_51205_MOESM6_ESM.docx]

#### Table S3-1 The fish families (subfamilies) with five species or more found in the Salween River

| Tab. 3 No. | Family No. | Family/subfamily | Globe Total | | Salween River | | % in total of Salween River | | Ranking No. in Irrawaddy River |
| --- | --- | --- | --- | --- | --- | --- | --- | --- | --- |
|  |  |  | Genera | Species | Genera | Species | Genera | Species |  |
| 1 | 11 | Nemacheilidae | 48 | 800 | 10 | 38 | 5.88 | 10.50 | 1 |
| 2 | 13A | Labeoninae | 53 | 521 | 10 | 22 | 5.88 | 6.08 | 2 |
| 3 | 14C | Danioninae | 10 | 99 | 7 | 20 | 4.12 | 5.52 | 3 |
| 4 | 22A | Sisorinae | 12 | 195 | 5 | 19 | 2.94 | 5.25 | 5 |
| 5 | 13B | Torinae | 33 | 228 | 8 | 18 | 4.71 | 4.97 | 18 |
| 6 | 13D | Cyprininae | 14 | 223 | 3 | 17 | 1.76 | 4.70 | 16 |
| 7 | 22B | Glyptosterninae | 14 | 111 | 6 | 17 | 3.53 | 4.70 | 6 |
| 8 | 13C | Smiliogastrinae | 30 | 478 | 8 | 15 | 4.71 | 4.14 | 4 |
| 9 | 19 | Bagridae | 19 | 228 | 6 | 13 | 3.53 | 3.59 | 7 |
| 10 | 9 | Cobitidae | 18 | 231 | 5 | 11 | 2.94 | 3.04 | 8 |
| 11 | 14A | Chedrinae | 17 | 131 | 4 | 10 | 2.35 | 2.76 | 9 |
| 12 | 10 | Balitoridae | 16 | 104 | 5 | 9 | 2.94 | 2.49 | 22 |
| 13 | 41 | Ambassidae | 7 | 54 | 2 | 9 | 1.18 | 2.49 | 21 |
| 14 | 47 | Mugilidae | 26 | 78 | 6 | 8 | 3.53 | 2.21 | 15 |
| 15 | 17 | Ailiidae | 7 | 25 | 4 | 7 | 2.35 | 1.93 | 23 |
| 16 | 31 | Mastacembelidae | 3 | 93 | 2 | 7 | 1.18 | 1.93 | 13 |
| 17 | 36 | Channidae | 2 | 56 | 1 | 7 | 0.59 | 1.93 | 14 |
| 18 | 8A | Botiinae | 6 | 32 | 2 | 5 | 1.18 | 1.38 |  |
| 19 | 13F | Schizothoracinae | 4 | 76 | 2 | 5 | 1.18 | 1.38 | 11 |
| 20 | 21 | Amblycipitidae | 4 | 47 | 1 | 5 | 0.59 | 1.38 | 24 |
| 21 | 24 | Siluridae | 13 | 106 | 4 | 5 | 2.35 | 1.38 | 29 |
| 22 | 35B | Trichogastrinae | 2 | 9 | 2 | 5 | 1.18 | 1.38 |  |
|  |  | **Subtotal** | **358** | **3925** | **103** | **272** | **60.59** | **75.14** |  |

Family No. corresponds to the numbers in Supplementary Appendix 1-1. The family name is written to the left of the cells and the subfamily name is to the right. The Cypriniformes comprise 11 families (subfamilies), Nemacheilidae, Labeoninae, Danioninae, Torinae. Cyprininae, Smiliogastrinae, Cobitidae, Chedrinae, Balitoridae, Botiinae, and Schizothoracinae. The Siluriformes comprise six families (subfamilies), Sisorinae, Glyptosterninae, Bagridae, Ailiidae, Amblycipitidae, and Siluridae. The Anabantiformes comprise two families (subfamilies), Channidae and Trichogastrinae. The other three families (subfamilies), Ambassidae, Mugilidae and Mastacembelidae, belong to other orders. Global Total is taken from Fricke, R., Eschmeyer, W.N. & Van der Laan, R. (eds.) Eschmeyer's catalog of fishes: genera, species, references, available from: <http://researcharchive.calacademy.org/research/ichthyology/catalog/fishcatmain.asp> (Online Version, accessed and updated 2 May 2023) (2023b).

#### Table S3-2 The fish families (subfamilies) with five species or more found in the Irrawaddy river

| Tab. 3 No. | Family No. | Family/subfamily | Total | | Irrawaddy River | | % in total of Irrawaddy River | | No. in Salween River |
| --- | --- | --- | --- | --- | --- | --- | --- | --- | --- |
|  |  |  | Genera | Species | Genera | Species | Genera | Species |  |
| 1 | 16 | Nemacheilidae | 48 | 800 | 7 | 45 | 3.63 | 8.96 | 1 |
| 2 | 18A | Labeoninae | 53 | 521 | 5 | 41 | 2.59 | 8.17 | 2 |
| 3 | 19C | Danioninae | 10 | 99 | 5 | 35 | 2.59 | 6.97 | 3 |
| 4 | 18C | Smiliogastrinae | 30 | 478 | 7 | 32 | 3.63 | 6.37 | 8 |
| 5 | 27A | Sisorinae | 12 | 195 | 7 | 26 | 3.63 | 5.18 | 4 |
| 6 | 27B | Glyptosterninae | 14 | 111 | 7 | 21 | 3.63 | 4.18 | 7 |
| 7 | 24 | Bagridae | 19 | 228 | 6 | 20 | 3.11 | 3.98 | 9 |
| 8 | 14 | Cobitidae | 18 | 231 | 4 | 16 | 2.07 | 3.19 | 10 |
| 9 | 19A | Chedrinae | 17 | 131 | 6 | 16 | 3.11 | 3.19 | 11 |
| 10 | 17 | Psilorhynchidae | 1 | 32 | 1 | 14 | 0.52 | 2.79 |  |
| 11 | 18F | Schizothoracinae | 4 | 76 | 1 | 14 | 0.52 | 2.79 | 19 |
| 12 | 45 | Badidae | 2 | 35 | 2 | 9 | 1.04 | 1.79 |  |
| 13 | 37 | Mastacembelidae | 3 | 93 | 2 | 11 | 1.04 | 2.19 | 16 |
| 14 | 43 | Channidae | 2 | 56 | 1 | 10 | 0.52 | 1.99 | 17 |
| 15 | 56 | Mugilidae | 26 | 78 | 7 | 9 | 3.63 | 1.79 | 14 |
| 16 | 18D | Cyprininae | 33 | 228 | 4 | 8 | 2.07 | 1.59 | 6 |
| 17 | 36A | Gobionellinae | 73 | 501 | 7 | 8 | 3.63 | 1.59 |  |
| 18 | 18B | Torinae | 14 | 223 | 2 | 7 | 1.04 | 1.39 | 5 |
| 19 | 32 | Ariinae | 32 | 148 | 5 | 7 | 2.59 | 1.39 |  |
| 20 | 36D | Gobiinae | 151 | 1273 | 7 | 7 | 3.63 | 1.39 |  |
| 21 | 50 | Ambassidae | 7 | 54 | 2 | 7 | 1.04 | 1.39 | 13 |
| 22 | 15 | Balitoridae | 16 | 104 | 4 | 6 | 2.07 | 1.20 | 12 |
| 23 | 22 | Ailiidae | 7 | 25 | 4 | 6 | 2.07 | 1.20 | 15 |
| 24 | 26 | Amblycipitidae | 4 | 47 | 1 | 6 | 0.52 | 1.20 | 20 |
| 25 | 36C | Amblyopinae | 15 | 37 | 4 | 6 | 2.07 | 1.20 |  |
| 26 | 10 | Clupeidae | 7 | 14 | 4 | 5 | 2.07 | 1.00 |  |
| 27 | 19B | Rasborinae | 11 | 124 | 2 | 5 | 1.04 | 1.00 |  |
| 28 | 19D | Esominae | 1 | 9 | 1 | 5 | 0.52 | 1.00 |  |
| 29 | 29 | Siluridae | 13 | 106 | 3 | 5 | 1.55 | 1.00 | 21 |
| 30 | 62 | Sciaenidae | 68 | 299 | 4 | 5 | 2.07 | 1.00 |  |
|  |  | **Subtotal** | **711** | **6356** | **122** | **412** | **63.21** | **82.07** |  |

Family No. corresponds to numbers in Supplementary Appendix 1-2. The family name is written to the left of the cells and the subfamily name is to the right. The Cypriniformes comprise 13 families (subfamilies), Nemacheilidae, Labeoninae, Danioninae, Smiliogastrinae, Cobitidae, Chedrinae, Psilorhynchidae, Schizothoracinae, Cyprininae, Torinae, Balitoridae, Rasborinae, and Esominae. The Siluriformes comprise seven families (subfamilies), Sisorinae, Glyptosterninae, Bagridae, Ariinae, Ailiidae, Amblycipitidae, and Siluridae. The Anabantiformes comprise two families (subfamilies), Badidae and Channidae. The Gobiiformes comprise three families (subfamilies), Gobionellinae, Gobiinae, and Amblyopinae. The other five families (subfamilies), Mastacembelidae, Mugilidae, Ambassidae, Clupeidae, and Sciaenidae, belong to other orders.
