# Supplementary material for: Fish diversity and selection of taxa for conservation in the Salween and Irrawaddy Rivers, Southeast Asia
Source: Sci Rep. 2024 Jan 29;14:2393. doi: 10.1038/s41598-024-51205-5 (PMC10825156; doi:10.1038/s41598-024-51205-5)
Supplement: Supplementary file 7 — Supplementary Table S4. [file 41598_2024_51205_MOESM7_ESM.docx]

#### Table S4-1 The fish genera with three species or more from the Salween river

| Tab. 5 No. | Genus No. | Genus | Species | | % of total species of Salween River | VFFP for genus | No. in Irrawaddy River |
| --- | --- | --- | --- | --- | --- | --- | --- |
|  |  |  | Total | Salween River |  |  |  |
| 1 | 31 | *Schistura* | 235 | 17 | 4.70 | 7.23 | 2 |
| 2 | 45 | *Neolissochilus* | 31 | 11 | 3.04 | 35.48 | 21 |
| 3 | 101 | *Glyptothorax* | 119 | 11 | 3.04 | 9.24 | 5 |
| 4 | 61 | *Poropuntius* | 35 | 8 | 2.21 | 22.86 | 27 |
| 5 | 76 | *Devario* | 44 | 8 | 2.21 | 18.18 | 4 |
| 6 | 145 | *Parambassis* | 21 | 8 | 2.21 | 38.10 | 19 |
| 7 | 38 | *Garra* | 189 | 7 | 1.93 | 3.70 | 1 |
| 8 | 139 | *Channa* | 53 | 7 | 1.93 | 13.21 | 10 |
| 9 | 40 | *Labeo* | 112 | 6 | 1.66 | 5.36 | 8 |
| 10 | 74 | *Danio* | 26 | 6 | 1.66 | 23.08 | 9 |
| 11 | 15 | *Lepidocephalichthys* | 19 | 5 | 1.38 | 26.32 | 11 |
| 12 | 46 | *Tor* | 17 | 5 | 1.38 | 29.41 | 51 |
| 13 | 69 | *Opsarius* | 32 | 5 | 1.38 | 15.63 | 15 |
| 14 | 96 | *Amblyceps* | 23 | 5 | 1.38 | 21.74 | 17 |
| 15 | 105 | *Oreoglanis* | 24 | 5 | 1.38 | 20.83 |  |
| 16 | 129 | *Mastacembelus* | 66 | 5 | 1.38 | 7.58 | 24 |
| 17 | 11 | *Botia* | 9 | 4 | 1.10 | 44.44 | 32 |
| 18 | 30 | *Physoschistura* | 8 | 4 | 1.10 | 50.00 |  |
| 19 | 32 | *Triplophysa* | 162 | 4 | 1.10 | 2.47 |  |
| 20 | 53 | *Puntius* | 44 | 4 | 1.10 | 9.09 | 26 |
| 21 | 65 | *Schizothorax* | 70 | 4 | 1.10 | 5.71 | 7 |
| 22 | 104 | *Exostoma* | 20 | 4 | 1.10 | 20.00 | 16 |
| 23 | 151 | *Oryzias* | 36 | 4 | 1.10 | 11.11 |  |
| 24 | 3 | *Anguilla* | 18 | 3 | 0.83 | 16.67 | 31 |
| 25 | 18 | *Balitora* | 17 | 3 | 0.83 | 17.65 |  |
| 26 | 23 | *Homatula* | 27 | 3 | 0.83 | 11.11 |  |
| 27 | 27 | *Paracanthocobitis* | 21 | 3 | 0.83 | 14.29 | 12 |
| 28 | 54 | *Systomus* | 15 | 3 | 0.83 | 20.00 | 34 |
| 29 | 81 | *Esomus* | 9 | 3 | 0.83 | 33.33 | 22 |
| 30 | 85 | *Eutropiichthys* | 7 | 3 | 0.83 | 42.86 |  |
| 31 | 89 | *Batasio* | 18 | 3 | 0.83 | 16.67 | 29 |
| 32 | 91 | *Mystus* | 50 | 3 | 0.83 | 6.00 | 13 |
| 33 | 100 | *Gagata* | 8 | 3 | 0.83 | 37.50 | 38 |
| 34 | 106 | *Pseudecheneis* | 20 | 3 | 0.83 | 15.00 | 23 |
| 35 | 113 | *Clarias* | 63 | 3 | 0.83 | 4.76 |  |
| 36 | 136 | *Trichogaster* | 4 | 3 | 0.83 | 75.00 |  |
| 37 | 143 | *Toxotes* | 10 | 3 | 0.83 | 30.00 |  |
|  |  | **Subtotal** | **1682** | **189** | **52.21** |  |  |

Genus No. corresponds to numbers in Supplementary Appendix 1-1. The Cypriniformes comprise 20 genera, *Schistura*, *Neolissochilus*, *Poropuntius*, *Devario*, *Garra*, *Labeo*, *Danio*, *Lepidocephalichthys*, *Tor*, *Opsarius*, *Botia*, *Physoschistura*, *Triplophysa*, *Puntius*, *Schizothorax*, *Balitora*, *Homatula*, *Paracanthocobitis*, *Systomus*, and *Esomus*. The Siluriformes comprise 10 genera, *Glyptothorax*, *Amblyceps*, *Oreoglanis*, *Exostoma*, *Eutropiichthys*, *Batasio*, *Mystus*, *Gagata*, *Pseudecheneis*, and *Clarias*. The Anabantiformes comprise two genera, *Channa* and *Trichogaster*. The Synbranchiformes comprises a single genus, *Mastacembelus*. The other four genera, *Parambassis*, *Oryzias*, *Anguilla*,and *Toxotes*, belong to other orders.

#### Table S4-2 The fish genera with four species or more from the Irrawaddy river

| Tab. 5 No. | Genus No. | Genus | Species | | % of total species of Irrawaddy River | VFFP for genus | No. in Salween River |
| --- | --- | --- | --- | --- | --- | --- | --- |
|  |  |  | Total | Irrawaddy River |  |  |  |
| 1 | 39 | *Garra* | 189 | 25 | 4.98 | 13.23 | 5 |
| 2 | 35 | *Schistura* | 235 | 23 | 4.58 | 9.79 | 1 |
| 3 | 48 | *Pethia* | 46 | 19 | 3.78 | 41.30 |  |
| 4 | 70 | *Devario* | 44 | 15 | 2.99 | 34.09 | 5 |
| 5 | 94 | *Glyptothorax* | 119 | 15 | 2.99 | 12.61 | 3 |
| 6 | 36 | *Psilorhynchus* | 32 | 14 | 2.79 | 43.75 |  |
| 7 | 58 | *Schizothorax* | 70 | 14 | 2.79 | 20.00 | 21 |
| 8 | 40 | *Labeo* | 112 | 13 | 2.59 | 11.61 | 9 |
| 9 | 68 | *Danio* | 26 | 11 | 2.19 | 42.31 | 10 |
| 10 | 154 | *Channa* | 53 | 10 | 1.99 | 18.87 | 8 |
| 11 | 22 | *Lepidocephalichthys* | 19 | 9 | 1.79 | 47.37 | 11 |
| 12 | 32 | *Paracanthocobitis* | 21 | 9 | 1.79 | 42.86 | 27 |
| 13 | 83 | *Mystus* | 50 | 9 | 1.79 | 18.00 | 32 |
| 14 | 30 | *Mustura* | 18 | 8 | 1.59 | 44.44 |  |
| 15 | 63 | *Opsarius* | 32 | 8 | 1.59 | 25.00 | 13 |
| 16 | 97 | *Exostoma* | 20 | 7 | 1.39 | 35.00 | 22 |
| 17 | 88 | *Amblyceps* | 23 | 6 | 1.20 | 26.09 | 14 |
| 18 | 141 | *Macrognathus* | 25 | 6 | 1.20 | 24.00 |  |
| 19 | 164 | *Parambassis* | 21 | 6 | 1.20 | 28.57 | 6 |
| 20 | 24 | *Pangio* | 34 | 5 | 1.00 | 14.71 |  |
| 21 | 42 | *Neolissochilus* | 31 | 5 | 1.00 | 16.13 | 2 |
| 22 | 73 | *Esomus* | 9 | 5 | 1.00 | 55.56 | 29 |
| 23 | 101 | *Pseudecheneis* | 20 | 5 | 1.00 | 25.00 | 34 |
| 24 | 142 | *Mastacembelus* | 66 | 5 | 1.00 | 7.58 | 16 |
| 25 | 156 | *Badis* | 26 | 5 | 1.00 | 19.23 |  |
| 26 | 49 | *Puntius* | 44 | 4 | 0.80 | 9.09 | 20 |
| 27 | 54 | *Poropuntius* | 35 | 4 | 0.80 | 11.43 | 4 |
| 28 | 69 | *Danionella* | 5 | 4 | 0.80 | 80.00 |  |
| 29 | 81 | *Batasio* | 18 | 4 | 0.80 | 22.22 | 31 |
| 30 | 157 | *Dario* | 9 | 4 | 0.80 | 44.44 |  |
|  |  | **Subtotal** | **1452** | **277** | **55.18** |  |  |

Genus No. corresponds to numbers in Supplementary Appendix 1-2. The Cypriniformes comprise 18 genera, *Garra*, *Schistura*, *Pethia*, *Devario*, *Psilorhynchus*, *Schizothorax*, *Labeo*, *Danio*, *Lepidocephalichthys*, *Paracanthocobitis*, *Mustura*, *Opsarius*, *Pangio*, *Neolissochilus*, *Esomus*, *Puntius*, *Poropuntius*, and *Danionella*. The Siluriformes comprise six genera, *Glyptothorax*, *Mystus*, *Exostoma*, *Amblyceps*, *Pseudecheneis*, and *Batasio*. The Anabantiformes comprise three genera, *Channa*, *Badis*, and *Dario*. The Synbranchiformes comprise two genera, *Macrognathus* and *Mastacembelus*. The genus *Parambassis* belongs to order Cichliformes.
